# Trends of national and sub-national burden attributed to kidney dysfunction risk factor in Iran: 1990-2019

**DOI:** 10.3389/fendo.2023.1115833

**Published:** 2023-02-27

**Authors:** Seyed Aria Nejadghaderi, Sahar Saeedi Moghaddam, Mohammad Keykhaei, Parnian Shobeiri, Negar Rezaei, Nazila Rezaei, Mohsen Naghavi, Bagher Larijani, Farshad Farzadfar

**Affiliations:** ^1^ Non-Communicable Diseases Research Center, Endocrinology and Metabolism Population Sciences Institute, Tehran University of Medical Sciences, Tehran, Iran; ^2^ School of Medicine, Shahid Beheshti University of Medical Sciences, Tehran, Iran; ^3^ Kiel Institute for the World Economy, Kiel, Germany; ^4^ Students’ Scientific Research Center (SSRC), Tehran University of Medical Sciences, Tehran, Iran; ^5^ Faculty of Medicine, Tehran University of Medical Sciences, Tehran, Iran; ^6^ Endocrinology and Metabolism Research Center, Endocrinology and Metabolism Clinical Sciences Institute, Tehran University of Medical Sciences, Tehran, Iran; ^7^ Institute for Health Metrics and Evaluation, University of Washington, Seattle, WA, United States; ^8^ Department of Health Metrics Sciences, School of Medicine, University of Washington, Seattle, WA, United States

**Keywords:** DALYs, Global Burden of Disease, Iran, kidney dysfunction, mortality risk factor, chronic kidney disease

## Abstract

**Background:**

Kidney dysfunction is a risk factor for cardiovascular disease and chronic kidney disease. Herein, we aimed to describe the attributable burden of kidney dysfunction at the national and sub-national levels in Iran.

**Methods:**

The Global Burden of Disease (GBD) 2019 data were extracted on the deaths, disability-adjusted life years (DALYs), years of life lost, and years lived with disability attributed to the risk factor of kidney dysfunction by age and sex at the national and provincial levels from 1990-2019. Also, risk exposure was reported by summary exposure value (SEV) with a range of 0 to 100. The estimated values were based on a comparative risk assessment framework.

**Results:**

In 2019, the age-standardized death rate and age-standardized DALYs rate attributable to kidney dysfunction were 58.2 (95% uncertainty interval of 48.8-68.1) and 1127.2 (981.1-1282.7) per 100,000 population in Iran, respectively. Also, the Sistan and Baluchistan province (1729.3 [1478.3-2006.4]) and the province of Tehran (681.9 [571.4-809.8]) had the greatest and lowest age-standardized DALYs rates, respectively. Nationally, SEVs increased from 22.8 to 26.2. The age-standardized burden attributable to kidney dysfunction had a positive association with age advancement. The attributable age-standardized deaths and DALYs rates in all socio-demographic index regions decreased from 1990-2019. Also, the highest and lowest attributable age-standardized DALYs rates of kidney dysfunction came from ischemic heart disease and peripheral artery disease in 2019, respectively.

**Conclusion:**

Although the attributed age-standardized DALYs and death rates decreased from 1990-2019, risk exposure increased and remains a crucial risk factor in Iran. Therefore, policymakers should consider preparing a preventive program that takes into account different levels of prevention of kidney dysfunction.

## Introduction

Kidney dysfunction is a risk factor for many diseases such as cardiovascular diseases, chronic kidney disease, and even cancers (1–5). Kidney dysfunction accounted for 76.5 million disability-adjusted life years (DALYs) and 3.1 million deaths in 2019 worldwide (6). Also, the risk exposure for kidney dysfunction showed a significant annual percent change of 0.35% between 1990 and 2019 (7). The diagnosis and treatment of kidney dysfunction in its early stages, especially among individuals with pre-existing diabetes, can prevent further life-threatening consequences and medical costs, and can increase quality-adjusted life years (8, 9).

As a middle-income country in the Middle East region, Iran has faced emerging challenges in the path toward overcoming non-communicable diseases; 78.1% of all diseases burden were attributed to such diseases in 2019 (10). According to a meta-analysis conducted on studies up to the end of 2017, the prevalence of chronic kidney disease (CKD) in the Iranian general population was 15.1%, which was greater than the global average (11). The prevalence of CKD was 1.7 times greater in females than males in Iran (18.8% vs. 10.8%) (11). Furthermore, more than 1,145,000 DALYs were attributable to CKD and the highest numbers were due to unknown etiologies, diabetes mellitus, and hypertension, respectively (12). The prevalence of CKD is increasing in Iran, similar to the global trend, and imposing high levels of morbidity and costs (13, 14).

Many studies have reported the burden of kidney disorders, especially CKD, and associated risk factors in national and provincial levels in Iran (12, 15–18). Also, a recent study reported the burden of different diseases in Iran in 2019 and its changes from 1990-2019 (19). To the best of our knowledge, impaired kidney function as a risk factor for different diseases has not been evaluated. As a result, we aimed to report the most recent data on the attributable burden of kidney dysfunction as a risk factor in Iran and its 31 provinces from 1990 to 2019 based on the findings of the Global Burden of Disease (GBD) 2019 study.

## Methods

### Overview

We used the national and sub-national data on the burden of kidney dysfunction in Iran from 1990-2019 provided by the GBD project. The GBD study is a comprehensive project that measures the burden of injuries and both communicable and non-communicable diseases. In the GBD 2019 study, data on the burden of 369 injuries and diseases and 87 risk factors in 204 countries and territories between 1990 and 2019 were provided (7, 20). The details of the methodology used for the estimation of the burden of diseases and risk factors are available elsewhere (7, 20). The GBD study was in accordance with the Guidelines for Accurate and Transparent Health Estimates Reporting (GATHER) (21).

### Data sources

A comparative risk assessment (CRA) framework was used in the GBD study in which the risk factors were categorized into four levels based on common features of each risk factor. Briefly, the CRA framework utilized a six-step comparative risk assessment process to estimate the burden of risk factors: 1. Risk-outcome pair inclusion; 2. Exposure risk estimation; 3. Exposure level estimation; 4. Determining the counterfactual level of exposure; 5. Estimation of theoretical minimum risk exposure level value; and 6. Calculation of population-attributable fractions. Kidney dysfunction was a metabolic risk factor. Also, the burden of diseases attributable to kidney dysfunction, including cardiovascular diseases, chronic kidney disease, and gout, was evaluated (7). The estimated deaths, DALYs, years of life lost (YLLs), and years lived with disability (YLDs) associated with kidney dysfunction as a risk factor, and cause-specific attributable burdens in males, females, and both sexes in eight age groups in Iran and 31 provinces from 1990 to 2019 were utilized. Details on the results of the present study were deposited online at http://ghdx.healthdata.org/gbd-results-tool (22).

### Definitions

Kidney dysfunction is defined as an estimated glomerular filtration rate (eGFR) of less than 60 ml/min/1·73m2 or an albumin-to-creatinine ratio (ACR) greater than or equal to 30 mg/g. Based on urinary ACR and eGFR, kidney dysfunction is divided into four categories, including:

- Stage 1 and 2 chronic kidney disease (CKD): Albuminuria with preserved eGFR (ACR >30 mg/g and eGFR >=60 ml/min/1.73m2)
- Stage 3 CKD: eGFR of 30-59 ml/min/1.73m2
- Stage 4 CKD: eGFR of 15-29 ml/min/1.73m2
- Stage 5 CKD: eGFR<15ml/min/1.73m2, not yet on renal replacement therapy (7).

Gout was defined as the presence of monosodium urate (MSU) crystals or a tophus in addition to at least six out of twelve gout findings proposed by the American College of Rheumatology (20). Ischemic heart disease was included as acute myocardial infarction or chronic ischemic heart disease (i.e., angina and asymptomatic ischemic heart disease following myocardial infarction) (20). Ischemic stroke was defined as “an episode of neurological dysfunction caused by focal cerebral, spinal, or retinal infarction” (20). The definition of peripheral artery disease was an ankle-brachial index (ABI) of less than 0.9 (20). Intracerebral hemorrhage was defined as “a focal collection of blood within the brain parenchyma or ventricular system that is not caused by trauma” (20).

The socio-demographic index (SDI) is a composite measure of the level of development which includes incomes per capita, average educational attainment for those aged ≥15, and total fertility rates under the age of 25. It ranges from 0 (low development) to 1 (high development) or is classified into five quintiles, including low, low-middle, middle, high-middle, and high (23).

The summary exposure value (SEV) is “a measure of a population’s exposure to a risk factor that takes into account the extent of exposure by risk level and the severity of that risk’s contribution to disease burden”. It takes a value from 0% (no excess risk exposure) to 100% (the highest risk exposure) (24). The SEV is calculated by the following formula:


$$SEV=\frac{\int_{x=l}^{u}RR\left(x\right)P\left(x\right)d\left(x\right)-1}{RRmax-1}$$

Where RR(x) is risk ratio at level x of exposure, RR_max_ is the highest risk ratio where more than 1% of population are exposed, P(x) is the density of exposure, and l and u are the lowest and highest levels of exposure, respectively.

### Data processing and statistical analysis

In order to describe the time trend of attributable burden to kidney dysfunction, age-standardized rates and their estimated percentage changes were calculated from 1990 to 2019 for deaths, DALYs, YLLs, and YLDs. We classified age into eight categories, including<20, 20-54, 55-59, 60-64, 65-69, 70-74, 75-79, and 80 plus. The age-standardized rates have been presented as per 100,000 population. For each point estimate, the 95% uncertainty interval (95% UI) has also been presented. The uncertainty intervals (UIs) were defined as the 25th and 975th values of the ordered draws. The R programming software version 3.6.1 (R Foundation for Statistical Computing, Vienna, Austria) was used for implementing statistical analysis (25).

## Results

### National attributed burden

In 2019, the age-standardized death rate (ASDR) and age-standardized DALYs rate attributable to kidney dysfunction were 58.2 (95% UI: 48.8 to 68.1) and 1127.2 (981.1 to 1282.7) per 100,000 population in Iran, respectively. By sex, the ASDR in females decreased from 75.1 (61.5 to 89.7) to 58.2 (48.0 to 68.7) and in males from 80.8 (66.8 to 96.5) to 58.4 (49.0 to 68.8) between 1990 and 2019. Moreover, the age-standardized DALYs rates attributable to kidney dysfunction were 1077.5 (933.4 to 1226.1) and 1179.4 (1023.7 to 1351.6) per 100,000 population in females and males in 2019, respectively (**Table 1**).

**Table 1:** Number and age-standardized rates of deaths, disability-adjusted-life-years (DALYs), years of life lost (YLLs), and years lived with disability (YLDs) attributable to kidney dysfunction in 1990 and 2019 and the overall percentage changes from 1990-2019 in Iran.

Measure	Age, Metric	Year						% Change (1990 to 2019)		
		1990			2019					
		Both	Female	Male	Both	Female	Male	Both	Female	Male
Deaths	Attributed all ages number	15111 (12902 to 17507)	6868 (5843 to 7973)	8242 (6922 to 9598)	35987 (30559 to 41889)	17356 (14522 to 20245)	18631 (15793 to 21799)	138.2 (116.9 to 159.1)	152.7 (126.4 to 180.9)	126 (103.9 to 148.1)
	Attributed age-standardized rate (per 100,000)	78.4 (65.0 to 93.1)	75.1 (61.5 to 89.7)	80.8 (66.8 to 96.5)	58.2 (48.8 to 68.1)	58.2 (48.0 to 68.7)	58.4 (49.0 to 68.8)	-25.8 (-31.7 to -20.5)	-22.4 (-30.6 to -12.9)	-27.8 (-33.7 to -22.0)
DALYs	Attributed all ages number	445178 (387847 to 502678)	200663 (176741 to 226572)	244514 (208327 to 281379)	790836 (692731 to 897947)	368995 (321289 to 415886)	421841 (367827 to 483023)	77.6 (64.2 to 91.3)	83.9 (69.6 to 100.3)	72.5 (57.0 to 90.0)
	Attributed age-standardized rate (per 100,000)	1596.7 (1377.8 to 1837.6)	1502.4 (1297.0 to 1733.1)	1673.4 (1419.8 to 1941.1)	1127.2 (981.1 to 1282.7)	1077.5 (933.4 to 1226.1)	1179.4 (1023.7 to 1351.6)	-29.4 (-33.9 to -24.7)	-28.3 (-33.9 to -22.0)	-29.5 (-35 to -24.0)
YLLs	Attributed all ages number	394008 (341358 to 447212)	172332 (150351 to 195320)	221675 (186173 to 255711)	662716 (579714 to 752950)	301612 (262948 to 341511)	361104 (314310 to 414065)	68.2 (55 to 82.8)	75.0 (58.6 to 93.2)	62.9 (46.9 to 81.1)
	Attributed age-standardized rate (per 100,000)	1445.4 (1233.3 to 1674.7)	1332.8 (1140.1 to 1542.8)	1540.5 (1297.0 to 1794.3)	959.0 (833.1 to 1097.4)	900.9 (773.5 to 1031.8)	1019.4 (881.4 to 1171)	-33.6 (-38.2 to -28.9)	-32.4 (-38.3 to -25.5)	-33.8 (-39.5 to -28.3)
YLDs	Attributed all ages number	51170 (37040 to 66931)	28331 (20733 to 37146)	22839 (16492 to 29802)	128120 (93566 to 166807)	67383 (49342 to 87263)	60737 (43975 to 80304)	150.4 (131.6 to 170.3)	137.8 (119.6 to 157.4)	165.9 (143.9 to 190.7)
	Attributed age-standardized rate (per 100,000)	151.3 (112.5 to 197.2)	169.6 (125.6 to 219.8)	132.9 (97.7 to 173.7)	168.1 (123.8 to 219.8)	176.6 (130.4 to 231.0)	160.0 (116.9 to 210.6)	11.1 (5.6 to 16.9)	4.1 (-2.3 to 10.5)	20.3 (13.9 to 26.2)

Data in parentheses are 95% Uncertainty Intervals (95% UIs).

DALYs, disability-adjusted life years; YLLs, years of life lost; YLDs, years lived with disability.

From 1990 to 2019, both age-standardized DALYs rates and the ASDR were decreased by -29.4% (-33.9% to -24.7%) and -25.8% (-31.7% to -20.5%), respectively. There was a higher decrease among men than women in ASDR (-27.8% [-33.7% to -22.0%] vs. -22.4% [-30.6% to -12.9%]) and age-standardized DALYs rates (-29.5% [-35.0% to -24.0%] vs. -28.3% [-33.9% to -22.0%]), which were not statistically significant (**Table 1**). Over 1990-2019, there was an overall decreasing trend in the age-standardized DALY and death rates (**Figure S1**).

### Provincial attributed burden

**Figure 1:**
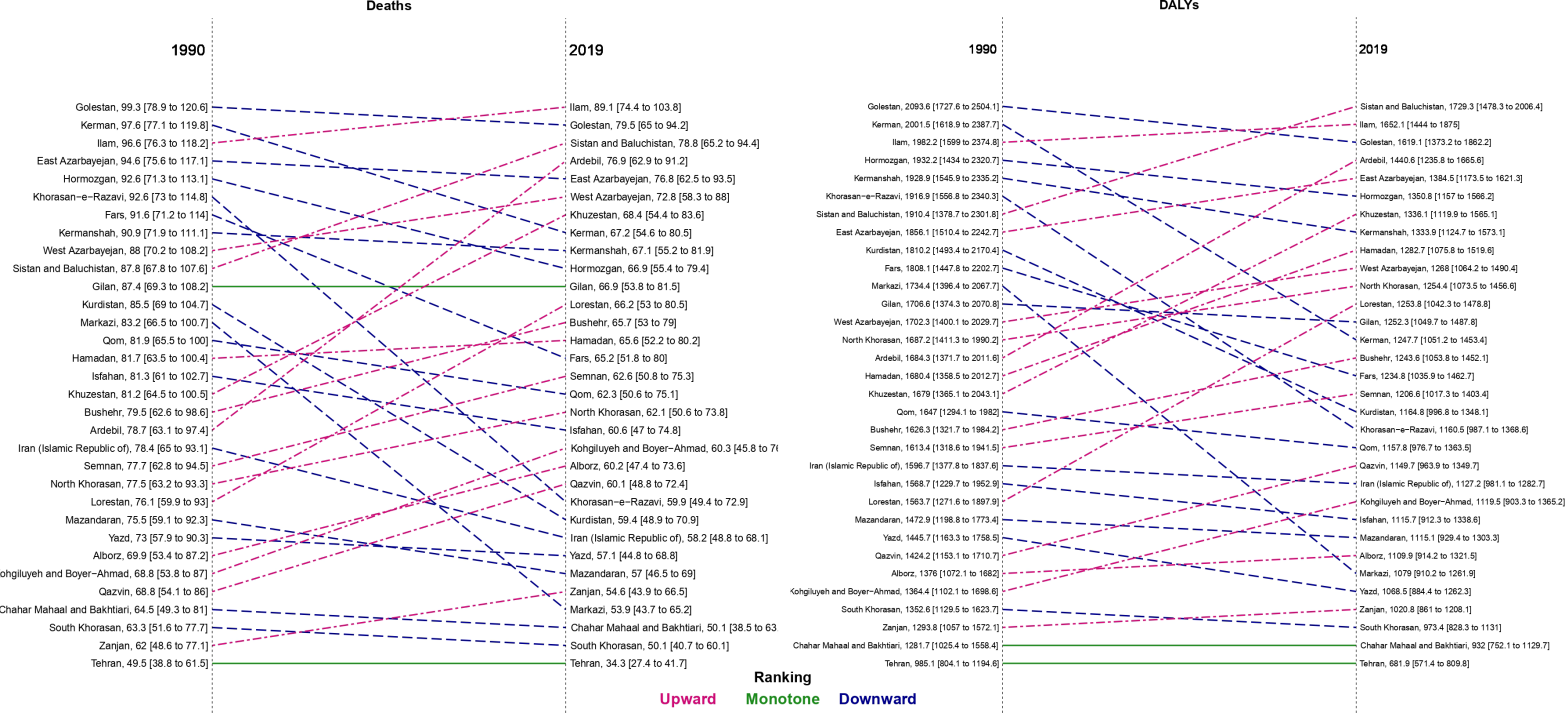
Comparison of the age-standardized rate of deaths and disability-adjusted life years (DALYs) attributable to kidney dysfunction for both sexes between 1990 and 2019 in Iran by province.

In 1990, the provinces of Golestan, Kerman, and Ilam had the highest ASDR and age-standardized DALYs rates in Iran (**Figure 1**; **Figure S2**). Ilam, Golestan, and Sistan and Baluchistan had the highest ASDR values in 2019 of 89.1 (74.4 to 103.8), 79.5 (65.0 to 94.2), and 78.8 (65.2 to 94.4), respectively. Moreover, the highest age-standardized DALYs rates in 2019 were in the provinces of Sistan and Baluchistan (1729.3 [1478.3 to 2006.4]), Ilam (1652.1 [1444.0 to 1875.0]), and Golestan (1619.1 [1373.2 to 1862.2]), respectively (**Figures 1** and **S3**). On the other hand, Tehran had the lowest values of ASDR and age-standardized DALYs rates in females, males, and both sexes in 1990 and 2019 (**Figure 1**; **Table S1**).

Among females, the Golestan province had the highest ASDR (93.3 [71.9 to 116.0]) and age-standardized DALYs rates (1972.6 [1553.3 to 2405.1) in 1990. Among males, the highest ASDR and age-standardized DALYs rates in 1990 were in Kerman (105.5 [81.3 to 131.3]) and Golestan (2201.8 [1714.5 to 2779.2]), respectively. In 2019, Ilam had the highest ASDR in both males (95.2 [77.2 to 113.2]) and females (81.0 [65.4 to 97.5]). In addition, the largest age-standardized DALYs rates in males (1802.2 [1463.5 to 2211.1]) and females (1662.2 [1357.8 to 1995.7]) were in Sistan and Baluchistan (**Table S1**).

From 1990-2019, Ardebil was the only province that had an increase in ASDR in males (6.0% [-15.6 to 32.6]). The change in age-standardized death rates ranged from -4.7% in Kohgiluyeh and Boyer-Ahmad to -32.4% in Khorasan-e-Razavi over this period in females. Also, the greatest decline in age-standardized DALYs rate values among males and females were in Kerman (-40.1%) and Khorasan-e-Razavi (-38.1%), respectively (**Table S1**; **Figure S4**).

### National and provincial exposure to risk

The risk exposure on a scale from 0 to 100 increased from 22.8 (16.7 to 30.0) in 1990 to 26.2 (19.9 to 33.7) in 2019 in Iran. The SEV for females was higher than males in 2019 (28.1 [21.5 to 35.7] vs. 24.4 [18.4 to 31.7]). The trend for kidney dysfunction risk exposure from 1990 to 2019 showed an increase of 15.0% (10.7% to 20.9%) in Iran (**Table 2**).

**Table 2:** Summary exposure values (SEVs) attributable to kidney dysfunction in 1990 and 2019 and the overall percentage changes from 1990-2019 in Iran by province.

Location	Year						% Change (1990 to 2019)		
	1990			2019					
	Both	Female	Male	Both	Female	Male	Both	Female	Male
Iran (Islamic Republic of)	22.8 (16.7 to 30.0)	24.7 (18.2 to 32.3)	21.0 (15.2 to 28.2)	26.2 (19.9 to 33.7)	28.1 (21.5 to 35.7)	24.4 (18.4 to 31.7)	15.0 (10.7 to 20.9)	13.9 (8.8 to 20.6)	16.2 (11.7 to 22)
Alborz	22.1 (16.2 to 29.4)	24.7 (18.2 to 32.3)	19.9 (14.3 to 26.8)	25.8 (19.7 to 33.2)	28.1 (21.5 to 35.7)	23.7 (17.8 to 30.9)	16.7 (11.7 to 23.9)	13.9 (8.8 to 20.6)	19.4 (12.7 to 28.4)
Ardebil	19.9 (14.3 to 26.8)	19.9 (14.3 to 26.8)	19.9 (14.3 to 26.8)	24.5 (18.3 to 31.9)	24.5 (18.3 to 31.9)	24.5 (18.3 to 31.9)	22.8 (15.2 to 32.4)	22.8 (15.2 to 32.4)	22.8 (15.2 to 32.4)
Bushehr	19.9 (14.4 to 27.1)	19.9 (14.4 to 27.1)	19.9 (14.4 to 27.1)	23.6 (17.7 to 30.8)	23.6 (17.7 to 30.8)	23.6 (17.7 to 30.8)	18.6 (11.7 to 26.9)	18.6 (11.7 to 26.9)	18.6 (11.7 to 26.9)
Chahar Mahaal and Bakhtiari	18.6 (13.2 to 25.5)	18.6 (13.2 to 25.5)	18.6 (13.2 to 25.5)	22.1 (16.2 to 29.3)	22.1 (16.2 to 29.3)	22.1 (16.2 to 29.3)	18.5 (12.1 to 27.7)	18.5 (12.1 to 27.7)	18.5 (12.1 to 27.7)
East Azarbayejan	20.9 (15.2 to 28.1)	20.9 (15.2 to 28.1)	20.9 (15.2 to 28.1)	25.2 (19.1 to 32.8)	25.2 (19.1 to 32.8)	25.2 (19.1 to 32.8)	20.7 (13.7 to 30.7)	20.7 (13.7 to 30.7)	20.7 (13.7 to 30.7)
Fars	22.6 (16.7 to 29.8)	22.6 (16.7 to 29.8)	22.6 (16.7 to 29.8)	25.7 (19.4 to 33.2)	25.7 (19.4 to 33.2)	25.7 (19.4 to 33.2)	13.5 (8.1 to 20.4)	13.5 (8.1 to 20.4)	13.5 (8.1 to 20.4)
Gilan	20.1 (14.4 to 27.3)	20.1 (14.4 to 27.3)	20.1 (14.4 to 27.3)	24.0 (18.1 to 31.3)	24.0 (18.1 to 31.3)	24.0 (18.1 to 31.3)	19.2 (12.1 to 29.1)	19.2 (12.1 to 29.1)	19.2 (12.1 to 29.1)
Golestan	23.2 (17.3 to 30.3)	23.2 (17.3 to 30.3)	23.2 (17.3 to 30.3)	26.1 (20.1 to 33.6)	26.1 (20.1 to 33.6)	26.1 (20.1 to 33.6)	12.5 (7.2 to 19.7)	12.5 (7.2 to 19.7)	12.5 (7.2 to 19.7)
Hamadan	19.6 (14.1 to 26.7)	19.6 (14.1 to 26.7)	19.6 (14.1 to 26.7)	23.7 (17.7 to 31.1)	23.7 (17.7 to 31.1)	23.7 (17.7 to 31.1)	20.7 (13.8 to 30.8)	20.7 (13.8 to 30.8)	20.7 (13.8 to 30.8)
Hormozgan	19.7 (14.0 to 26.7)	19.7 (14.0 to 26.7)	19.7 (14.0 to 26.7)	24.5 (18.3 to 32.2)	24.5 (18.3 to 32.2)	24.5 (18.3 to 32.2)	24.3 (16.6 to 34.2)	24.3 (16.6 to 34.2)	24.3 (16.6 to 34.2)
Ilam	21.3 (15.5 to 28.4)	21.3 (15.5 to 28.4)	21.3 (15.5 to 28.4)	26.3 (20.1 to 33.9)	26.3 (20.1 to 33.9)	26.3 (20.1 to 33.9)	23.5 (15.9 to 33.8)	23.5 (15.9 to 33.8)	23.5 (15.9 to 33.8)
Isfahan	27.4 (19.9 to 35.5)	27.4 (19.9 to 35.5)	27.4 (19.9 to 35.5)	29.7 (22.1 to 38.0)	29.7 (22.1 to 38.0)	29.7 (22.1 to 38.0)	8.6 (4.2 to 13.8)	8.6 (4.2 to 13.8)	8.6 (4.2 to 13.8)
Kerman	21.0 (15.3 to 28.2)	21.0 (15.3 to 28.2)	21.0 (15.3 to 28.2)	24.5 (18.3 to 31.9)	24.5 (18.3 to 31.9)	24.5 (18.3 to 31.9)	16.5 (10.9 to 24.1)	16.5 (10.9 to 24.1)	16.5 (10.9 to 24.1)
Kermanshah	20.0 (14.3 to 27.2)	20.0 (14.3 to 27.2)	20.0 (14.3 to 27.2)	24.1 (18 to 31.5)	24.1 (18.0 to 31.5)	24.1 (18.0 to 31.5)	20.6 (14.0 to 29.7)	20.6 (14.0 to 29.7)	20.6 (14 to 29.7)
Khorasan-e-Razavi	20.8 (15.1 to 28.1)	20.8 (15.1 to 28.1)	20.8 (15.1 to 28.1)	23.4 (17.5 to 30.6)	23.4 (17.5 to 30.6)	23.4 (17.5 to 30.6)	12.1 (6.5 to 19.4)	12.1 (6.5 to 19.4)	12.1 (6.5 to 19.4)
Khuzestan	19.9 (14.2 to 27.1)	19.9 (14.2 to 27.1)	19.9 (14.2 to 27.1)	23.9 (18.0 to 31.3)	23.9 (18.0 to 31.3)	23.9 (18.0 to 31.3)	20.2 (13.6 to 29.9)	20.2 (13.6 to 29.9)	20.2 (13.6 to 29.9)
Kohgiluyeh and Boyer-Ahmad	18.9 (13.3 to 25.9)	18.9 (13.3 to 25.9)	18.9 (13.3 to 25.9)	22.9 (17.1 to 30.0)	22.9 (17.1 to 30.0)	22.9 (17.1 to 30.0)	21.4 (14.3 to 30.8)	21.4 (14.3 to 30.8)	21.4 (14.3 to 30.8)
Kurdistan	19.7 (14.2 to 26.8)	19.7 (14.2 to 26.8)	19.7 (14.2 to 26.8)	23.9 (17.8 to 31.0)	23.9 (17.8 to 31.0)	23.9 (17.8 to 31.0)	20.8 (14.1 to 29.8)	20.8 (14.1 to 29.8)	20.8 (14.1 to 29.8)
Lorestan	19.4 (14.0 to 26.5)	19.4 (14.0 to 26.5)	19.4 (14.0 to 26.5)	23.7 (17.8 to 31.3)	23.7 (17.8 to 31.3)	23.7 (17.8 to 31.3)	22.3 (15.2 to 32.5)	22.3 (15.2 to 32.5)	22.3 (15.2 to 32.5)
Markazi	20.7 (14.9 to 27.9)	20.7 (14.9 to 27.9)	20.7 (14.9 to 27.9)	24.3 (18.2 to 31.8)	24.3 (18.2 to 31.8)	24.3 (18.2 to 31.8)	17.3 (11.4 to 25.2)	17.3 (11.4 to 25.2)	17.3 (11.4 to 25.2)
Mazandaran	20.5 (14.8 to 27.6)	20.5 (14.8 to 27.6)	20.5 (14.8 to 27.6)	24.2 (18.2 to 31.8)	24.2 (18.2 to 31.8)	24.2 (18.2 to 31.8)	18.3 (11.8 to 26.9)	18.3 (11.8 to 26.9)	18.3 (11.8 to 26.9)
North Khorasan	19.4 (13.8 to 26.3)	19.4 (13.8 to 26.3)	19.4 (13.8 to 26.3)	23.6 (17.5 to 31.0)	23.6 (17.5 to 31.0)	23.6 (17.5 to 31.0)	21.8 (14.9 to 31.3)	21.8 (14.9 to 31.3)	21.8 (14.9 to 31.3)
Qazvin	19.4 (13.9 to 26.5)	19.4 (13.9 to 26.5)	19.4 (13.9 to 26.5)	23.9 (17.9 to 31.3)	23.9 (17.9 to 31.3)	23.9 (17.9 to 31.3)	23.5 (15.7 to 33.8)	23.5 (15.7 to 33.8)	23.5 (15.7 to 33.8)
Qom	20.3 (14.6 to 27.5)	20.3 (14.6 to 27.5)	20.3 (14.6 to 27.5)	24.2 (18.2 to 31.5)	24.2 (18.2 to 31.5)	24.2 (18.2 to 31.5)	19.0 (12.5 to 28.4)	19.0 (12.5 to 28.4)	19.0 (12.5 to 28.4)
Semnan	20.7 (15.0 to 27.5)	20.7 (15.0 to 27.5)	20.7 (15.0 to 27.5)	24.6 (18.5 to 31.7)	24.6 (18.5 to 31.7)	24.6 (18.5 to 31.7)	18.9 (12.5 to 27.7)	18.9 (12.5 to 27.7)	18.9 (12.5 to 27.7)
Sistan and Baluchistan	20.3 (14.7 to 27.4)	20.3 (14.7 to 27.4)	20.3 (14.7 to 27.4)	25.1 (18.8 to 32.7)	25.1 (18.8 to 32.7)	25.1 (18.8 to 32.7)	23.8 (16.3 to 33.6)	23.8 (16.3 to 33.6)	23.8 (16.3 to 33.6)
South Khorasan	19.3 (13.8 to 26.3)	19.3 (13.8 to 26.3)	19.3 (13.8 to 26.3)	23.3 (17.2 to 30.5)	23.3 (17.2 to 30.5)	23.3 (17.2 to 30.5)	20.3 (13.6 to 30.1)	20.3 (13.6 to 30.1)	20.3 (13.6 to 30.1)
Tehran	21.3 (15.6 to 28.3)	21.3 (15.6 to 28.3)	21.3 (15.6 to 28.3)	23.3 (17.5 to 30.6)	23.3 (17.5 to 30.6)	23.3 (17.5 to 30.6)	9.3 (4.7 to 15.5)	9.3 (4.7 to 15.5)	9.3 (4.7 to 15.5)
West Azarbayejan	19.3 (13.8 to 26.3)	19.3 (13.8 to 26.3)	19.3 (13.8 to 26.3)	23.3 (17.4 to 30.5)	23.3 (17.4 to 30.5)	23.3 (17.4 to 30.5)	20.6 (13.7 to 30.8)	20.6 (13.7 to 30.8)	20.6 (13.7 to 30.8)
Yazd	19.9 (14.4 to 26.9)	19.9 (14.4 to 26.9)	19.9 (14.4 to 26.9)	24.3 (18.2 to 31.6)	24.3 (18.2 to 31.6)	24.3 (18.2 to 31.6)	22.0 (14.5 to 32.0)	22.0 (14.5 to 32.0)	22 (14.5 to 32.0)
Zanjan	19.2 (13.6 to 26.3)	19.2 (13.6 to 26.3)	19.2 (13.6 to 26.3)	23.1 (17.0 to 30.4)	23.1 (17.0 to 30.4)	23.1 (17.0 to 30.4)	20.4 (13.8 to 30.1)	20.4 (13.8 to 30.1)	20.4 (13.8 to 30.1)

Data in parentheses are 95% Uncertainty Intervals (95% UIs).

At the sub-national level, the risk exposure among both sexes in 2019 ranged from 22.1 (16.2 to 29.3) in Chahar Mahaal and Bakhtiari to 29.7 (22.1 to 38.0) in Isfahan. The lowest and highest change in risk exposure between 1990 and 2019 was in Isfahan (8.6% [4.2% to 13.8%]) and Hormozgan (24.3% [16.6% to 34.2%]), respectively (**Table 2**).

### Attributed burden of kidney dysfunction by age

**Figure 2:**
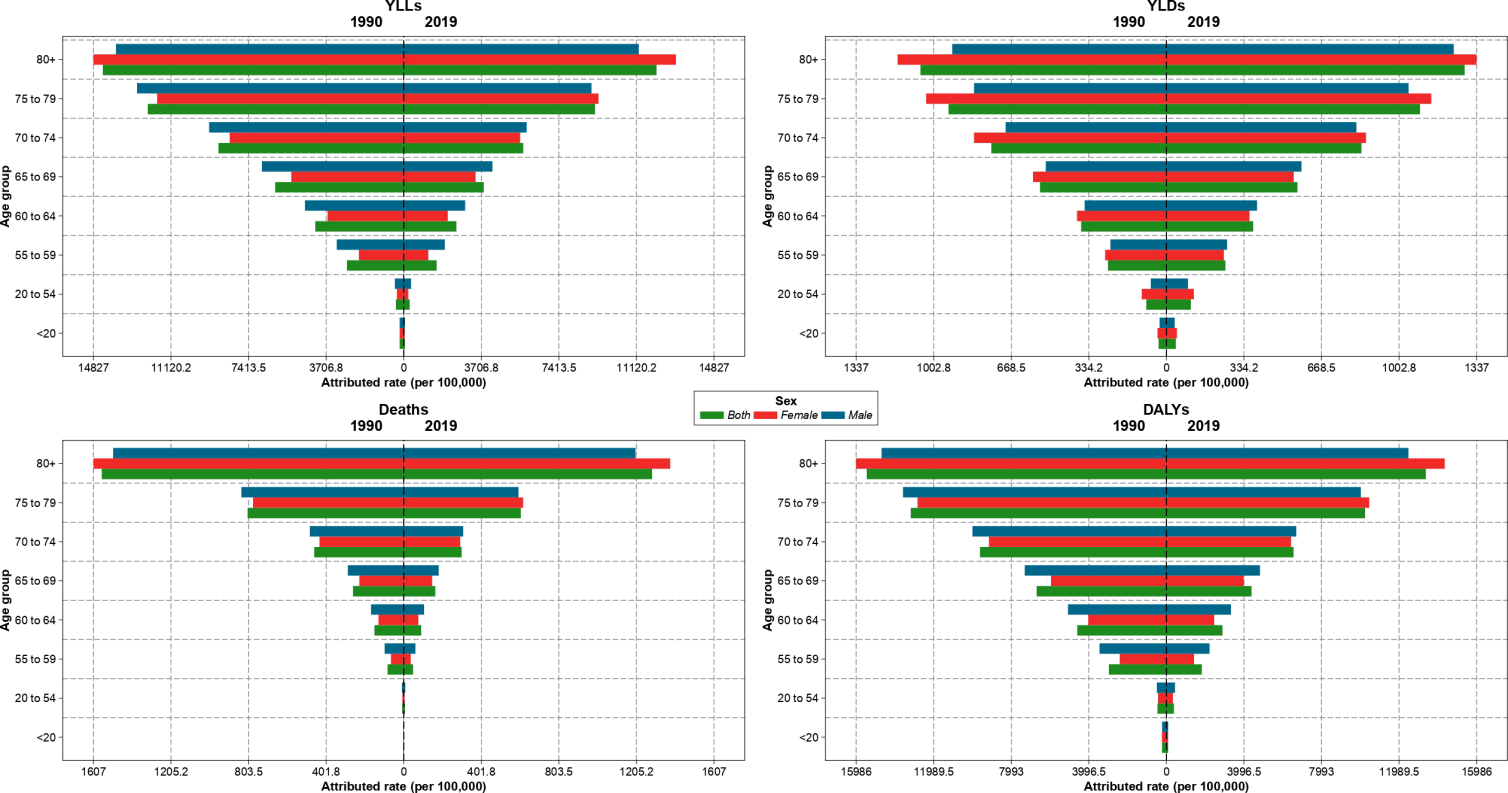
Age-standardized rate of years of life lost (YLLs), years lived with disability (YLDs), deaths, and disability-adjusted life years (DALYs) attributable to kidney dysfunction in Iran in 1990 and 2019 by sex and age.

The attributable age-standardized death, DALYs, YLLs, and YLDs rates per 100,000 population increased with age and had the highest values in the 80-years plus category in 1990 and 2019 in Iran. In 1990, the ASDR and age-standardized DALYs rates were higher in males than females up to 79 years old. While, in 2019, these measures were higher among males from birth up to 74 years old, and females aged 75 or older had higher rates (**Figure 2**). The increasing trends of the burden of age-standardized deaths and DALYs attributable to kidney dysfunction in provinces were similar to the national trend (**Figure S5**).

### Attributed burden of kidney dysfunction by SDI

**Figure 3:**
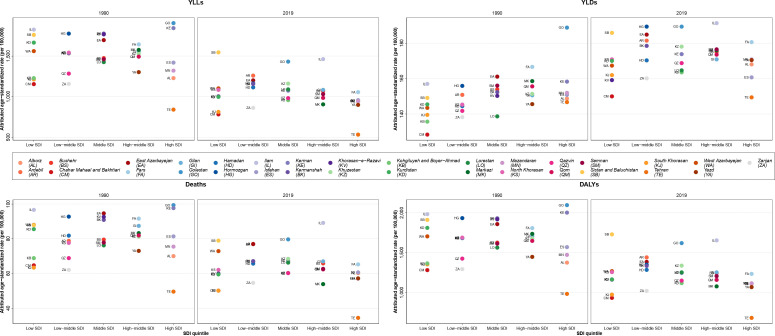
Age-standardized rate of years of life lost (YLLs), years lived with disability (YLDs), deaths, and disability-adjusted life years (DALYs) attributable to kidney dysfunction in Iran in 1990 and 2019 by sociodemographic index (SDI) quintiles and province.

Overall, the ASDR and age-standardized DALYs rate values in 2019 were lower than in 1990 in almost all SDI quintiles. Golestan, with a DALYs rate of above 2,000 per 100,000 population, had the highest age-standardized DALYs rates in 1990 and 2000, whereas it decreased to lower than 2,000 in 2010 and 2019 (**Figure S6**). Furthermore, the highest ASDR, which was in Golestan and Kerman in 1990 and 2019 with almost 100 deaths per 100,000 population in 1990 and 2010, decreased to about 90 deaths per 100,000 population in Ilam in 2019 (**Figure S7**). In 2019, the ASDR in all SDI quintiles were near to each other, whereas the provinces of Tehran and Ilam, which had the lowest and highest ASDR, respectively, were in the high and high-middle SDI quintiles, respectively. Also, Sistan and Baluchistan, which was in the low SDI quintile, had the largest DALYs age-standardized rate (**Figure 3**).

### Burden of diseases attributable to kidney dysfunction

**Figure 4:**
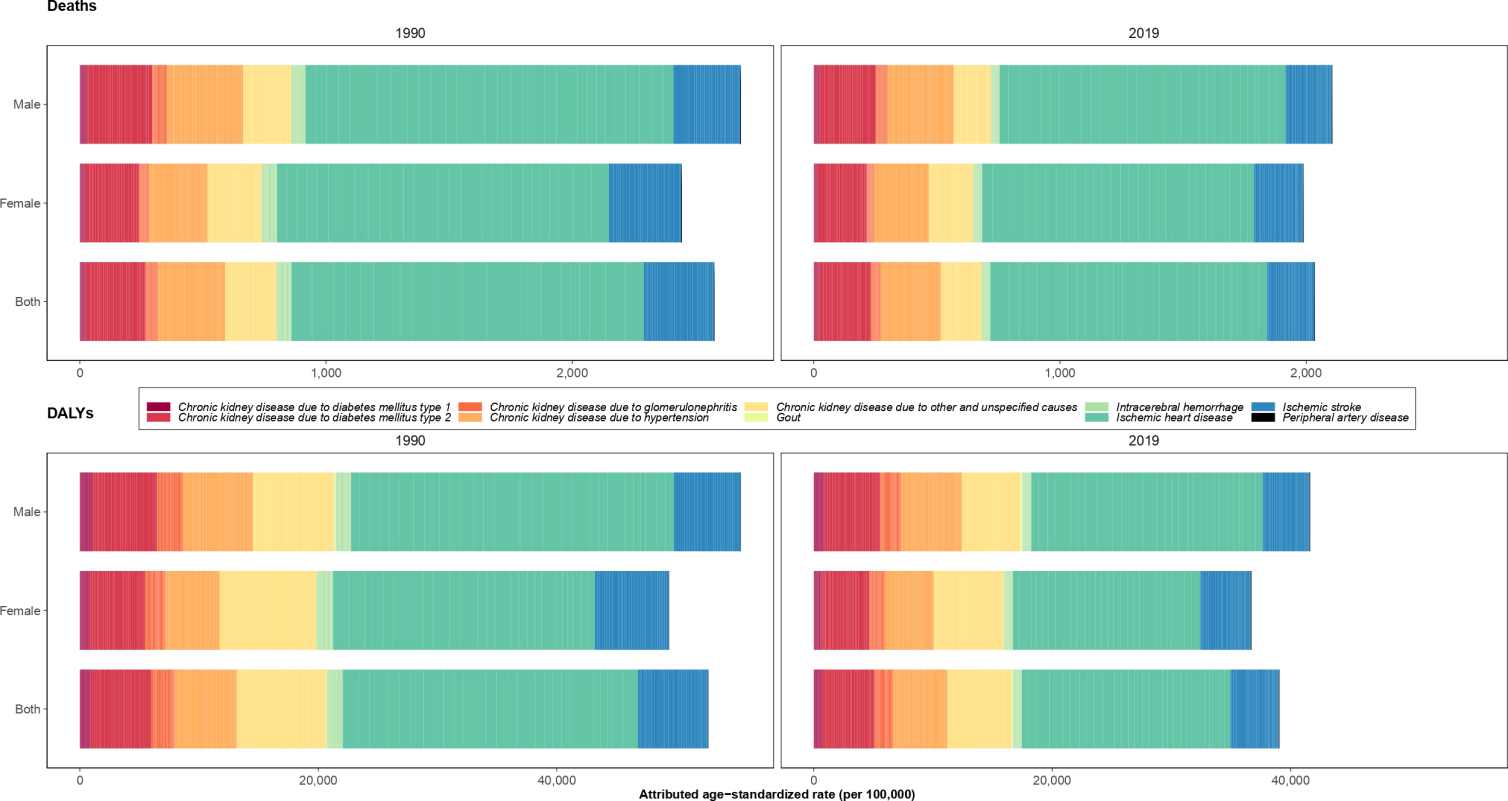
Age-standardized rate of deaths and disability-adjusted life years (DALYs) of underlying causes attributable to kidney dysfunction in 1990 and 2019 in Iran by sex.

The highest attributable age-standardized DALYs rate of kidney dysfunction came from ischemic heart disease (755.6 [559.8-975.5]), while peripheral artery disease (1.3 [0.7-2.2]) had the lowest attributable burden in 1990. Ischemic heart disease and CKD due to other and unspecified causes than kidney diseases had the highest attributable age-standardized DALYs rates among both sexes in Iran in 2019. Furthermore, the largest ASDR of kidney dysfunction was contributed by ischemic heart disease in 1990 and 2019 (43.9 [30.6-58.4] in 1990 and 31.7 [22.7-40.9] in 2019). The second most attributed cause of death was ischemic stroke in 1990 and CKD due to hypertension in 2019 (**Figure 4**).

## Discussion

Our descriptive national and sub-national study on the attributed burden of kidney dysfunction showed that ASDR and age-standardized DALYs rates decreased over the 1990-2019 period, although risk exposure and age-standardized YLD increased. Sistan and Baluchistan, Golestan, and Ilam accounted for the highest attributable burdens in 2019. The attributed age-standardized death and DALYs rates had a positive association with age and were greater in males. Also, the age-standardized death and DALYs rates decreased in all SDI quintiles, while the low-middle SDI had the highest values in 2019. The highest deaths and DALYs resulted from ischemic heart disease.

Comparing the burden of kidney dysfunction in Iran with the regional and global levels in 2019 showed that Iran had lower ASDR and age-standardized DALYs rates than the Eastern Mediterranean Region of the World Health Organization (WHO) classifications (ASDR: 58.2 vs. 87.4 and age-standardized DALYs rate: 1127.2 vs. 1837.0), and also in comparison to North Africa and the Middle East out of 21 GBD regions (ASDR: 58.2 vs. 83.4 and age-standardized DALYs rate: 1127.2 vs. 1691.0). However, the attributable burden in Iran was the highest in the world (ASDR: 58.2 vs. 40.6 and age-standardized DALYs rate: 1127.2 vs. 945.3) (6). The higher burden of kidney dysfunction in comparison to global values could be explained by a greater prevalence of risk factors for chronic kidney disease such as salt consumption, physical inactivity, overweight and obesity, and high blood pressure in Iran (5, 26–29). On the other hand, there were negative changes in the age-standardized DALYs and death rates attributable to kidney dysfunction in Iran, which could be as a result of improving medication and access to healthcare equipment within Iran over the last three decades.

The global SEV for kidney dysfunction increased from 20.6 (14.3 to 28.0) in 1990 to 22.7 (16.2 to 30.3) in 2019 with a significant annualized rate of change of 0.35% over this period (7). Our findings showed an increase from 22.8 to 26.1 from 1990 to 2019 for SEVs in Iran. The higher risk exposure of kidney dysfunction could be justified by a higher risk of exposure to previously mentioned risk factors like salt intake in addition to high fasting blood glucose levels in Iran compared to global levels (27, 30). Also, our results showed that Isfahan and the Chahar Mahaal and Bakhtiari province had the highest and lowest SEVs, respectively. The prevalence of different risk factors attributable to kidney dysfunction, such as metabolic syndrome, high blood pressure, inadequate physical activity, and high levels of salt consumption, had discrepancies between the various provinces. Overall, this variation in the burden of these risk factors in addition to sex and age disparities in the provinces could have led to the discrepancies in SEVs.

Studies that were conducted in different provinces of Iran, including Tehran, Fars, Kerman, and Golestan showed that the burden of CKD was higher in females than males (15, 18, 31–33). On the other hand, the study by Ghafari et al. in urban and rural populations of Urmia revealed no significant difference between males and females in the prevalence of high serum creatinine (p=0.13) and proteinuria (p=0.44) as markers of impairment in kidney function (34). Moreover, a cross-sectional study on 1,400 participants in Shahreza in Isfahan province revealed a higher frequency of micro/macro-albuminuria in females compared to males (16.8% vs. 15.0%) (35). Also, low GFR was greater among females and had a significant positive association with age (35). The Tehran Lipid and Glucose Study in 2009 on individuals aged 20 and over showed a higher prevalence of CKD in the ≥70 years old age group compared to the 20-39 year old group (61.0% vs. 3.5%) (15). In addition, the prevalence rate among females was 23.0% compared to 13.1% in males (15). The article by Khajehdehi et al. in Southern Iran showed a significant higher rate of stage 3-5 CKD in the elderly (p<0.001) and in females compared to males (14.9% vs. 5.4%) (18). Our results showed a higher rate of deaths and DALYs attributable to kidney dysfunction in males up to 74 years old. By increasing oxidative stress, promoting fibrosis, and inducing the activation of the renin-angiotensin-aldosterone system, male hormones are associated with worse CKD progression (36). Therefore, this might justify the trend that measures were higher among males from birth up to 74 years old and in females aged 75 or older.

The findings of studies in Iran about the level of awareness of people about the three diseases of diabetes, blood pressure, and kidney disease have also shown that awareness of the studied population about diabetes and blood pressure was much higher than that of kidney disease, and long-term training concerning diabetes and blood pressure have, to a large extent, not been effective in aspects of prevention and treatment. Thus, more training and information about kidney disease is needed. Therefore, it is necessary to make people, doctors, and health workers aware of these risks, and able to receive and perform simple and low-cost screening tests such as urine tests and creatinine measurements; furthermore, there is need for effective policies for timely diagnosis and treatment to prevent the progression of the disease and its complications. Evidence suggests that the diagnosis of kidney disease and the availability of community centers which provide the point-of-care to identify and guide the management of patients locally are critical factors in kidney disease-related DALY rates (37, 38). Dehghani et al. showed that of 9,781 participants aged 30–73 years-old were referred to community health centers of Iran, and 27.5% had positive screening results for renal dysfunction requiring follow-up (39). There are complex relationships between kidney disease, metabolic factors, and behavioral factors, such as nutritional habits. The results of an ongoing cohort study in Iran have shown that the Iranian dietary pattern is safe and not related to incidences of CKD, but that a high-fat, high-sugar diet may significantly (46%) increase the likelihood of CKD occurrence, whereas a lacto-vegetarian diet may protect against CKD occurrence by 43% (37). A reorientation of food systems appears to be needed to achieve better health and environmental outcomes because of kidney function-related dietary patterns. Shifting to a healthier diet requires that the necessary foods be both available and affordable to low-income populations. Food choices depend on resource availability, cost, and access to quality food in the area.

To our best of knowledge, no study was conducted to explain the association of SDIs and the burden of kidney dysfunction in Iran and its provinces. As a critical insight from this study, we found that low-middle, middle, and high-middle SDI quintiles had higher attributable age-standardized death and DALYs rates, although they were close to other SDI quintiles. Globally, all SDI quintiles had a positive annualized rate of change values for kidney dysfunction, while the highest and lowest DALYs attributable to kidney dysfunction were in the middle and low SDI quintiles, respectively (7). The discrepancies between the provinces of Iran in the measurements of SDI quintiles might be a result of the differences in access to health care facilities. For example, kidney dysfunction might be diagnosed in end-stages, resulting in increased rates of deaths and DALYs (40). In the United States of America, there was a positive upward association between the age-standardized DALYs rates attributable to CKD and SDI from 2002-2016 in national and state levels except in Washington D.C. (41).

Appraising the attributable burden of kidney dysfunction, Bikbov et al. showed that kidney dysfunction accounted for 58.4%, 41.6%, and 0.003% of CKD, cardiovascular disease, and gout, respectively, in 2017 worldwide (5). Also, it was illustrated that ischemic heart disease and peripheral artery disease had the highest and lowest attributable DALYs originating from kidney dysfunction, respectively (5). Other attributable causes of CKD that were represented were diabetes mellitus type 1 and 2, hypertension, and glomerulonephritis. In this regard, an observational study showed that a past history of cardiovascular diseases (OR= 1.47; 95% CI: 1.27-1.69), hypertension (OR= 1.58; 95% CI: 1.43-1.74), and diabetes (OR= 1.09; 95% CI: 1.02-1.23) were associated with CKD development (17). Furthermore, Saber et al. revealed that hypertension in addition to hypercholesterolemia and high low-density lipoprotein (LDL) were significantly associated with CKD (p<0.05), whereas no significant association was found for diabetes mellitus (33).

## Strengths and limitations

A strength of our study was that it was one of the most comprehensive and up-to-date studies to describe the attributable burden to kidney dysfunction by age, sex, location, SDI, and its contributed diseases. Also, a measure indicating the exposure to risk factors called SEV was introduced for kidney dysfunction in Iran and its provinces. Nevertheless, this study had several limitations. First, the lack of data due to under-registration in certain provinces was a major limitation of this study. Second, data on urban and rural areas or various areas of large cities were not available, due to the differences in risk factors between rural and urban areas, they might have affected the interpretation of results (42). It should be considered that most of the limitations were as a result of GBD methodology in data collection and the fact that we could not manipulate this. Nevertheless, the GBD project has one of the most comprehensive and recent datasets on Iran and its provinces in addition to regional and global levels.

## Conclusion

Although the attributed age-standardized DALYs and deaths decreased from 1990-2019, the risk exposure increased and remains a crucial risk factor in Iran. Kidney dysfunction not only due to CKD can increase mortality and morbidity, but also predispose individuals to cardiovascular disease. Therefore, policymakers should consider preparing a preventive program that takes into account different levels of prevention from kidney dysfunction. Moreover, increasing awareness and directing the attention of public health authorities and citizens to such programs could be effective.

## 2019 Iran Kidney Dysfunction Collaborators

Ashkan Abdollahi, Department of Medicine, Johns Hopkins University, Baltimore, MD, United States; Department of Medicine, Shiraz University of Medical Sciences, Shiraz, Iran; Ali Ahmadi, Department of Epidemiology and Biostatistics, Shahrekord University of Medical Sciences, Shahrekord, Iran; Department of Epidemiology, Shahid Beheshti University of Medical Sciences, Tehran, Iran; Sepideh Ahmadi, School of Advanced Technologies in Medicine, Shahid Beheshti University of Medical Sciences, Tehran, Iran; Sudabeh Alatab, Digestive Diseases Research Institute, Tehran University of Medical Sciences, Tehran, Iran; Jalal Arabloo, Health Management and Economics Research Center, Iran University of Medical Sciences, Tehran, Iran; Mohammad Arjomandzadegan, Infectious Diseases Research Center (IDRC), Arak University of Medical Sciences, Arak, Iran; Seyyed Shamsadin Athari, Department of Immunology, Zanjan University of Medical Sciences, Zanjan, Iran; Sina Azadnajafabad, Non-Communicable Diseases Research Center, Endocrinology and Metabolism Population Sciences Institute, Tehran University of Medical Sciences, Tehran, Iran; Mohammadreza Azangou-Khyavy, Non-Communicable Diseases Research Center, Endocrinology and Metabolism Population Sciences Institute, Tehran University of Medical Sciences, Tehran, Iran; Social Determinants of Health Research Center, Shahid Beheshti University of Medical Sciences, Tehran, Iran; Nayereh Baghcheghi, Department of Nursing, Saveh University of Medical Sciences, Saveh, Iran; Sara Bagherieh, School of Medicine, Isfahan University of Medical Sciences, Isfahan, Iran; Shirin Barati, Department of Anatomy, Saveh University of Medical Sciences, Saveh, Iran; Azizallah Dehghan, Department of Epidemiology and Community Medicine, Non-Communicable Diseases Research Center (NCDRC), Fasa, Iran; Ali Fatehizadeh, Department of Environmental Health Engineering, Isfahan University of Medical Sciences, Isfahan, Iran; Fataneh Ghadirian, Psychiatric Nursing and Management Department, Shahid Beheshti University of Medical Sciences, Tehran, Iran; Maryam Gholamalizadeh, Cancer Research Center, Shahid Beheshti University of Medical Sciences, Tehran, Iran; Ali Gholami, Department of Epidemiology and Biostatistics, Neyshabur University of Medical Sciences, Neyshabur, Iran; Non-communicable Diseases Research Center, Neyshabur University of Medical Sciences, Neyshabur, Iran; Kimiya Gohari, Non-Communicable Diseases Research Center, Endocrinology and Metabolism Population Sciences Institute, Tehran University of Medical Sciences, Tehran, Iran; Department of Biostatistics, Tarbiat Modares University, Tehran, Iran; Hadi Hassankhani, School of Nursing and Midwifery, Tabriz University of Medical Sciences, Tabriz, Iran; Independent Consultant, Tabriz, Iran; Mohammad Jokar, Zoonotic Research Center, Islamic Azad University, Tehran, Iran; Department of Clinical Sciences, Jahrom University of Medical Sciences, Jahrom, Iran; Fatemeh Khorashadizadeh, Department of Epidemiology and Biostatistics, Neyshabur University of Medical Sciences, Neyshabur, Iran; Farzad Kompani, Children’s Medical Center, Tehran University of Medical Sciences, Tehran, Iran; Hamid Reza Koohestani, Social Determinants of Health Research Center, Saveh University of Medical Sciences, Saveh, Iran; Soleiman Mahjoub, Cellular and Molecular Biology Research Center, Health Research Institute, Babol University of Medical Sciences, Babol, Iran; Department of Clinical Biochemistry, Babol University of Medical Sciences, Babol, Iran; Ata Mahmoodpoor, Department of Anesthesiology and Critical Care, Tabriz University of Medical Sciences, Tabriz, Iran; Elaheh Malakan Rad, Pediatric Cardiology Unit, Tehran University of Medical Sciences, Tehran, Iran; Mohammadreza Mobayen, Burn and Regenerative Medicine Research Center, Guilan University of Medical Sciences, Rasht, Iran; Esmaeil Mohammadi, Non-Communicable Diseases Research Center, Endocrinology and Metabolism Population Sciences Institute, Tehran University of Medical Sciences, Tehran, Iran; School of Medicine, Tehran University of Medical Sciences, Tehran, Iran; Yousef Moradi, Social Determinants of Health Research Center, Kurdistan University of Medical Sciences, Kurdistan, Iran; Negar Morovatdar, Clinical Research Development Unit, Mashhad University of Medical Sciences, Mashhad, Iran; Maryam Noori, Student Research Committee, Iran University of Medical Sciences, Tehran, Iran; Hassan Okati-Aliabad, Health Promotion Research Center, Zahedan University of Medical Sciences, Zahedan, Iran; Ghazaleh Pourali, Metabolic Syndrome Research Center, Mashhad University of Medical Sciences, Mashhad, Iran; International UNESCO Center for Health-Related Basic Sciences and Human Nutrition, Mashhad University of Medical Sciences, Mashhad, Iran; Quinn Rafferty, Institute for Health Metrics and Evaluation, University of Washington, Seattle, WA, United States; Sina Rashedi, Non-Communicable Diseases Research Center, Endocrinology and Metabolism Population Sciences Institute, Tehran University of Medical Sciences, Tehran, Iran; Department of Cardiology, Tehran University of Medical Sciences, Tehran, Iran; Mahsa Rashidi, Department of Clinical Science, Islamic Azad University, Garmsar, Iran; Mohammad-Mahdi Rashidi, Non-Communicable Diseases Research Center, Endocrinology and Metabolism Population Sciences Institute, Tehran University of Medical Sciences, Tehran, Iran; Social Determinants of Health Research Center, Shahid Beheshti University of Medical Sciences, Tehran, Iran; Amirhossein Sahebkar, Applied Biomedical Research Center, Mashhad University of Medical Sciences, Mashhad, Iran; Biotechnology Research Center, Mashhad University of Medical Sciences, Mashhad, Iran; Seyed Afshin Shorofi, Medical-Surgical Nursing Department, Mazandaran University of Medical Sciences, Sari, Iran; Department of Nursing and Health Sciences, Flinders University, Adelaide, SA, Australia; Seyyed Mohammad Tabatabaei, Department of Medical Informatics, Mashhad University of Medical Sciences, Mashhad, Iran; Clinical Research Development Unit, Mashhad University of Medical Sciences, Mashhad, Iran; Majid Taheri, Trauma and Injury Research Center, Iran University of Medical Sciences, Tehran, Iran; Medical Ethics and Law Research Center, Shahid Beheshti University of Medical Sciences, Tehran, Iran; Amir Taherkhani, Research Center for Molecular Medicine, Hamadan University of Medical Sciences, Hamadan, Iran; Mazyar Zahir, Urology and Nephrology Research Center, Shahid Beheshti University of Medical Sciences, Tehran, Iran; Moein Zangiabadian, Non-Communicable Diseases Research Center, Endocrinology and Metabolism Population Sciences Institute, Tehran University of Medical Sciences, Tehran, Iran; Iman Zare, Research and Development Department, Sina Medical Biochemistry Technologies, Shiraz, Iran.

## Authors note

This study is based on publicly available data and solely reflects the opinion of its authors and not that of the Institute for Health Metrics and Evaluation.

## Data availability statement

Publicly available datasets were analyzed in this study. This data can be found here: http://ghdx.healthdata.org/gbd-results-tool.

## Ethics statement

The studies involving human participants were reviewed and approved by Endocrinology and Metabolism Research Institute, Tehran University of Medical Sciences, Tehran, Iran (IR.TUMS.EMRI.REC.1400.026). The patients/participants provided their written informed consent to participate in this study.

## Author contributions

Please see supplementary material for more detailed information about individual author contributions to the research, divided into the following categories: providing data or critical feedback on data sources; developing methods or computational machinery; providing critical feedback on methods or results; drafting the manuscript or revising it critically for important intellectual content; and management of the overall research enterprise. Members of the core research team for this topic area had full access to the underlying data used to generate estimates presented in this article. All other authors had access to and reviewed estimates as part of the research evaluation process, which includes additional stages of formal review.

## Supplementary material

Supplementary Table 1 Number and age-standardized rates of deaths, disability-adjusted-life-years (DALYs), years of life lost (YLLs), and years lived with disability (YLDs) attributable to kidney dysfunction in 1990 and 2019 and the overall percentage changes from 1990-2019 in Iran by province.

Supplementary Figure 1 Trend of age-standardized rate of years of life lost (YLLs), years lived with disability (YLDs), deaths, and disability-adjusted life years (DALYs) attributable to kidney dysfunction in Iran from 1990 to 2019 by sex.

Supplementary Figure 2 Age-standardized rate of years of life lost (YLLs), years lived with disability (YLDs), deaths, and disability-adjusted life years (DALYs) attributable to kidney dysfunction among both sexes in Iran in 1990 by province.

Supplementary Figure 3 Age-standardized rate of years of life lost (YLLs), years lived with disability (YLDs), deaths, and disability-adjusted life years (DALYs) attributable to kidney dysfunction among both sexes in Iran in 2019 by province.

Supplementary Figure 4 Trend of age-standardized rate of years of life lost (YLLs), years lived with disability (YLDs), deaths, and disability-adjusted life years (DALYs) attributable to kidney dysfunction in Iran from 1990 to 2019 by province.

Supplementary Figure 5 Age-standardized rate of years of life lost (YLLs), years lived with disability (YLDs), deaths and disability-adjusted life years (DALYs) attributable to kidney dysfunction in provinces of Iran in 1990 and 2019 by sex and age.

Supplementary Figure 6 Age-standardized rate of disability-adjusted life years (DALYs) attributable to kidney dysfunction in Iran in 1990 and 2019 by socio-demographic index (SDI) quintiles and province.

Supplementary Figure 7 Age-standardized rate of deaths attributable to kidney dysfunction in Iran in 1990 and 2019 by socio-demographic index (SDI) quintiles and province.
