# Supplementary material for: Trends of national and sub-national burden attributed to kidney dysfunction risk factor in Iran: 1990-2019
Source: Front Endocrinol (Lausanne). 2023 Feb 27;14:1115833. doi: 10.3389/fendo.2023.1115833 (PMC10010168; doi:10.3389/fendo.2023.1115833)
Supplement: Supplementary Table 1 — Number and age-standardized rates of deaths, disability-adjusted-life-years (DALYs), years of life lost (YLLs), and years lived with disability (YLDs) attributable to kidney dysfunction in 1990 and 2019 and the overall percentage changes from 1990-2019 in Iran by province. [file Table_1.pdf]

Table S1. Number and age-standardized rates of deaths, disability-adjusted-life-years (DALYs), years of life lost (YLLs), and years lived with disability (YLDs) attributable to kidney dysfunction in 1990 and 2019 and overall percent change over 1990-2019 in Iran, by province.

Province	Measure	Age, Metric	Year						% Change (1990 to 2019)		
			1990			2019					
			Both	Female	Male	Both	Female	Male	Both	Female	Male
Alborz	Deaths	Attributed all ages number	280 (214 to 349)	134 (99 to 170)	146 (105 to 193)	1110 (894 to 1343)	517 (407 to 647)	593 (451 to 750)	296.2 (218.8 to 418.7)	286.5 (198.6 to 455.4)	305 (204.7 to 460.9)
		Attributed age-standardized rate (per 100,000)	69.9 (53.4 to 87.2)	72.2 (53.7 to 91.7)	67.1 (49.6 to 88.1)	60.2 (47.4 to 73.6)	67 (51.6 to 85.4)	57.3 (43.5 to 72.6)	-13.9 (-28.9 to 7.6)	-7.2 (-26.8 to 26.4)	-14.7 (-35.2 to 12.6)
	DALYs	Attributed all ages number	8207 (6488 to 9847)	3866 (2949 to 4746)	4341 (3237 to 5542)	25466 (21104 to 30428)	11438 (9350 to 13761)	14029 (11190 to 17445)	210.3 (155.8 to 303.7)	195.8 (135 to 308.3)	223.2 (148.3 to 343.9)
		Attributed age-standardized rate (per 100,000)	1376 (1072.1 to 1682)	1386.1 (1063.6 to 1731.1)	1354 (1004.1 to 1758.2)	1109.9 (914.2 to 1321.5)	1109.7 (899.9 to 1346.6)	1138.5 (904.1 to 1411.4)	-19.3 (-33 to 1.5)	-19.9 (-36.2 to 9.2)	-15.9 (-35.4 to 12.7)
	YLLs	Attributed all ages number	7019 (5268 to 8701)	3187 (2280 to 4056)	3832 (2720 to 5033)	21050 (17129 to 25297)	9110 (7241 to 11288)	11940 (9172 to 15099)	199.9 (138.7 to 310.1)	185.8 (115.2 to 321.2)	211.6 (128.9 to 348.3)
		Attributed age-standardized rate (per 100,000)	1227.4 (933 to 1526.5)	1213.3 (906.2 to 1540.1)	1228.3 (891.7 to 1621.2)	941.9 (766 to 1131.4)	930.1 (736.4 to 1157.9)	981.4 (749.1 to 1239.4)	-23.3 (-37.8 to 0.6)	-23.3 (-40.8 to 11)	-20.1 (-40.2 to 10.5)
	YLDs	Attributed all ages number	1188 (858 to 1555)	679 (489 to 906)	509 (359 to 670)	4416 (3203 to 5748)	2328 (1667 to 3044)	2089 (1520 to 2786)	271.8 (229.5 to 314.6)	242.8 (198.7 to 289.7)	310.6 (258.4 to 372.3)
		Attributed age-standardized rate (per 100,000)	148.6 (109.6 to 194.1)	172.8 (127 to 226.9)	125.7 (91.8 to 166.1)	168 (122.9 to 222.6)	179.6 (129.9 to 235.6)	157.2 (114.4 to 209.7)	13 (4.8 to 21.5)	4 (-6.8 to 14.3)	25 (12.7 to 39.1)
Ardebil	Deaths	Attributed all ages number	300 (239 to 361)	133 (103 to 162)	167 (128 to 211)	762 (629 to 899)	346 (282 to 413)	416 (336 to 496)	153.8 (114.2 to 208.5)	160.9 (112.4 to 225.3)	148.2 (92.9 to 218)
		Attributed age-standardized rate (per 100,000)	78.7 (63.1 to 97.4)	75.1 (57.6 to 93.6)	81.7 (62.7 to 103.5)	76.9 (62.9 to 91.2)	67.9 (54.8 to 81.2)	86.7 (69.6 to 103.6)	-2.2 (-16.6 to 15.8)	-9.7 (-25 to 13.3)	6 (-15.6 to 32.6)
	DALYs	Attributed all ages number	9850 (7963 to 11634)	4532 (3547 to 5555)	5317 (4000 to 6722)	15840 (13580 to 18237)	7023 (5964 to 8148)	8817 (7362 to 10336)	60.8 (34.5 to 98.2)	55 (23.8 to 97.5)	65.8 (28 to 115.4)

Province	Measure	Age, Metric	Year						% Change (1990 to 2019)		
			1990			2019					
			Both	Female	Male	Both	Female	Male	Both	Female	Male
		Attributed age-standardized rate (per 100,000)	1684.3 (1371.7 to 2011.6)	1625.4 (1306.9 to 1971.3)	1729.9 (1359.4 to 2165.2)	1440.6 (1235.8 to 1665.6)	1257.7 (1063.2 to 1461.3)	1634.5 (1353.2 to 1933.7)	-14.5 (-26.6 to 1.3)	-22.6 (-35.5 to -5.6)	-5.5 (-24.6 to 19.1)
	YLLs	Attributed all ages number	8863 (6980 to 10549)	3977 (3011 to 4968)	4886 (3587 to 6234)	13683 (11639 to 15803)	5860 (4884 to 6861)	7823 (6459 to 9292)	54.4 (26.6 to 92.5)	47.3 (13.9 to 94.6)	60.1 (20.8 to 112.5)
		Attributed age-standardized rate (per 100,000)	1533.5 (1225 to 1842.8)	1450.3 (1128.1 to 1773.7)	1602.4 (1230.1 to 2019.6)	1259.2 (1065.9 to 1466)	1064.2 (884.1 to 1249.1)	1464.7 (1201.7 to 1747.9)	-17.9 (-30.6 to -1.1)	-26.6 (-40.1 to -8.2)	-8.6 (-28.7 to 17.1)
	YLDs	Attributed all ages number	986 (706 to 1302)	555 (398 to 735)	431 (304 to 569)	2157 (1566 to 2824)	1163 (842 to 1529)	994 (720 to 1318)	118.7 (95.8 to 143.9)	109.4 (85.1 to 140)	130.6 (99.7 to 162.4)
		Attributed age-standardized rate (per 100,000)	150.7 (111.7 to 196.3)	175.2 (128.6 to 227.4)	127.5 (93.5 to 170.4)	181.4 (133.3 to 239.2)	193.4 (141.2 to 255.1)	169.8 (122.4 to 225.8)	20.3 (11.3 to 29.9)	10.4 (0.2 to 22.2)	33.2 (19.5 to 47.5)
Bushehr	Deaths	Attributed all ages number	158 (126 to 195)	70 (55 to 87)	88 (65 to 114)	417 (347 to 493)	187 (154 to 224)	230 (188 to 276)	163.3 (120.4 to 225.3)	165.7 (115.4 to 235.4)	161.4 (103.4 to 248.3)
		Attributed age-standardized rate (per 100,000)	79.5 (62.6 to 98.6)	73.1 (56.1 to 91.2)	85.7 (65.8 to 109.7)	65.7 (53 to 79)	59.5 (47.4 to 72.4)	72.1 (58.1 to 87.7)	-17.3 (-29.9 to -1.7)	-18.6 (-32.9 to -1.2)	-15.9 (-32.3 to 6.7)
	DALYs	Attributed all ages number	4752 (3891 to 5720)	2152 (1749 to 2611)	2599 (2013 to 3304)	9930 (8535 to 11475)	4423 (3755 to 5132)	5508 (4571 to 6480)	109 (78 to 155.7)	105.5 (71 to 153.5)	111.9 (68.3 to 174.4)
		Attributed age-standardized rate (per 100,000)	1626.3 (1321.7 to 1984.2)	1500.3 (1189.1 to 1832.3)	1743.4 (1330.3 to 2210.3)	1243.6 (1053.8 to 1452.1)	1119.6 (939 to 1313)	1370.5 (1135.6 to 1621.2)	-23.5 (-34.8 to -8.7)	-25.4 (-37.6 to -8.9)	-21.4 (-37.2 to 0.4)
	YLLs	Attributed all ages number	4168 (3352 to 5100)	1821 (1426 to 2249)	2347 (1764 to 3044)	8249 (7023 to 9643)	3527 (2972 to 4178)	4722 (3897 to 5621)	97.9 (63.5 to 151.4)	93.6 (56.3 to 146.5)	101.2 (55.4 to 169.6)
		Attributed age-standardized rate (per 100,000)	1472.5 (1166.4 to 1819.6)	1327.1 (1035.1 to 1648.1)	1609 (1207.1 to 2071.3)	1068.5 (895.8 to 1256.1)	930.9 (769.4 to 1106.8)	1207.8 (993.5 to 1452.3)	-27.4 (-39.1 to -11.4)	-29.9 (-42.9 to -11)	-24.9 (-41 to -2.3)
	YLDs	Attributed all ages number	583 (423 to 769)	331 (239 to 439)	252 (177 to 335)	1681 (1210 to 2187)	896 (644 to 1169)	786 (558 to 1041)	188.2 (157.7 to 219.1)	170.6 (139.1 to 206.4)	211.4 (172.5 to 256.7)

Province	Measure	Age, Metric	Year						% Change (1990 to 2019)		
			1990			2019					
			Both	Female	Male	Both	Female	Male	Both	Female	Male
		Attributed age-standardized rate (per 100,000)	153.8 (113.8 to 199.9)	173.2 (127.7 to 223.7)	134.4 (97.9 to 177)	175.1 (127.9 to 229.1)	188.6 (138.2 to 248.9)	162.8 (117.7 to 216.9)	13.8 (4.8 to 22.6)	8.9 (-1.7 to 20)	21.1 (9.6 to 34.6)
Chahar Mahaal and Bakhtiari	Deaths	Attributed all ages number	141 (111 to 174)	61 (46 to 80)	80 (60 to 103)	352 (272 to 443)	148 (108 to 197)	204 (155 to 257)	149.1 (102.7 to 209.4)	141.8 (80.3 to 230)	154.6 (95.9 to 238.1)
		Attributed age-standardized rate (per 100,000)	64.5 (49.3 to 81)	59.2 (43.2 to 78)	69.5 (51.6 to 89.3)	50.1 (38.5 to 63.7)	41.1 (29.5 to 54.8)	59.8 (45.5 to 75.8)	-22.3 (-35.1 to -6.3)	-30.6 (-47.2 to -6.7)	-13.9 (-32.4 to 8.8)
	DALYs	Attributed all ages number	4020 (3307 to 4814)	1768 (1403 to 2211)	2252 (1760 to 2835)	7322 (5929 to 8755)	3069 (2460 to 3834)	4253 (3435 to 5167)	82.1 (52.6 to 120.6)	73.6 (36.2 to 125.5)	88.9 (47.8 to 141.1)
		Attributed age-standardized rate (per 100,000)	1281.7 (1025.4 to 1558.4)	1179.5 (909.4 to 1504)	1367.9 (1050.6 to 1731)	932 (752.1 to 1129.7)	765.4 (610.8 to 966.7)	1108.7 (880.9 to 1358.9)	-27.3 (-39.2 to -12.1)	-35.1 (-49.2 to -15.1)	-19 (-36.7 to 2.1)
	YLLs	Attributed all ages number	3497 (2819 to 4225)	1476 (1133 to 1895)	2020 (1550 to 2590)	6010 (4806 to 7395)	2360 (1797 to 3101)	3649 (2883 to 4524)	71.9 (40 to 116)	59.9 (18 to 122.3)	80.6 (37.1 to 136.4)
		Attributed age-standardized rate (per 100,000)	1153.3 (908.8 to 1421.1)	1033.6 (771.2 to 1346.6)	1257 (949.8 to 1608.5)	782.1 (620.3 to 965.8)	605.9 (458.1 to 805.4)	968.1 (754.5 to 1207.2)	-32.2 (-44.4 to -16)	-41.4 (-56.4 to -19.9)	-23 (-41.3 to -0.4)
	YLDs	Attributed all ages number	523 (382 to 684)	292 (213 to 382)	231 (167 to 304)	1312 (958 to 1707)	708 (516 to 922)	604 (433 to 793)	150.9 (124.9 to 182)	143 (111.8 to 178.1)	160.8 (127 to 199.7)
		Attributed age-standardized rate (per 100,000)	128.3 (95.7 to 166.9)	145.8 (107.4 to 188.4)	110.9 (81.6 to 146.1)	149.9 (109.3 to 195.8)	159.5 (115.5 to 208.2)	140.6 (101.7 to 185.8)	16.8 (7.8 to 26.4)	9.4 (-1 to 21.5)	26.8 (15 to 40.7)
East Azarbayejan	Deaths	Attributed all ages number	1059 (852 to 1297)	472 (361 to 598)	587 (439 to 755)	2291 (1885 to 2769)	1117 (894 to 1387)	1174 (933 to 1456)	116.4 (75.7 to 162.5)	136.6 (80.2 to 211.5)	100.2 (53.3 to 170.9)
		Attributed age-standardized rate (per 100,000)	94.6 (75.6 to 117.1)	91.9 (70.2 to 116)	96.5 (73.2 to 122.2)	76.8 (62.5 to 93.5)	76.3 (60.7 to 94.9)	77.3 (62 to 96.6)	-18.8 (-32.9 to -3.3)	-17 (-36.2 to 6.9)	-19.9 (-37.9 to 4.7)
	DALYs	Attributed all ages number	31713 (26312 to 38126)	14397 (11464 to 18052)	17316 (13265 to 22027)	49430 (42195 to 57732)	23292 (19213 to 27957)	26139 (21202 to 31894)	55.9 (29.6 to 87.2)	61.8 (25.6 to 109.2)	50.9 (16.9 to 98.7)

Province	Measure	Age, Metric	Year						% Change (1990 to 2019)		
			1990			2019					
			Both	Female	Male	Both	Female	Male	Both	Female	Male
		Attributed age-standardized rate (per 100,000)	1856.1 (1510.4 to 2242.7)	1789 (1402.8 to 2213)	1907.6 (1465.7 to 2402)	1384.5 (1173.5 to 1621.3)	1327.5 (1092 to 1603)	1442.1 (1178.5 to 1764.2)	-25.4 (-37.4 to -11.4)	-25.8 (-42.2 to -5.7)	-24.4 (-41.2 to -1.8)
	YLLs	Attributed all ages number	28417 (23041 to 34550)	12550 (9751 to 16081)	15867 (11785 to 20535)	42223 (35297 to 50500)	19468 (15756 to 23917)	22754 (18038 to 28345)	48.6 (19.4 to 83.5)	55.1 (16.4 to 108.5)	43.4 (7.1 to 95.1)
		Attributed age-standardized rate (per 100,000)	1695 (1359.8 to 2064.8)	1603.9 (1228.3 to 2032)	1769.6 (1339.2 to 2259.5)	1199.7 (997 to 1437.2)	1131.6 (911.4 to 1389.1)	1267.9 (1012.6 to 1575.8)	-29.2 (-42 to -14.9)	-29.4 (-46.2 to -7.6)	-28.4 (-45.6 to -4.6)
	YLDs	Attributed all ages number	3296 (2392 to 4292)	1847 (1350 to 2425)	1449 (1038 to 1912)	7208 (5245 to 9482)	3823 (2759 to 5016)	3384 (2436 to 4455)	118.7 (97.9 to 142)	107 (84 to 133)	133.6 (105.3 to 165.5)
		Attributed age-standardized rate (per 100,000)	161.1 (118.6 to 208.5)	185.1 (136 to 238.7)	138.1 (100.7 to 181.3)	184.8 (136.2 to 241.7)	195.9 (142.8 to 256)	174.2 (126.8 to 230)	14.7 (6.4 to 24.1)	5.8 (-4.5 to 17.3)	26.2 (13.4 to 40.1)
Fars	Deaths	Attributed all ages number	1033 (810 to 1283)	457 (343 to 577)	576 (416 to 752)	2339 (1871 to 2851)	1111 (858 to 1381)	1228 (953 to 1568)	126.5 (83.3 to 185)	143.1 (88.8 to 221.7)	113.4 (55.8 to 197.8)
		Attributed age-standardized rate (per 100,000)	91.6 (71.2 to 114)	85.4 (63.7 to 108.9)	96.8 (70.2 to 125.6)	65.2 (51.8 to 80)	65.3 (49.7 to 82.1)	65.5 (50.1 to 83.5)	-28.8 (-40.9 to -12.6)	-23.5 (-39.2 to -1.2)	-32.4 (-48.6 to -7.5)
	DALYs	Attributed all ages number	29609 (24149 to 35969)	12817 (10112 to 15850)	16791 (12862 to 21508)	52145 (43763 to 61820)	23951 (19511 to 28421)	28193 (22382 to 35313)	76.1 (45.6 to 118)	86.9 (49.3 to 141.9)	67.9 (26.2 to 125.7)
		Attributed age-standardized rate (per 100,000)	1808.1 (1447.8 to 2202.7)	1637.5 (1275 to 2043.4)	1957.4 (1468.5 to 2528.4)	1234.8 (1035.9 to 1462.7)	1174.9 (949.5 to 1408.1)	1297.3 (1033.2 to 1615.2)	-31.7 (-42.8 to -15.7)	-28.2 (-42.3 to -7.8)	-33.7 (-49.7 to -9.1)
	YLLs	Attributed all ages number	25976 (20660 to 32152)	10857 (8326 to 13631)	15119 (11247 to 19679)	43699 (35843 to 52367)	19497 (15311 to 24006)	24202 (18698 to 30835)	68.2 (34.9 to 115.8)	79.6 (36.6 to 144.4)	60.1 (16.8 to 124.5)
		Attributed age-standardized rate (per 100,000)	1641.6 (1293.4 to 2027.1)	1456.6 (1093.7 to 1835.3)	1805.1 (1306.2 to 2353.3)	1054.2 (858.2 to 1268.9)	984.5 (765.5 to 1208.4)	1126.3 (873.4 to 1432.3)	-35.8 (-47.8 to -19.2)	-32.4 (-47.4 to -9.4)	-37.6 (-54 to -12)
	YLDs	Attributed all ages number	3632 (2574 to 4794)	1961 (1392 to 2587)	1672 (1173 to 2267)	8445 (6189 to 11134)	4454 (3190 to 5875)	3991 (2871 to 5297)	132.5 (107.7 to 160.6)	127.2 (98.3 to 160.4)	138.7 (107.3 to 176.3)

Province	Measure	Age, Metric	Year						% Change (1990 to 2019)		
			1990			2019					
			Both	Female	Male	Both	Female	Male	Both	Female	Male
		Attributed age-standardized rate (per 100,000)	166.5 (121.9 to 217.7)	181 (133.6 to 237.1)	152.3 (111.1 to 202.9)	180.6 (132.9 to 235.8)	190.5 (138.2 to 248.9)	171 (124.2 to 226.8)	8.4 (-0.1 to 17.6)	5.2 (-5 to 16.8)	12.3 (0.4 to 25.2)
Gilan	Deaths	Attributed all ages number	758 (601 to 931)	382 (297 to 483)	376 (283 to 487)	1815 (1474 to 2204)	933 (732 to 1148)	882 (698 to 1106)	139.5 (97.7 to 197.1)	144.2 (91.1 to 218)	134.6 (72.5 to 219.5)
		Attributed age-standardized rate (per 100,000)	87.4 (69.3 to 108.2)	85.7 (66.2 to 108.9)	87.5 (66 to 112.7)	66.9 (53.8 to 81.5)	70.4 (54.9 to 87.3)	63.9 (50.3 to 80.4)	-23.5 (-35.1 to -8.2)	-17.9 (-34.2 to 4.8)	-27 (-43.6 to -4.7)
	DALYs	Attributed all ages number	20499 (16450 to 24811)	9877 (7907 to 12381)	10622 (8102 to 13475)	36668 (30689 to 43627)	17941 (14580 to 21541)	18727 (15094 to 22912)	78.9 (50.2 to 119.3)	81.7 (43.7 to 134.9)	76.3 (33.7 to 135.1)
		Attributed age-standardized rate (per 100,000)	1706.6 (1374.3 to 2070.8)	1647.4 (1310.1 to 2043.8)	1745.7 (1330.4 to 2229.9)	1252.3 (1049.7 to 1487.8)	1240.1 (1001.3 to 1494)	1268.7 (1028.6 to 1549.5)	-26.6 (-37.6 to -11.6)	-24.7 (-39.4 to -4.5)	-27.3 (-43.9 to -4.6)
	YLLs	Attributed all ages number	18278 (14454 to 22344)	8595 (6677 to 10981)	9683 (7259 to 12403)	31640 (26200 to 37846)	15281 (12112 to 18478)	16358 (13056 to 20307)	73.1 (42 to 118.2)	77.8 (36.5 to 141.7)	68.9 (23.4 to 134.5)
		Attributed age-standardized rate (per 100,000)	1556.2 (1234.7 to 1909.2)	1477.1 (1145.9 to 1855.2)	1617.7 (1222.9 to 2083.2)	1081.4 (889.1 to 1295)	1059.4 (840.6 to 1280.6)	1107.5 (880.2 to 1381.5)	-30.5 (-42 to -14.4)	-28.3 (-43.9 to -5.7)	-31.5 (-48.7 to -8.1)
	YLDs	Attributed all ages number	2222 (1620 to 2909)	1282 (930 to 1684)	940 (663 to 1238)	5029 (3668 to 6594)	2660 (1940 to 3482)	2368 (1708 to 3153)	126.4 (104.2 to 151.7)	107.5 (84.5 to 133.4)	152 (119.9 to 193.9)
		Attributed age-standardized rate (per 100,000)	150.4 (110.9 to 196.3)	170.2 (125.5 to 221.9)	128 (93.3 to 167.1)	170.9 (124.8 to 223.7)	180.7 (132.3 to 236.3)	161.2 (117.5 to 216.1)	13.6 (4.9 to 23.2)	6.2 (-3.9 to 16.8)	25.9 (13 to 40)
Golestan	Deaths	Attributed all ages number	408 (331 to 492)	185 (143 to 230)	223 (171 to 284)	959 (797 to 1120)	447 (362 to 540)	512 (409 to 616)	134.7 (98.5 to 184.1)	141 (92.1 to 207)	129.4 (78.4 to 207)
		Attributed age-standardized rate (per 100,000)	99.3 (78.9 to 120.6)	93.3 (71.9 to 116)	105.3 (80.6 to 134)	79.5 (65 to 94.2)	74.1 (59.3 to 90.6)	85.6 (68.1 to 103.5)	-19.9 (-30.7 to -6)	-20.6 (-35 to -0.1)	-18.7 (-34.7 to 4.4)
	DALYs	Attributed all ages number	12366 (10284 to 14611)	5648 (4543 to 6897)	6718 (5289 to 8425)	23401 (20000 to 26715)	10880 (9150 to 12862)	12521 (10378 to 14921)	89.2 (62.5 to 127.4)	92.7 (54.8 to 139)	86.4 (48.6 to 139.7)

Province	Measure	Age, Metric	Year						% Change (1990 to 2019)		
			1990			2019					
			Both	Female	Male	Both	Female	Male	Both	Female	Male
		Attributed age-standardized rate (per 100,000)	2093.6 (1727.6 to 2504.1)	1972.6 (1553.3 to 2405.1)	2201.8 (1714.5 to 2779.2)	1619.1 (1373.2 to 1862.2)	1485.7 (1232.8 to 1759.7)	1762.3 (1448.4 to 2108.9)	-22.7 (-33.1 to -8.7)	-24.7 (-38.6 to -6.9)	-20 (-35.6 to 3.2)
	YLLs	Attributed all ages number	10951 (8932 to 13094)	4857 (3751 to 5990)	6094 (4740 to 7746)	20339 (17183 to 23380)	9257 (7651 to 11013)	11082 (8982 to 13218)	85.7 (56 to 127.2)	90.6 (49 to 144.2)	81.9 (40.8 to 140.9)
		Attributed age-standardized rate (per 100,000)	1904.8 (1550.8 to 2293.2)	1759.4 (1353.2 to 2173)	2038.1 (1556.9 to 2595.4)	1429.8 (1202.2 to 1656.9)	1288.5 (1056 to 1536.4)	1580.8 (1264.3 to 1902.3)	-24.9 (-36.3 to -9.8)	-26.8 (-41.7 to -6.6)	-22.4 (-38.9 to 2.6)
	YLDs	Attributed all ages number	1414 (1016 to 1830)	790 (559 to 1036)	624 (442 to 825)	3062 (2217 to 3990)	1623 (1176 to 2139)	1439 (1022 to 1879)	116.5 (94.2 to 141.4)	105.4 (78.4 to 135.4)	130.5 (102.6 to 161.1)
		Attributed age-standardized rate (per 100,000)	188.8 (137.9 to 245.7)	213.2 (154.9 to 279.6)	163.7 (118.6 to 214.2)	189.3 (139.6 to 246.3)	197.1 (144.8 to 260.2)	181.6 (132 to 240.4)	0.3 (-8.6 to 9.3)	-7.6 (-17.6 to 3.7)	10.9 (0.2 to 23.6)
Hamadan	Deaths	Attributed all ages number	497 (395 to 603)	223 (174 to 287)	274 (206 to 355)	1087 (869 to 1327)	524 (415 to 652)	563 (435 to 706)	118.6 (79.2 to 166.6)	134.6 (85 to 200.6)	105.4 (52.3 to 177.9)
		Attributed age-standardized rate (per 100,000)	81.7 (63.5 to 100.4)	81.8 (62.4 to 106.1)	80.3 (60.4 to 104.6)	65.6 (52.2 to 80.2)	66.8 (52.8 to 83.5)	65.9 (51 to 82.6)	-19.8 (-32.8 to -4.4)	-18.4 (-34.4 to 2.5)	-18 (-38 to 7.6)
	DALYs	Attributed all ages number	14615 (11996 to 17243)	6566 (5346 to 8099)	8049 (6250 to 10064)	22157 (18620 to 26053)	10196 (8535 to 12194)	11962 (9616 to 14601)	51.6 (26.4 to 81.4)	55.3 (24.8 to 93.5)	48.6 (14.4 to 95.6)
		Attributed age-standardized rate (per 100,000)	1680.4 (1358.5 to 2012.7)	1617.5 (1289.3 to 2025.5)	1724.6 (1330.9 to 2200.2)	1282.7 (1075.8 to 1519.6)	1205.3 (1006.8 to 1444.2)	1372.2 (1103.2 to 1678.3)	-23.7 (-35.6 to -9)	-25.5 (-39.3 to -7.4)	-20.4 (-38.8 to 4.9)
	YLLs	Attributed all ages number	13135 (10580 to 15663)	5736 (4538 to 7198)	7399 (5642 to 9427)	19145 (15960 to 23032)	8598 (6966 to 10546)	10547 (8354 to 13107)	45.8 (18.4 to 78.3)	49.9 (16.4 to 93.3)	42.5 (7 to 92.8)
		Attributed age-standardized rate (per 100,000)	1536.1 (1225.1 to 1858.8)	1451.1 (1129.5 to 1861.6)	1601.5 (1220.4 to 2066)	1112.4 (922.9 to 1335.5)	1024.1 (828.7 to 1258.9)	1212.3 (955.5 to 1506.2)	-27.6 (-40 to -11.9)	-29.4 (-44.1 to -9.6)	-24.3 (-43.4 to 2.4)
	YLDs	Attributed all ages number	1480 (1080 to 1962)	830 (608 to 1099)	650 (460 to 864)	3012 (2224 to 3945)	1598 (1163 to 2108)	1414 (1018 to 1879)	103.6 (83.2 to 128)	92.5 (70.6 to 119.2)	117.7 (90.3 to 149.5)

Province	Measure	Age, Metric	Year						% Change (1990 to 2019)		
			1990			2019					
			Both	Female	Male	Both	Female	Male	Both	Female	Male
		Attributed age-standardized rate (per 100,000)	144.4 (107.1 to 188.6)	166.5 (122.7 to 220)	123 (89.4 to 160.5)	170.3 (125.4 to 223.9)	181.2 (132.8 to 238.2)	159.9 (115 to 212.7)	18 (8.8 to 28.1)	8.8 (-1.6 to 21.4)	29.9 (17.4 to 44.5)
Hormozgan	Deaths	Attributed all ages number	292 (215 to 355)	127 (95 to 162)	165 (112 to 211)	648 (541 to 766)	304 (250 to 362)	343 (276 to 425)	121.5 (81.6 to 204.2)	139.3 (88.5 to 214.4)	107.9 (56.1 to 201)
		Attributed age-standardized rate (per 100,000)	92.6 (71.3 to 113.1)	84.3 (64 to 109.1)	100.9 (73.5 to 127.3)	66.9 (55.4 to 79.4)	67.8 (55 to 81.4)	67.6 (54.2 to 83.9)	-27.8 (-40 to -9.4)	-19.6 (-36.9 to 2.6)	-32.9 (-48.1 to -8.4)
	DALYs	Attributed all ages number	8730 (6275 to 10444)	3875 (2725 to 4810)	4855 (3313 to 6072)	15903 (13740 to 18310)	7188 (6095 to 8356)	8715 (7215 to 10487)	82.2 (51.6 to 157.6)	85.5 (48.7 to 163.6)	79.5 (37.6 to 160.2)
		Attributed age-standardized rate (per 100,000)	1932.2 (1434 to 2320.7)	1777.7 (1331 to 2215.1)	2065.6 (1455.3 to 2595)	1350.8 (1157 to 1566.2)	1275 (1073 to 1486.4)	1441.3 (1184.3 to 1750.2)	-30.1 (-41.6 to -7.3)	-28.3 (-41.8 to -8.1)	-30.2 (-46.2 to -2.7)
	YLLs	Attributed all ages number	7886 (5447 to 9530)	3387 (2243 to 4287)	4498 (2967 to 5719)	13231 (11353 to 15369)	5732 (4790 to 6751)	7499 (6088 to 9146)	67.8 (36.7 to 149.3)	69.2 (30.9 to 155.7)	66.7 (25 to 152.4)
		Attributed age-standardized rate (per 100,000)	1776.3 (1290.2 to 2152.9)	1595.3 (1156.2 to 2019.1)	1935.7 (1333 to 2461.6)	1161.5 (988.2 to 1366.2)	1070.3 (885.6 to 1265)	1267 (1025.1 to 1558.9)	-34.6 (-46.2 to -10.7)	-32.9 (-47.1 to -10.5)	-34.5 (-50.6 to -5.8)
	YLDs	Attributed all ages number	844 (617 to 1099)	487 (352 to 629)	356 (256 to 471)	2671 (1954 to 3467)	1455 (1051 to 1904)	1216 (860 to 1585)	216.5 (184.8 to 249.4)	198.5 (161.5 to 237.9)	241.2 (199.1 to 290.6)
		Attributed age-standardized rate (per 100,000)	155.8 (116.2 to 201.7)	182.4 (135.5 to 235.8)	129.8 (94.2 to 169.4)	189.2 (140.3 to 243.8)	204.8 (150.2 to 265.3)	174.4 (126.5 to 229.5)	21.4 (12.3 to 31.3)	12.2 (0.5 to 24.5)	34.3 (20.5 to 49.9)
Ilam	Deaths	Attributed all ages number	128 (103 to 155)	49 (39 to 60)	79 (61 to 98)	321 (274 to 370)	135 (112 to 160)	186 (152 to 221)	151.3 (115.5 to 202.8)	174.3 (123.2 to 248.7)	137 (89.5 to 200.6)
		Attributed age-standardized rate (per 100,000)	96.6 (76.3 to 118.2)	85.9 (65.9 to 108.1)	104.7 (80.5 to 132.2)	89.1 (74.4 to 103.8)	81 (65.4 to 97.5)	95.2 (77.2 to 113.2)	-7.8 (-20.4 to 8.9)	-5.7 (-23.2 to 18.7)	-9.1 (-26.3 to 13.8)
	DALYs	Attributed all ages number	3850 (3162 to 4551)	1540 (1222 to 1846)	2310 (1802 to 2854)	7323 (6442 to 8246)	3189 (2708 to 3722)	4134 (3471 to 4817)	90.2 (62.9 to 131.1)	107 (67.1 to 160.3)	79 (42.7 to 131)

Province	Measure	Age, Metric	Year						% Change (1990 to 2019)		
			1990			2019					
			Both	Female	Male	Both	Female	Male	Both	Female	Male
		Attributed age-standardized rate (per 100,000)	1982.2 (1599 to 2374.8)	1744.4 (1387.6 to 2130.1)	2156.5 (1712.7 to 2683)	1652.1 (1444 to 1875)	1502.8 (1264 to 1751.9)	1780.5 (1487.5 to 2089.5)	-16.7 (-27.7 to -0.3)	-13.8 (-28.8 to 7.6)	-17.4 (-33.3 to 4.1)
	YLLs	Attributed all ages number	3474 (2800 to 4131)	1343 (1042 to 1633)	2131 (1643 to 2674)	6349 (5517 to 7222)	2690 (2262 to 3171)	3659 (3036 to 4308)	82.8 (54.3 to 127.6)	100.2 (57.4 to 160.5)	71.7 (34 to 127.7)
		Attributed age-standardized rate (per 100,000)	1825.3 (1461.6 to 2200.5)	1571.4 (1235.6 to 1938)	2014.6 (1573.9 to 2544.2)	1460.8 (1258.8 to 1668.3)	1305.3 (1087.1 to 1540.7)	1594.7 (1313.7 to 1886)	-20 (-31.4 to -2.8)	-16.9 (-33.1 to 7)	-20.8 (-36.9 to 2)
	YLDs	Attributed all ages number	376 (274 to 497)	197 (142 to 258)	179 (128 to 240)	974 (701 to 1284)	499 (361 to 658)	475 (343 to 631)	159.2 (131.8 to 192)	153.6 (118.5 to 193.1)	165.4 (129.1 to 206.8)
		Attributed age-standardized rate (per 100,000)	156.9 (116.4 to 206.2)	173 (127.2 to 226.7)	141.8 (103.7 to 189.1)	191.3 (140.4 to 251.3)	197.5 (143.6 to 260.5)	185.8 (133.9 to 244.3)	21.9 (12.6 to 31.7)	14.2 (2.3 to 26.6)	31 (18.2 to 44.6)
Isfahan	Deaths	Attributed all ages number	1051 (801 to 1336)	480 (360 to 619)	570 (398 to 779)	2664 (2080 to 3275)	1232 (957 to 1532)	1432 (1080 to 1837)	153.5 (101.3 to 227.6)	156.4 (98 to 240.2)	151.1 (82.5 to 256.9)
		Attributed age-standardized rate (per 100,000)	81.3 (61 to 102.7)	76.9 (57.1 to 99.2)	84.3 (60.6 to 114.7)	60.6 (47 to 74.8)	60.4 (46.2 to 75.8)	61.4 (46.3 to 78.8)	-25.5 (-39 to -7.6)	-21.4 (-38.2 to 1.8)	-27.1 (-45 to -1.5)
	DALYs	Attributed all ages number	28697 (22443 to 35726)	12436 (9582 to 15574)	16260 (11679 to 21781)	54828 (44864 to 65928)	23972 (19427 to 28830)	30856 (23956 to 38741)	91.1 (54.2 to 140.7)	92.8 (51.7 to 151.5)	89.8 (41.8 to 162.4)
		Attributed age-standardized rate (per 100,000)	1568.7 (1229.7 to 1952.9)	1415.4 (1087.2 to 1796.1)	1699.4 (1222.3 to 2275.8)	1115.7 (912.3 to 1338.6)	1028.9 (830.6 to 1243.3)	1206 (938 to 1517.3)	-28.9 (-41.7 to -11.1)	-27.3 (-42.2 to -6.7)	-29 (-46.7 to -3.7)
	YLLs	Attributed all ages number	25301 (19396 to 31975)	10602 (7967 to 13648)	14699 (10316 to 20021)	46449 (36931 to 56520)	19776 (15422 to 24189)	26674 (20238 to 34118)	83.6 (45.5 to 141.6)	86.5 (40.4 to 158.3)	81.5 (30.9 to 160.2)
		Attributed age-standardized rate (per 100,000)	1417.9 (1077 to 1806.3)	1252.5 (937.2 to 1612.2)	1561.2 (1097.9 to 2134.1)	955 (758.1 to 1158.7)	865.9 (675 to 1060)	1047.5 (797 to 1336.8)	-32.6 (-46.3 to -13.3)	-30.9 (-46.9 to -7.9)	-32.9 (-51.1 to -5.1)
	YLDs	Attributed all ages number	3396 (2453 to 4492)	1835 (1326 to 2418)	1561 (1112 to 2081)	8379 (6070 to 11171)	4196 (3021 to 5582)	4183 (2993 to 5597)	146.7 (122.9 to 176.3)	128.7 (102 to 161.2)	167.9 (134.5 to 206.7)

Province	Measure	Age, Metric	Year						% Change (1990 to 2019)		
			1990			2019					
			Both	Female	Male	Both	Female	Male	Both	Female	Male
		Attributed age-standardized rate (per 100,000)	150.8 (111.2 to 198.8)	162.9 (119.9 to 214.2)	138.1 (101.5 to 183.6)	160.7 (117.7 to 213.3)	163 (118 to 216.9)	158.5 (113.5 to 211.7)	6.6 (-2 to 15.5)	0.1 (-10.2 to 11.7)	14.8 (2.7 to 27.8)
Kerman	Deaths	Attributed all ages number	618 (494 to 745)	264 (203 to 334)	354 (271 to 445)	1312 (1074 to 1562)	624 (497 to 759)	688 (554 to 842)	112.1 (75.6 to 158.9)	136.2 (88.1 to 201.4)	94.1 (47.7 to 157.8)
		Attributed age-standardized rate (per 100,000)	97.6 (77.1 to 119.8)	88.9 (68.3 to 111.9)	105.5 (81.3 to 131.3)	67.2 (54.6 to 80.5)	67 (52.8 to 82.6)	67.6 (53.9 to 82)	-31.1 (-41.7 to -18.4)	-24.6 (-39 to -5.6)	-36 (-49.4 to -18.5)
	DALYs	Attributed all ages number	18262 (14926 to 21758)	7861 (6232 to 9634)	10401 (8094 to 12976)	30025 (25553 to 34697)	13764 (11571 to 16382)	16261 (13339 to 19620)	64.4 (37.6 to 98.2)	75.1 (41.1 to 119.5)	56.3 (20.8 to 104.5)
		Attributed age-standardized rate (per 100,000)	2001.5 (1618.9 to 2387.7)	1791.4 (1403.2 to 2216.9)	2186.9 (1706 to 2701.3)	1247.7 (1051.2 to 1453.4)	1187.7 (990.3 to 1426.6)	1309.1 (1069.6 to 1574.8)	-37.7 (-47.4 to -25.7)	-33.7 (-45.9 to -17.5)	-40.1 (-53.3 to -22.2)
	YLLs	Attributed all ages number	16531 (13267 to 19982)	6897 (5323 to 8701)	9634 (7383 to 12180)	25199 (21076 to 29622)	11216 (9189 to 13535)	13983 (11321 to 17060)	52.4 (23.7 to 87.1)	62.6 (26.6 to 111)	45.1 (10 to 95.8)
		Attributed age-standardized rate (per 100,000)	1843.3 (1465.6 to 2226.5)	1613.5 (1242 to 2038.1)	2048.7 (1576.2 to 2556.3)	1073.9 (891.7 to 1269)	1003.5 (816.9 to 1215)	1144.9 (924.5 to 1398)	-41.7 (-51.6 to -29.4)	-37.8 (-50.2 to -20.3)	-44.1 (-57 to -25.7)
	YLDs	Attributed all ages number	1731 (1260 to 2287)	964 (706 to 1283)	767 (545 to 1019)	4826 (3498 to 6257)	2549 (1837 to 3297)	2277 (1629 to 2976)	178.7 (152.2 to 207.7)	164.3 (134.6 to 198.7)	196.9 (161.1 to 238.7)
		Attributed age-standardized rate (per 100,000)	158.2 (116.7 to 207.4)	177.9 (132.5 to 233.8)	138.2 (101.5 to 184.1)	173.8 (127.9 to 226.2)	184.2 (135 to 239.8)	164.2 (117.9 to 215.5)	9.8 (2.2 to 18.4)	3.6 (-5.6 to 13.9)	18.8 (7.5 to 31.1)
Kermanshah	Deaths	Attributed all ages number	528 (417 to 644)	209 (161 to 259)	318 (238 to 412)	1082 (889 to 1319)	522 (413 to 640)	560 (435 to 705)	105 (68.4 to 158)	149.4 (91.5 to 229.6)	75.9 (31.9 to 137)
		Attributed age-standardized rate (per 100,000)	90.9 (71.9 to 111.1)	82.6 (63.8 to 103)	97.3 (74.2 to 124.2)	67.1 (55.2 to 81.9)	68.7 (54.2 to 84.6)	66.3 (51.5 to 83.6)	-26.1 (-38.1 to -9.8)	-16.8 (-34.8 to 6.5)	-31.9 (-47.3 to -10.6)
	DALYs	Attributed all ages number	15804 (12704 to 18934)	6492 (5156 to 7928)	9313 (7089 to 11831)	23774 (20119 to 27868)	11217 (9171 to 13460)	12557 (10106 to 15445)	50.4 (26.1 to 86.1)	72.8 (35.4 to 122.1)	34.8 (3.3 to 80.1)

Province	Measure	Age, Metric	Year						% Change (1990 to 2019)		
			1990			2019					
			Both	Female	Male	Both	Female	Male	Both	Female	Male
		Attributed age-standardized rate (per 100,000)	1928.9 (1545.9 to 2335.2)	1727.4 (1347.7 to 2105)	2088 (1586.8 to 2668.2)	1333.9 (1124.7 to 1573.1)	1297.1 (1064.1 to 1551.7)	1379.7 (1112.3 to 1703.3)	-30.8 (-41.7 to -15.6)	-24.9 (-40.4 to -3.6)	-33.9 (-48.8 to -13)
	YLLs	Attributed all ages number	14294 (11331 to 17409)	5668 (4383 to 7101)	8626 (6364 to 11084)	20387 (16992 to 24247)	9412 (7603 to 11456)	10975 (8630 to 13697)	42.6 (17.4 to 81)	66.1 (26.1 to 122.7)	27.2 (-4.6 to 74.2)
		Attributed age-standardized rate (per 100,000)	1776.6 (1402.3 to 2177.3)	1552.5 (1191.9 to 1924.8)	1956.3 (1466.2 to 2507)	1155.5 (959.9 to 1382.7)	1106.7 (891.1 to 1347.2)	1212.6 (956.2 to 1517.6)	-35 (-46.4 to -18.3)	-28.7 (-45.5 to -5.4)	-38 (-53.2 to -16.3)
	YLDs	Attributed all ages number	1511 (1093 to 1977)	824 (589 to 1077)	686 (490 to 912)	3387 (2469 to 4424)	1805 (1317 to 2375)	1582 (1145 to 2085)	124.2 (102.3 to 147.9)	119 (94.5 to 147.1)	130.4 (104.4 to 163.3)
		Attributed age-standardized rate (per 100,000)	152.4 (111.7 to 199.5)	174.9 (127.9 to 229.1)	131.7 (96.2 to 175.3)	178.5 (131.1 to 232.3)	190.4 (140.6 to 249.5)	167.1 (122.2 to 221.5)	17.1 (8.2 to 26.1)	8.9 (-0.8 to 19.6)	26.9 (14.4 to 39.8)
Khorasan-e-Razavi	Deaths	Attributed all ages number	1520 (1207 to 1876)	656 (506 to 822)	865 (655 to 1110)	2708 (2258 to 3268)	1316 (1062 to 1616)	1392 (1122 to 1749)	78.1 (46.1 to 116.1)	100.7 (54.4 to 156)	61 (21.2 to 112.1)
		Attributed age-standardized rate (per 100,000)	92.6 (73 to 114.8)	86.9 (67 to 111.5)	97 (73.2 to 124.3)	59.9 (49.4 to 72.9)	58.8 (46.7 to 72.9)	61.1 (48.8 to 76.7)	-35.3 (-45.8 to -23.2)	-32.4 (-46.9 to -15.2)	-37 (-51.7 to -19.4)
	DALYs	Attributed all ages number	46776 (38130 to 56304)	20520 (16222 to 25255)	26256 (20162 to 32812)	60666 (51851 to 70734)	28434 (23738 to 34116)	32232 (26615 to 38905)	29.7 (8.5 to 57.1)	38.6 (9.9 to 76.7)	22.8 (-5 to 57.6)
		Attributed age-standardized rate (per 100,000)	1916.9 (1556.8 to 2340.3)	1766.7 (1388 to 2194.9)	2043.6 (1575.1 to 2583)	1160.5 (987.1 to 1368.6)	1093.4 (905.5 to 1314.5)	1229.7 (1013.1 to 1496.7)	-39.5 (-49.3 to -28.3)	-38.1 (-50.7 to -22.4)	-39.8 (-53.4 to -23)
	YLLs	Attributed all ages number	42605 (34157 to 52117)	18353 (14062 to 22873)	24251 (18305 to 30989)	51455 (43824 to 60559)	23636 (19313 to 28762)	27819 (22573 to 34346)	20.8 (-0.6 to 50.4)	28.8 (-0.5 to 69.9)	14.7 (-13.5 to 51)
		Attributed age-standardized rate (per 100,000)	1766.7 (1396.1 to 2185.6)	1606.7 (1243.1 to 2020.1)	1903.7 (1448.1 to 2438.8)	1001.5 (843.9 to 1183.6)	929.9 (758.7 to 1132.9)	1074.9 (873.7 to 1334.7)	-43.3 (-53.1 to -31.3)	-42.1 (-55.1 to -25.9)	-43.5 (-57.2 to -25.8)
	YLDs	Attributed all ages number	4172 (2978 to 5538)	2167 (1566 to 2883)	2005 (1424 to 2644)	9211 (6681 to 12088)	4798 (3519 to 6210)	4413 (3161 to 5942)	120.8 (99 to 144.5)	121.4 (95.8 to 148.8)	120.1 (93.5 to 153.2)

Province	Measure	Age, Metric	Year						% Change (1990 to 2019)		
			1990			2019					
			Both	Female	Male	Both	Female	Male	Both	Female	Male
		Attributed age-standardized rate (per 100,000)	150.2 (110.9 to 197.8)	160 (118.4 to 210.2)	139.9 (101.7 to 185.6)	159 (117 to 210.5)	163.5 (121 to 212.7)	154.8 (112.1 to 208.7)	5.9 (-2.1 to 14.8)	2.2 (-7.3 to 13.3)	10.6 (-0.8 to 23.4)
Khuzestan	Deaths	Attributed all ages number	824 (661 to 1013)	397 (310 to 494)	427 (328 to 556)	2002 (1617 to 2431)	984 (768 to 1216)	1019 (801 to 1276)	143 (99.7 to 192.5)	147.6 (93.9 to 219.9)	138.7 (82.6 to 210.5)
		Attributed age-standardized rate (per 100,000)	81.2 (64.5 to 100.5)	78.6 (60.5 to 98.7)	82.8 (63.4 to 107.1)	68.4 (54.4 to 83.6)	69.3 (53.5 to 86.5)	67.7 (53 to 85.6)	-15.7 (-29.3 to -0.5)	-11.9 (-30 to 12.1)	-18.2 (-36.4 to 4.5)
	DALYs	Attributed all ages number	23359 (19626 to 27788)	10868 (8852 to 13220)	12491 (9928 to 15929)	46507 (39092 to 53868)	22202 (18117 to 26438)	24305 (19801 to 29487)	99.1 (65.8 to 135.2)	104.3 (64.8 to 157.5)	94.6 (52.1 to 151)
		Attributed age-standardized rate (per 100,000)	1679 (1365.1 to 2043.1)	1606.9 (1272.8 to 1980.5)	1733.3 (1349.2 to 2222.4)	1336.1 (1119.9 to 1565.1)	1301.7 (1052.7 to 1560.2)	1371.1 (1115 to 1667.3)	-20.4 (-33.6 to -6)	-19 (-35 to 1.9)	-20.9 (-38.4 to 1.3)
	YLLs	Attributed all ages number	20715 (17122 to 24872)	9354 (7380 to 11637)	11361 (8899 to 14757)	39413 (32776 to 46662)	18372 (14731 to 22402)	21041 (16896 to 26196)	90.3 (54.5 to 129.6)	96.4 (52.5 to 157.2)	85.2 (40.9 to 144.8)
		Attributed age-standardized rate (per 100,000)	1527.7 (1226.6 to 1875.1)	1433.7 (1110.3 to 1785.4)	1604.7 (1234 to 2089.7)	1158.1 (954.6 to 1383.5)	1111.5 (889.3 to 1361.5)	1205 (959.5 to 1496.9)	-24.2 (-37.8 to -8.7)	-22.5 (-39.2 to 0.7)	-24.9 (-42.6 to -1.6)
	YLDs	Attributed all ages number	2643 (1892 to 3460)	1514 (1093 to 2016)	1130 (795 to 1521)	7093 (5138 to 9226)	3830 (2781 to 5004)	3264 (2337 to 4314)	168.3 (142.1 to 197.1)	153 (124.3 to 186.8)	188.9 (154.1 to 228.7)
		Attributed age-standardized rate (per 100,000)	151.3 (111.7 to 196.9)	173.3 (126.4 to 224.4)	128.6 (93 to 170.5)	178.1 (131.2 to 231)	190.2 (140 to 248.8)	166.1 (119.9 to 217.9)	17.7 (8.6 to 27.1)	9.8 (-1 to 21.3)	29.2 (16.8 to 42.5)
Kohgiluyeh and Boyer-Ahmad	Deaths	Attributed all ages number	78 (62 to 97)	38 (29 to 48)	40 (29 to 55)	248 (191 to 309)	116 (88 to 149)	131 (96 to 172)	217.6 (149.7 to 302.9)	210 (131.2 to 321)	224.7 (131.8 to 351.5)
		Attributed age-standardized rate (per 100,000)	68.8 (53.8 to 87)	70.2 (53.3 to 90.8)	66.2 (47.7 to 89.4)	60.3 (45.8 to 76)	66.9 (49.5 to 86.2)	55.9 (40.8 to 73.2)	-12.5 (-29.4 to 7.3)	-4.7 (-27.3 to 24.2)	-15.5 (-37 to 14.6)
	DALYs	Attributed all ages number	2648 (2158 to 3197)	1238 (986 to 1509)	1409 (1044 to 1827)	5519 (4494 to 6604)	2599 (2070 to 3202)	2920 (2266 to 3644)	108.4 (67.5 to 158)	109.9 (63.2 to 177)	107.2 (53.7 to 184.1)

Province	Measure	Age, Metric	Year						% Change (1990 to 2019)		
			1990			2019					
			Both	Female	Male	Both	Female	Male	Both	Female	Male
		Attributed age-standardized rate (per 100,000)	1364.4 (1102.1 to 1698.6)	1360.3 (1071.8 to 1709.3)	1347.4 (983.7 to 1774.4)	1119.5 (903.3 to 1365.2)	1175.5 (935.5 to 1455.9)	1086.7 (835 to 1369.7)	-18 (-32.8 to 1)	-13.6 (-33.2 to 13.4)	-19.3 (-40 to 9.3)
	YLLs	Attributed all ages number	2313 (1828 to 2841)	1051 (812 to 1308)	1262 (915 to 1654)	4568 (3640 to 5595)	2102 (1614 to 2687)	2466 (1835 to 3179)	97.5 (54.7 to 154)	100 (49.5 to 178.4)	95.4 (39.8 to 180.6)
		Attributed age-standardized rate (per 100,000)	1228.7 (975.1 to 1542.9)	1206.6 (922.2 to 1540)	1229.9 (888.7 to 1666.6)	956 (752.4 to 1179)	1000.5 (766.2 to 1278.1)	933.5 (690.8 to 1205.3)	-22.2 (-38.2 to -1.3)	-17.1 (-38 to 13.5)	-24.1 (-44.5 to 6.8)
	YLDs	Attributed all ages number	335 (239 to 436)	188 (133 to 247)	147 (105 to 197)	951 (683 to 1249)	497 (356 to 649)	454 (327 to 610)	183.8 (152.5 to 219.6)	165 (130.2 to 201.2)	207.8 (165.3 to 253.6)
		Attributed age-standardized rate (per 100,000)	135.7 (99.7 to 177.9)	153.7 (111.4 to 201.2)	117.5 (85.8 to 155.2)	163.5 (120.5 to 213.6)	175.1 (128.7 to 225.9)	153.2 (110.7 to 205.3)	20.5 (11.6 to 29.9)	13.9 (2.9 to 26.2)	30.4 (18.1 to 44)
Kurdistan	Deaths	Attributed all ages number	379 (307 to 458)	158 (125 to 197)	221 (169 to 278)	738 (612 to 878)	353 (283 to 423)	385 (304 to 483)	94.9 (63.8 to 135.1)	123.4 (78.2 to 177.3)	74.5 (34.2 to 128.3)
		Attributed age-standardized rate (per 100,000)	85.5 (69 to 104.7)	78.7 (61.1 to 98.9)	91 (69.8 to 116.3)	59.4 (48.9 to 70.9)	60.3 (47.9 to 72.7)	59.3 (46.8 to 74.4)	-30.5 (-40.5 to -17.4)	-23.4 (-38.2 to -6.4)	-34.8 (-49.4 to -16.6)
	DALYs	Attributed all ages number	11485 (9408 to 13631)	5040 (3980 to 6211)	6444 (4948 to 8102)	16097 (13822 to 18554)	7443 (6222 to 8732)	8653 (7089 to 10573)	40.2 (18.8 to 68.1)	47.7 (18.5 to 86.2)	34.3 (3.8 to 75)
		Attributed age-standardized rate (per 100,000)	1810.2 (1493.4 to 2170.4)	1680.8 (1366.2 to 2068)	1912.7 (1485.4 to 2404.4)	1164.8 (996.8 to 1348.1)	1114.9 (929 to 1307)	1223.5 (1001.3 to 1495.6)	-35.7 (-44.7 to -24.2)	-33.7 (-45.4 to -19.1)	-36 (-50 to -18.1)
	YLLs	Attributed all ages number	10433 (8375 to 12608)	4461 (3386 to 5683)	5972 (4501 to 7619)	13528 (11520 to 15807)	6093 (5002 to 7253)	7435 (5951 to 9246)	29.7 (7.1 to 59)	36.6 (5.8 to 77.1)	24.5 (-6.1 to 66.3)
		Attributed age-standardized rate (per 100,000)	1664.9 (1353.6 to 2007.3)	1513.6 (1199.2 to 1879.1)	1787.3 (1367.6 to 2261)	994.9 (843.1 to 1165.1)	935.1 (764.1 to 1110.5)	1062.8 (846.3 to 1322.7)	-40.2 (-49.5 to -28.1)	-38.2 (-50.8 to -22.5)	-40.5 (-55 to -21.9)
	YLDs	Attributed all ages number	1052 (764 to 1375)	579 (422 to 756)	472 (340 to 631)	2569 (1852 to 3361)	1351 (972 to 1767)	1218 (875 to 1606)	144.3 (120.2 to 170.8)	133.2 (105.9 to 164.2)	157.9 (127.9 to 193.1)

Province	Measure	Age, Metric	Year						% Change (1990 to 2019)		
			1990			2019					
			Both	Female	Male	Both	Female	Male	Both	Female	Male
		Attributed age-standardized rate (per 100,000)	145.3 (108.2 to 191)	167.1 (124.2 to 218.5)	125.4 (91.2 to 167.7)	169.9 (123.8 to 223.7)	179.9 (131.6 to 235.7)	160.7 (116.4 to 214.2)	16.9 (7.8 to 26)	7.6 (-2.9 to 19.3)	28.2 (15.8 to 41.8)
Lorestan	Deaths	Attributed all ages number	364 (291 to 444)	146 (113 to 182)	218 (164 to 281)	856 (692 to 1034)	339 (261 to 415)	518 (410 to 640)	135.5 (94.4 to 186.4)	131.8 (77.3 to 199.1)	137.9 (81.1 to 213.3)
		Attributed age-standardized rate (per 100,000)	76.1 (59.9 to 93)	68 (51.7 to 85.8)	83.2 (63.1 to 105.6)	66.2 (53 to 80.5)	52.8 (40.7 to 65.3)	80.1 (63.3 to 99.2)	-13 (-27.2 to 4.2)	-22.3 (-39.2 to -2.3)	-3.6 (-25 to 24.8)
	DALYs	Attributed all ages number	10573 (8725 to 12674)	4313 (3449 to 5237)	6260 (4833 to 7980)	18163 (15142 to 21439)	7244 (5760 to 8681)	10918 (8844 to 13333)	71.8 (42.6 to 106.3)	68 (31.4 to 113.8)	74.4 (36.1 to 124.3)
		Attributed age-standardized rate (per 100,000)	1563.7 (1271.6 to 1897.9)	1382.4 (1098.1 to 1689.4)	1712.2 (1319.6 to 2168.8)	1253.8 (1042.3 to 1478.8)	993 (790.4 to 1186.4)	1528.8 (1241.6 to 1872.8)	-19.8 (-32.6 to -3.7)	-28.2 (-43.3 to -10.2)	-10.7 (-30.4 to 15.6)
	YLLs	Attributed all ages number	9399 (7561 to 11437)	3675 (2855 to 4557)	5724 (4300 to 7399)	15546 (12772 to 18701)	5865 (4568 to 7185)	9680 (7736 to 11996)	65.4 (34.2 to 103.7)	59.6 (18.4 to 111.4)	69.1 (28.4 to 125.2)
		Attributed age-standardized rate (per 100,000)	1425.1 (1137.8 to 1739.4)	1226.4 (952.6 to 1524.1)	1590.6 (1206.2 to 2055.9)	1089.2 (897.1 to 1309.2)	821.7 (639.4 to 1000.8)	1370.2 (1092.5 to 1695.7)	-23.6 (-36.9 to -6.5)	-33 (-49.1 to -13.4)	-13.9 (-34.2 to 14.3)
	YLDs	Attributed all ages number	1174 (858 to 1555)	637 (462 to 835)	536 (382 to 708)	2617 (1889 to 3419)	1379 (1006 to 1804)	1238 (887 to 1623)	123 (100.3 to 148.1)	116.4 (87.6 to 148.2)	130.9 (103 to 165.7)
		Attributed age-standardized rate (per 100,000)	138.6 (102.7 to 181.4)	155.9 (115.4 to 203.7)	121.6 (88.4 to 161.4)	164.6 (120.6 to 215)	171.2 (125.3 to 223.2)	158.6 (114.7 to 207.7)	18.7 (9.1 to 28.1)	9.8 (-1.7 to 21.8)	30.4 (17.7 to 44.7)
Markazi	Deaths	Attributed all ages number	392 (313 to 473)	177 (137 to 220)	215 (162 to 275)	799 (649 to 969)	396 (311 to 490)	403 (317 to 506)	103.9 (63.9 to 151.9)	123.5 (71.3 to 197.3)	87.7 (40.8 to 151)
		Attributed age-standardized rate (per 100,000)	83.2 (66.5 to 100.7)	80 (61.6 to 100)	85.7 (64.9 to 109.7)	53.9 (43.7 to 65.2)	54.5 (42.8 to 67.4)	53.5 (42.1 to 67.6)	-35.2 (-46 to -22.2)	-31.9 (-46.1 to -12.4)	-37.5 (-51.8 to -18.8)
	DALYs	Attributed all ages number	11364 (9165 to 13461)	5201 (4196 to 6296)	6163 (4666 to 7830)	16050 (13568 to 18775)	7706 (6399 to 9126)	8344 (6794 to 10194)	41.2 (15.9 to 72.7)	48.2 (17.6 to 91.3)	35.4 (4.2 to 79.8)

Province	Measure	Age, Metric	Year						% Change (1990 to 2019)		
			1990			2019					
			Both	Female	Male	Both	Female	Male	Both	Female	Male
		Attributed age-standardized rate (per 100,000)	1734.4 (1396.4 to 2067.7)	1641.6 (1312.5 to 1998.5)	1816.1 (1394.7 to 2292.5)	1079 (910.2 to 1261.9)	1040.9 (866.5 to 1235.6)	1118.4 (909.7 to 1368.8)	-37.8 (-47.8 to -25.3)	-36.6 (-49.2 to -19.2)	-38.4 (-52.5 to -19.9)
	YLLs	Attributed all ages number	10157 (8066 to 12110)	4518 (3523 to 5573)	5640 (4179 to 7318)	13401 (11134 to 15944)	6293 (5090 to 7623)	7108 (5642 to 8926)	31.9 (5.5 to 65.2)	39.3 (6 to 87.9)	26 (-6 to 73.8)
		Attributed age-standardized rate (per 100,000)	1575.8 (1256.2 to 1895.8)	1460.7 (1134.6 to 1810)	1680.2 (1267 to 2153.9)	903.2 (752.3 to 1073.8)	853.5 (692.2 to 1030.2)	953.7 (756.5 to 1194.3)	-42.7 (-53.1 to -29.6)	-41.6 (-54.7 to -23)	-43.2 (-57.4 to -23.8)
	YLDs	Attributed all ages number	1207 (874 to 1568)	683 (505 to 899)	523 (371 to 699)	2649 (1943 to 3434)	1413 (1037 to 1840)	1236 (896 to 1622)	119.5 (98.1 to 145.2)	106.8 (83 to 134)	136.1 (105.6 to 171.5)
		Attributed age-standardized rate (per 100,000)	158.5 (116.8 to 206)	180.9 (132.5 to 237.1)	135.9 (98.8 to 178.5)	175.9 (128.5 to 228.5)	187.5 (138.8 to 246.1)	164.6 (119.2 to 216.9)	10.9 (2.7 to 19.1)	3.6 (-6.4 to 14.3)	21.1 (9.1 to 33.3)
Mazandaran	Deaths	Attributed all ages number	632 (505 to 770)	325 (246 to 415)	307 (227 to 404)	1802 (1474 to 2174)	914 (716 to 1139)	888 (692 to 1107)	185.2 (135.3 to 249.2)	181.1 (115.7 to 265.3)	189.6 (113.8 to 294.8)
		Attributed age-standardized rate (per 100,000)	75.5 (59.1 to 92.3)	77.8 (58.4 to 100.4)	71.9 (54.1 to 93.2)	57 (46.5 to 69)	57.8 (45.2 to 72)	56.1 (43.8 to 70.1)	-24.5 (-36.7 to -8.4)	-25.7 (-41.5 to -4.9)	-22 (-40.5 to 3.2)
	DALYs	Attributed all ages number	17563 (14593 to 20928)	8740 (6885 to 10872)	8823 (6841 to 11295)	38771 (32490 to 45430)	18994 (15551 to 22640)	19777 (16064 to 24281)	120.8 (85.7 to 164.1)	117.3 (71.7 to 171.2)	124.2 (69.1 to 195)
		Attributed age-standardized rate (per 100,000)	1472.9 (1198.8 to 1773.4)	1503.1 (1158.4 to 1884.4)	1429 (1109.5 to 1839.9)	1115.1 (929.4 to 1303.3)	1097.1 (900.9 to 1310.3)	1133 (916.9 to 1392.5)	-24.3 (-35.9 to -9.3)	-27 (-41.9 to -8.1)	-20.7 (-39.4 to 4.1)
	YLLs	Attributed all ages number	15229 (12308 to 18413)	7399 (5634 to 9439)	7830 (5893 to 10206)	32629 (26876 to 38733)	15732 (12693 to 19230)	16897 (13339 to 20983)	114.3 (75.3 to 164.2)	112.6 (61.1 to 176.7)	115.8 (56.2 to 196.2)
		Attributed age-standardized rate (per 100,000)	1321.1 (1054.1 to 1602.5)	1330.5 (1009.5 to 1702.7)	1299 (972.7 to 1693.5)	944.3 (780.2 to 1126)	917.1 (729.2 to 1123.2)	971.2 (765.9 to 1206.8)	-28.5 (-40.6 to -12.2)	-31.1 (-47.2 to -10.2)	-25.2 (-44.8 to 1.6)
	YLDs	Attributed all ages number	2334 (1667 to 3057)	1342 (957 to 1760)	992 (709 to 1325)	6142 (4489 to 8037)	3262 (2374 to 4249)	2880 (2067 to 3854)	163.2 (136.7 to 192.1)	143.2 (114.3 to 176.3)	190.2 (154.8 to 231.3)

Province	Measure	Age, Metric	Year						% Change (1990 to 2019)		
			1990			2019					
			Both	Female	Male	Both	Female	Male	Both	Female	Male
		Attributed age-standardized rate (per 100,000)	151.8 (112.1 to 196.3)	172.6 (126.4 to 224)	130 (94.3 to 171.1)	170.8 (124.9 to 222.6)	180 (131.4 to 235.2)	161.8 (117.1 to 216.5)	12.5 (3.6 to 21.8)	4.3 (-6.3 to 15.5)	24.4 (12.5 to 37.5)
North Khorasan	Deaths	Attributed all ages number	164 (135 to 196)	71 (57 to 88)	92 (73 to 117)	392 (323 to 466)	196 (160 to 238)	197 (159 to 239)	139.7 (100.3 to 186.4)	174.5 (117.9 to 245.2)	112.9 (67.2 to 172)
		Attributed age-standardized rate (per 100,000)	77.5 (63.2 to 93.3)	74.7 (57.8 to 93.4)	79.2 (62.1 to 99.4)	62.1 (50.6 to 73.8)	66.4 (53.3 to 81.8)	58.9 (47.5 to 71.3)	-19.9 (-32.1 to -5.8)	-11.2 (-28.4 to 9.8)	-25.6 (-40.3 to -7.6)
	DALYs	Attributed all ages number	5509 (4631 to 6514)	2458 (2029 to 2977)	3051 (2406 to 3806)	8799 (7557 to 10181)	4370 (3703 to 5152)	4429 (3697 to 5273)	59.7 (34.7 to 88.9)	77.8 (43 to 120.9)	45.2 (15.3 to 85.4)
		Attributed age-standardized rate (per 100,000)	1687.2 (1411.3 to 1990.2)	1605 (1296 to 1937.3)	1749.3 (1396 to 2162.7)	1254.4 (1073.5 to 1456.6)	1282.9 (1082.7 to 1515.9)	1236.8 (1029.1 to 1475.4)	-25.7 (-36.7 to -12.9)	-20.1 (-34.3 to -2.2)	-29.3 (-42.7 to -12.1)
	YLLs	Attributed all ages number	4984 (4109 to 5932)	2162 (1735 to 2693)	2822 (2172 to 3593)	7498 (6355 to 8728)	3669 (3043 to 4379)	3829 (3135 to 4634)	50.4 (24.9 to 81.8)	69.7 (31.3 to 119)	35.7 (5.7 to 78.1)
		Attributed age-standardized rate (per 100,000)	1542 (1279.5 to 1847.2)	1437.9 (1133.6 to 1773.5)	1625.9 (1286.5 to 2043.2)	1083.6 (908.4 to 1273.2)	1098.9 (905.1 to 1319.7)	1078.6 (879.5 to 1310.3)	-29.7 (-40.8 to -16.4)	-23.6 (-38.7 to -4.7)	-33.7 (-47.3 to -15.6)
	YLDs	Attributed all ages number	525 (382 to 690)	296 (216 to 387)	229 (164 to 306)	1301 (953 to 1700)	700 (513 to 901)	601 (432 to 805)	147.7 (124.8 to 175)	136.4 (109.9 to 166.9)	162.2 (130.6 to 198.1)
		Attributed age-standardized rate (per 100,000)	145.2 (108.1 to 190.4)	167.1 (124 to 219.5)	123.5 (90.2 to 162.7)	170.8 (125.6 to 220.5)	184 (135.8 to 238.5)	158.3 (114 to 209.9)	17.6 (8.2 to 26.9)	10.1 (-0.3 to 22)	28.2 (15.8 to 40.7)
Qazvin	Deaths	Attributed all ages number	224 (179 to 275)	104 (80 to 132)	120 (90 to 156)	568 (466 to 681)	247 (193 to 308)	321 (258 to 386)	153.7 (109.1 to 213.4)	138 (84.2 to 213.4)	167.4 (105.1 to 256.5)
		Attributed age-standardized rate (per 100,000)	68.8 (54.1 to 86)	64.9 (49.2 to 83.5)	72.9 (54.5 to 94.2)	60.1 (48.8 to 72.4)	50.3 (38.9 to 62.7)	70.8 (56.7 to 86.1)	-12.7 (-27.3 to 5.1)	-22.5 (-39.7 to 0.6)	-2.9 (-23 to 25.2)
	DALYs	Attributed all ages number	6563 (5349 to 7787)	3062 (2468 to 3811)	3501 (2710 to 4442)	12469 (10507 to 14552)	5298 (4376 to 6359)	7171 (5883 to 8456)	90 (60.6 to 129.5)	73 (38.7 to 119.6)	104.8 (59.2 to 165.8)

Province	Measure	Age, Metric	Year						% Change (1990 to 2019)		
			1990			2019					
			Both	Female	Male	Both	Female	Male	Both	Female	Male
		Attributed age-standardized rate (per 100,000)	1424.2 (1153.1 to 1710.7)	1356.2 (1070.3 to 1698.4)	1487 (1142.5 to 1888.2)	1149.7 (963.9 to 1349.7)	957.3 (786 to 1153)	1353.5 (1111.1 to 1611.6)	-19.3 (-31.9 to -2.8)	-29.4 (-43.4 to -11.2)	-9 (-27.7 to 18.4)
	YLLs	Attributed all ages number	5780 (4623 to 6993)	2616 (2061 to 3301)	3164 (2382 to 4073)	10454 (8664 to 12402)	4220 (3363 to 5182)	6234 (5059 to 7512)	80.9 (48.8 to 126.1)	61.3 (24.4 to 114.4)	97 (49.4 to 163.4)
		Attributed age-standardized rate (per 100,000)	1282.5 (1028.5 to 1572.5)	1193.5 (928.7 to 1513.7)	1366.8 (1031.8 to 1761.4)	980.9 (810.9 to 1164.2)	779.6 (616.2 to 965.6)	1193.5 (967.9 to 1439.5)	-23.5 (-36.8 to -5.6)	-34.7 (-49.7 to -14.1)	-12.7 (-32.1 to 15.7)
	YLDs	Attributed all ages number	783 (565 to 1036)	446 (321 to 589)	337 (236 to 450)	2015 (1453 to 2654)	1078 (779 to 1417)	937 (673 to 1241)	157.3 (132 to 185.8)	141.6 (112.7 to 173.1)	178 (141.8 to 218.5)
		Attributed age-standardized rate (per 100,000)	141.7 (104.4 to 186.2)	162.6 (120.1 to 211.6)	120.2 (86.8 to 162.3)	168.8 (122.2 to 222.4)	177.7 (129.2 to 232.3)	160 (115.5 to 211.8)	19.1 (10.1 to 28.3)	9.3 (-1.5 to 20.8)	33.1 (20.1 to 47.5)
Qom	Deaths	Attributed all ages number	186 (144 to 228)	90 (68 to 112)	97 (71 to 124)	525 (430 to 627)	260 (208 to 314)	265 (205 to 330)	181.3 (128.9 to 265.5)	188.9 (123.8 to 296.2)	174.2 (109.2 to 283.3)
		Attributed age-standardized rate (per 100,000)	81.9 (65.5 to 100)	83.9 (63.1 to 105.4)	78.6 (58.7 to 100.1)	62.3 (50.6 to 75.1)	72.5 (56.7 to 88)	56.4 (43.8 to 70.2)	-23.9 (-37.2 to -5.2)	-13.5 (-31.8 to 10.6)	-28.3 (-43.7 to -3.9)
	DALYs	Attributed all ages number	5491 (4255 to 6592)	2601 (1956 to 3174)	2889 (2121 to 3676)	11489 (9755 to 13388)	5546 (4670 to 6471)	5943 (4823 to 7184)	109.2 (74.4 to 166.1)	113.2 (70.4 to 188.8)	105.7 (59.3 to 176.4)
		Attributed age-standardized rate (per 100,000)	1647 (1294.1 to 1982)	1632.5 (1268.8 to 2006.4)	1643.1 (1241.1 to 2082.9)	1157.8 (976.7 to 1363.5)	1229.8 (1027.5 to 1447.9)	1115.4 (900.2 to 1357)	-29.7 (-40.9 to -12.5)	-24.7 (-39.4 to -1.3)	-32.1 (-47 to -9.5)
	YLLs	Attributed all ages number	4848 (3627 to 5907)	2238 (1616 to 2773)	2610 (1843 to 3384)	9494 (7937 to 11195)	4496 (3664 to 5342)	4998 (3945 to 6139)	95.8 (60.1 to 159.7)	100.9 (54.5 to 193)	91.5 (44.7 to 166.1)
		Attributed age-standardized rate (per 100,000)	1491.5 (1153.3 to 1815)	1454.4 (1092.4 to 1817.2)	1509.9 (1118.8 to 1945.9)	984.2 (818.3 to 1167.3)	1043.5 (846.3 to 1252)	953.8 (751 to 1183)	-34 (-45.8 to -15.3)	-28.3 (-44.2 to -2.6)	-36.8 (-51.7 to -13.1)
	YLDs	Attributed all ages number	642 (460 to 843)	363 (265 to 476)	280 (196 to 366)	1994 (1438 to 2631)	1049 (759 to 1379)	945 (679 to 1252)	210.4 (180 to 242)	189.1 (155.5 to 226.1)	238.1 (196.7 to 284.6)

Province	Measure	Age, Metric	Year						% Change (1990 to 2019)		
			1990			2019					
			Both	Female	Male	Both	Female	Male	Both	Female	Male
		Attributed age-standardized rate (per 100,000)	155.5 (115.5 to 202.5)	178.1 (131 to 230.6)	133.3 (97.3 to 176.7)	173.5 (126.4 to 228.3)	186.3 (134.9 to 244.7)	161.6 (115.9 to 212.7)	11.6 (3.3 to 20.1)	4.6 (-5.1 to 15.7)	21.2 (9.1 to 33.5)
Semnan	Deaths	Attributed all ages number	163 (129 to 201)	73 (55 to 92)	91 (68 to 117)	363 (298 to 435)	161 (125 to 197)	203 (163 to 245)	122.2 (84.4 to 176)	121.4 (71.6 to 192.6)	122.8 (73.2 to 199.1)
		Attributed age-standardized rate (per 100,000)	77.7 (62.8 to 94.5)	68.9 (52.7 to 87.6)	87.9 (67.7 to 110.8)	62.6 (50.8 to 75.3)	52.6 (40.8 to 64.5)	73.5 (59.1 to 89.5)	-19.4 (-32.2 to -2.6)	-23.7 (-40.1 to -2.5)	-16.3 (-33 to 6.7)
	DALYs	Attributed all ages number	4505 (3667 to 5422)	1967 (1522 to 2438)	2538 (1945 to 3200)	7852 (6626 to 9094)	3364 (2743 to 4016)	4488 (3752 to 5310)	74.3 (46 to 113.1)	71 (37.1 to 120.9)	76.8 (39.4 to 132.3)
		Attributed age-standardized rate (per 100,000)	1613.4 (1318.6 to 1941.5)	1427.9 (1125.7 to 1763.4)	1805.4 (1399.1 to 2268.2)	1206.6 (1017.3 to 1403.4)	1009 (820.2 to 1210.8)	1413 (1175.7 to 1683.4)	-25.2 (-36.8 to -9.8)	-29.3 (-43.6 to -10.5)	-21.7 (-37.4 to 2.5)
	YLLs	Attributed all ages number	4004 (3148 to 4893)	1687 (1251 to 2121)	2316 (1736 to 2978)	6607 (5501 to 7812)	2700 (2158 to 3295)	3907 (3181 to 4686)	65 (35.2 to 108.2)	60 (22.6 to 117.8)	68.7 (30.2 to 127.2)
		Attributed age-standardized rate (per 100,000)	1457.5 (1168.6 to 1773.9)	1253.9 (949.3 to 1579.5)	1668.6 (1260.1 to 2137.6)	1030.1 (853.3 to 1221)	823.8 (656.7 to 1011.4)	1245.1 (1012.2 to 1497.3)	-29.3 (-41.3 to -12.9)	-34.3 (-49.5 to -12.8)	-25.4 (-41.2 to 0.2)
	YLDs	Attributed all ages number	501 (368 to 665)	280 (206 to 371)	222 (159 to 295)	1245 (916 to 1649)	664 (479 to 877)	581 (423 to 777)	148.2 (125.1 to 173.5)	137.3 (110.8 to 167.1)	162 (132.4 to 196.4)
		Attributed age-standardized rate (per 100,000)	155.9 (115.6 to 204.8)	174.1 (128.6 to 226.7)	136.8 (99.8 to 182.6)	176.5 (130.6 to 233.1)	185.1 (134.8 to 243.6)	168 (121.9 to 226.7)	13.2 (4.6 to 22.3)	6.3 (-4.1 to 17.4)	22.8 (10.9 to 35.6)
Sistan and Baluchistan	Deaths	Attributed all ages number	392 (268 to 478)	156 (115 to 197)	237 (148 to 306)	961 (804 to 1145)	452 (358 to 556)	510 (409 to 630)	145 (95.1 to 261.8)	190 (115.3 to 303.1)	115.5 (56.4 to 245)
		Attributed age-standardized rate (per 100,000)	87.8 (67.8 to 107.6)	80.7 (61.2 to 102.7)	93.7 (68.2 to 122)	78.8 (65.2 to 94.4)	76.5 (60.5 to 94.2)	81.5 (64.9 to 101)	-10.2 (-27.8 to 15.6)	-5.2 (-29.7 to 26.6)	-13.1 (-35.6 to 23.2)
	DALYs	Attributed all ages number	13265 (8293 to 16085)	5380 (3413 to 6718)	7886 (4731 to 9996)	26533 (22882 to 30584)	12504 (10383 to 14914)	14029 (11537 to 16857)	100 (58.6 to 216.1)	132.4 (74.4 to 273.5)	77.9 (31.8 to 202.3)

Province	Measure	Age, Metric	Year						% Change (1990 to 2019)		
			1990			2019					
			Both	Female	Male	Both	Female	Male	Both	Female	Male
		Attributed age-standardized rate (per 100,000)	1910.4 (1378.7 to 2301.8)	1731.3 (1322.7 to 2166.5)	2051.8 (1365.4 to 2627.9)	1729.3 (1478.3 to 2006.4)	1662.2 (1357.8 to 1995.7)	1802.2 (1463.5 to 2211.1)	-9.5 (-26.5 to 24.8)	-4 (-27.3 to 28.4)	-12.2 (-34.2 to 30)
	YLLs	Attributed all ages number	12096 (7322 to 14871)	4749 (2837 to 6038)	7348 (4157 to 9438)	23012 (19560 to 26670)	10570 (8548 to 12946)	12442 (10056 to 15255)	90.2 (49 to 219.2)	122.6 (61.9 to 289.6)	69.3 (22.5 to 206)
		Attributed age-standardized rate (per 100,000)	1761.3 (1229.9 to 2152.5)	1562.7 (1164 to 1995.3)	1920.9 (1245.7 to 2486.2)	1543.5 (1302.2 to 1815.6)	1463.5 (1173.7 to 1803)	1628.8 (1300.5 to 2014.4)	-12.4 (-30.2 to 24.5)	-6.3 (-30.9 to 29.7)	-15.2 (-38 to 29.6)
	YLDs	Attributed all ages number	1169 (838 to 1547)	631 (447 to 829)	538 (380 to 719)	3521 (2512 to 4658)	1933 (1381 to 2559)	1587 (1124 to 2132)	201.1 (174.2 to 233.2)	206.4 (170.6 to 247.4)	194.9 (159.9 to 232.4)
		Attributed age-standardized rate (per 100,000)	149 (109.8 to 195.8)	168.7 (124.1 to 221.4)	130.9 (95.3 to 173.6)	185.8 (135.6 to 243.3)	198.7 (145.6 to 259.5)	173.4 (124.4 to 230.5)	24.7 (16 to 34)	17.8 (6.5 to 30.3)	32.5 (18.5 to 46.3)
South Khorasan	Deaths	Attributed all ages number	175 (143 to 212)	76 (61 to 96)	98 (76 to 125)	344 (279 to 413)	168 (134 to 208)	176 (141 to 216)	96.7 (62.5 to 135.5)	119.8 (72.9 to 179.4)	78.8 (37.9 to 136.4)
		Attributed age-standardized rate (per 100,000)	63.3 (51.6 to 77.7)	59.1 (46.1 to 75.1)	67.2 (52.4 to 85.1)	50.1 (40.7 to 60.1)	47 (37.5 to 58.2)	54.2 (43 to 66.4)	-20.8 (-33.1 to -7.3)	-20.4 (-37.5 to 0.9)	-19.3 (-35.7 to 3.2)
	DALYs	Attributed all ages number	5427 (4485 to 6457)	2413 (1957 to 2941)	3015 (2308 to 3778)	6980 (5944 to 8096)	3350 (2808 to 3972)	3630 (3006 to 4343)	28.6 (7.7 to 54.2)	38.9 (13.5 to 71)	20.4 (-6.5 to 58.2)
		Attributed age-standardized rate (per 100,000)	1352.6 (1129.5 to 1623.7)	1271.7 (1041.4 to 1577)	1423 (1130 to 1771.6)	973.4 (828.3 to 1131)	915.6 (766 to 1088.6)	1042 (861.5 to 1247.1)	-28 (-38.3 to -16.4)	-28 (-41.5 to -12.8)	-26.8 (-41.6 to -6.6)
	YLLs	Attributed all ages number	4833 (3908 to 5843)	2082 (1646 to 2589)	2752 (2031 to 3500)	5750 (4818 to 6780)	2684 (2208 to 3262)	3066 (2504 to 3714)	19 (-2.5 to 46.9)	28.9 (1.3 to 64.8)	11.4 (-16.2 to 49.8)
		Attributed age-standardized rate (per 100,000)	1213.2 (1002.1 to 1470)	1112.3 (885.2 to 1396.5)	1303.4 (1019.4 to 1649.9)	811.5 (679.4 to 956.5)	743.3 (611.9 to 903.2)	889.7 (723.6 to 1079)	-33.1 (-43.9 to -20.9)	-33.2 (-47.4 to -16)	-31.7 (-47 to -10.9)
	YLDs	Attributed all ages number	594 (435 to 786)	331 (243 to 437)	263 (190 to 349)	1230 (898 to 1595)	666 (484 to 859)	563 (411 to 742)	107 (88.8 to 129.2)	101.4 (80.9 to 125.8)	114.1 (89 to 144.6)

Province	Measure	Age, Metric	Year						% Change (1990 to 2019)		
			1990			2019					
			Both	Female	Male	Both	Female	Male	Both	Female	Male
		Attributed age-standardized rate (per 100,000)	139.4 (103.8 to 182)	159.4 (119.4 to 209)	119.6 (87.5 to 156.9)	162 (118.5 to 212.2)	172.3 (125.9 to 223)	152.3 (110.9 to 201.2)	16.2 (7.9 to 25)	8.1 (-1.5 to 19.6)	27.4 (14.7 to 41)
Tehran	Deaths	Attributed all ages number	1414 (1116 to 1743)	719 (549 to 924)	694 (509 to 923)	4149 (3336 to 5016)	2148 (1683 to 2687)	2000 (1528 to 2505)	193.5 (133.7 to 265.3)	198.8 (126.8 to 295.7)	188 (109.3 to 294.2)
		Attributed age-standardized rate (per 100,000)	49.5 (38.8 to 61.5)	51.2 (38.2 to 66.3)	46.9 (34.5 to 61.8)	34.3 (27.4 to 41.7)	38.2 (29.7 to 47.7)	31.1 (23.7 to 39)	-30.7 (-44 to -13.9)	-25.4 (-43 to -1.6)	-33.7 (-51.2 to -10.5)
	DALYs	Attributed all ages number	40841 (33858 to 48961)	19855 (16177 to 24436)	20987 (16144 to 26897)	89814 (75285 to 106495)	44395 (36432 to 53536)	45419 (36695 to 55283)	119.9 (81.9 to 164.4)	123.6 (77.5 to 178.8)	116.4 (63.5 to 186.5)
		Attributed age-standardized rate (per 100,000)	985.1 (804.1 to 1194.6)	987.5 (780.4 to 1230.9)	968 (741.7 to 1247.7)	681.9 (571.4 to 809.8)	703.9 (572.8 to 854.1)	664.8 (536.6 to 810.7)	-30.8 (-42.4 to -16.7)	-28.7 (-43.4 to -10)	-31.3 (-48.6 to -9.6)
	YLLs	Attributed all ages number	33207 (26842 to 40524)	15680 (12175 to 19782)	17528 (13022 to 23315)	69023 (56752 to 82444)	33845 (26921 to 42066)	35178 (27348 to 43915)	107.9 (63.7 to 161.8)	115.9 (60 to 186)	100.7 (42.1 to 183.9)
		Attributed age-standardized rate (per 100,000)	838.5 (663.5 to 1032.5)	827.2 (636.6 to 1054.8)	835.4 (613.6 to 1110.7)	532.6 (437.3 to 639.2)	551.1 (438.1 to 684.3)	518.8 (403.6 to 649.5)	-36.5 (-49.2 to -20.8)	-33.4 (-49.8 to -11.6)	-37.9 (-55.6 to -13.8)
	YLDs	Attributed all ages number	7634 (5460 to 10115)	4175 (2964 to 5452)	3459 (2441 to 4597)	20791 (15213 to 27209)	10550 (7693 to 13814)	10241 (7324 to 13507)	172.4 (144.6 to 204.2)	152.7 (124.1 to 186.1)	196.1 (158.5 to 238.1)
		Attributed age-standardized rate (per 100,000)	146.6 (108.1 to 190.6)	160.3 (116.2 to 207.2)	132.5 (96.7 to 173.2)	149.4 (109.4 to 197.1)	152.8 (111.3 to 200.6)	146 (105 to 192.6)	1.8 (-6.5 to 10.4)	-4.7 (-14.4 to 6)	10.2 (-0.8 to 21.8)
West Azarbayejan	Deaths	Attributed all ages number	579 (471 to 701)	262 (205 to 336)	317 (240 to 406)	1420 (1157 to 1702)	714 (568 to 879)	706 (559 to 871)	145.2 (104.7 to 199.2)	172.6 (112.8 to 251.3)	122.5 (71.9 to 196.6)
		Attributed age-standardized rate (per 100,000)	88 (70.2 to 108.2)	85.4 (65.6 to 110.2)	89.9 (68.4 to 115)	72.8 (58.3 to 88)	73.2 (57.1 to 91.5)	72.5 (56.7 to 90.1)	-17.2 (-29.2 to -2.1)	-14.2 (-31.5 to 7.1)	-19.4 (-35.4 to 2.4)
	DALYs	Attributed all ages number	18104 (15055 to 21470)	8309 (6657 to 10164)	9795 (7654 to 12197)	31355 (26765 to 36556)	15178 (12640 to 17836)	16178 (13209 to 19543)	73.2 (46.1 to 109.5)	82.7 (45.8 to 130.2)	65.2 (31.5 to 115)

Province	Measure	Age, Metric	Year						% Change (1990 to 2019)		
			1990			2019					
			Both	Female	Male	Both	Female	Male	Both	Female	Male
		Attributed age-standardized rate (per 100,000)	1702.3 (1400.1 to 2029.7)	1640.2 (1294.3 to 2050)	1751.7 (1366.5 to 2195.4)	1268 (1064.2 to 1490.4)	1225.3 (1007.4 to 1460.3)	1311.3 (1068.8 to 1590.8)	-25.5 (-36 to -11.5)	-25.3 (-40 to -6.7)	-25.1 (-40.3 to -4.1)
	YLLs	Attributed all ages number	16215 (13201 to 19444)	7235 (5669 to 9094)	8981 (6829 to 11288)	26506 (22258 to 31253)	12519 (10236 to 15079)	13986 (11192 to 17127)	63.5 (36 to 103.4)	73 (33.2 to 126.4)	55.7 (20.5 to 106.3)
		Attributed age-standardized rate (per 100,000)	1558.9 (1267.5 to 1893)	1474.3 (1147.3 to 1899.6)	1630.3 (1251.1 to 2068.8)	1100.7 (911.6 to 1309.8)	1045.9 (849 to 1272.2)	1156.2 (921.9 to 1421.7)	-29.4 (-40.4 to -14.1)	-29.1 (-44.2 to -8.6)	-29.1 (-44.5 to -7.1)
	YLDs	Attributed all ages number	1889 (1366 to 2526)	1074 (772 to 1430)	814 (579 to 1094)	4850 (3486 to 6312)	2659 (1924 to 3447)	2191 (1549 to 2934)	156.8 (133.1 to 184.5)	147.4 (119.7 to 178.8)	169.1 (135.2 to 204.9)
		Attributed age-standardized rate (per 100,000)	143.4 (104.7 to 187.4)	165.9 (122.5 to 215.7)	121.4 (88.5 to 163.1)	167.2 (122.4 to 219)	179.4 (131.3 to 233.1)	155.2 (111.5 to 207.6)	16.7 (7.7 to 26.5)	8.2 (-2.7 to 18.8)	27.8 (14.2 to 42.3)
Yazd	Deaths	Attributed all ages number	187 (149 to 232)	92 (70 to 115)	95 (69 to 124)	465 (371 to 562)	225 (174 to 281)	240 (186 to 301)	148.2 (101 to 209)	145.2 (86 to 224.8)	151 (85.5 to 246.2)
		Attributed age-standardized rate (per 100,000)	73 (57.9 to 90.3)	68.3 (51.3 to 87.1)	77.8 (57.9 to 100.8)	57.1 (44.8 to 68.8)	53.9 (41.7 to 67.2)	60.7 (46.9 to 76)	-21.9 (-35.4 to -5.8)	-21.1 (-39.4 to 3.8)	-22 (-40.5 to 3.4)
	DALYs	Attributed all ages number	5069 (4144 to 6140)	2383 (1869 to 2942)	2686 (2005 to 3429)	9773 (8134 to 11543)	4475 (3658 to 5366)	5298 (4300 to 6511)	92.8 (60.1 to 138)	87.8 (48.2 to 142.4)	97.3 (52.7 to 161.9)
		Attributed age-standardized rate (per 100,000)	1445.7 (1163.3 to 1758.5)	1342.6 (1052.8 to 1661.5)	1547.9 (1166.7 to 1978.8)	1068.5 (884.4 to 1262.3)	985.1 (800.4 to 1183.9)	1149 (927.5 to 1414.6)	-26.1 (-37.8 to -10.4)	-26.6 (-42 to -6.3)	-25.8 (-42.5 to -2.6)
	YLLs	Attributed all ages number	4452 (3569 to 5464)	2034 (1556 to 2536)	2419 (1738 to 3137)	8036 (6606 to 9609)	3568 (2819 to 4403)	4469 (3500 to 5611)	80.5 (46 to 130.6)	75.4 (31.7 to 137.7)	84.8 (36.2 to 154.6)
		Attributed age-standardized rate (per 100,000)	1300.2 (1038.5 to 1603.7)	1179.2 (900.2 to 1481.4)	1421.3 (1050.5 to 1841.8)	898.1 (735.2 to 1074.4)	806.6 (635 to 994.8)	986.5 (771.7 to 1235.9)	-30.9 (-43.3 to -13.9)	-31.6 (-48.2 to -8.8)	-30.6 (-47.8 to -5.1)
	YLDs	Attributed all ages number	616 (450 to 816)	349 (254 to 460)	267 (192 to 354)	1737 (1255 to 2261)	907 (662 to 1178)	830 (587 to 1098)	181.8 (154.7 to 209.9)	159.8 (129.6 to 191.9)	210.6 (174 to 254.2)

Province	Measure	Age, Metric	Year						% Change (1990 to 2019)		
			1990			2019					
			Both	Female	Male	Both	Female	Male	Both	Female	Male
		Attributed age-standardized rate (per 100,000)	145.5 (107.2 to 190.7)	163.4 (119.9 to 213.2)	126.6 (92.6 to 167.8)	170.4 (123.8 to 221.6)	178.4 (130.9 to 231.8)	162.5 (115.6 to 216.9)	17.1 (7.9 to 26.6)	9.2 (-2.2 to 20)	28.4 (16 to 41.9)
Zanjan	Deaths	Attributed all ages number	186 (149 to 229)	81 (63 to 101)	105 (80 to 137)	490 (395 to 596)	222 (172 to 274)	268 (215 to 331)	163.7 (118 to 219.2)	173.9 (117 to 239.2)	155.8 (96.8 to 235.4)
		Attributed age-standardized rate (per 100,000)	62 (48.6 to 77.1)	55.7 (41.9 to 70.9)	69 (50.9 to 89.1)	54.6 (43.9 to 66.5)	47.1 (36.4 to 58.3)	62.8 (50.3 to 77.6)	-12 (-24.9 to 3.3)	-15.4 (-32.1 to 3.8)	-8.9 (-27.1 to 14.9)
	DALYs	Attributed all ages number	5663 (4640 to 6793)	2487 (2001 to 3005)	3175 (2429 to 4037)	9786 (8270 to 11485)	4350 (3581 to 5178)	5436 (4507 to 6478)	72.8 (46.8 to 107.3)	74.9 (42.5 to 114.3)	71.2 (33.9 to 118.7)
		Attributed age-standardized rate (per 100,000)	1293.8 (1057 to 1572.1)	1170.4 (938.8 to 1432.5)	1409.6 (1089.6 to 1801.1)	1020.8 (861 to 1208.1)	879.9 (722.3 to 1054.7)	1173.9 (968.6 to 1408.5)	-21.1 (-32.2 to -7.5)	-24.8 (-38.1 to -8.8)	-16.7 (-33.1 to 5)
	YLLs	Attributed all ages number	4945 (3957 to 6024)	2086 (1615 to 2546)	2859 (2123 to 3704)	8141 (6776 to 9731)	3462 (2781 to 4182)	4679 (3811 to 5685)	64.6 (37 to 102.1)	66 (30 to 111.6)	63.7 (25.2 to 116.5)
		Attributed age-standardized rate (per 100,000)	1155.7 (929.1 to 1415.5)	1014.2 (788.3 to 1263.2)	1290.6 (974.8 to 1676)	860.7 (714.8 to 1031.5)	710.7 (568.2 to 860.3)	1022.7 (831.6 to 1241.7)	-25.5 (-36.9 to -11.5)	-29.9 (-44 to -12.4)	-20.8 (-38.1 to 2.4)
	YLDs	Attributed all ages number	718 (511 to 948)	402 (290 to 537)	316 (222 to 421)	1645 (1191 to 2150)	889 (646 to 1161)	756 (552 to 1014)	129.1 (106.8 to 156.5)	121.3 (93.6 to 150.9)	139.1 (111.2 to 175.3)
		Attributed age-standardized rate (per 100,000)	138.1 (101.7 to 180.3)	156.3 (115.5 to 202.7)	119 (85.4 to 156.3)	160.1 (116.2 to 209.1)	169.2 (123.3 to 222)	151.2 (109.5 to 202.1)	15.9 (7.8 to 24.7)	8.3 (-1.5 to 19.3)	27.1 (15 to 40.3)

Data in parentheses are 95% Uncertainty Intervals (95% UIs).

Abbreviations: DALYs: disability-adjusted life years; YLLs: years of life lost; YLDs: years lived with disability.
