# Supplementary figures and images for: Trends of national and sub-national burden attributed to kidney dysfunction risk factor in Iran: 1990-2019
Source: Front Endocrinol (Lausanne). 2023 Feb 27;14:1115833. doi: 10.3389/fendo.2023.1115833 (PMC10010168; doi:10.3389/fendo.2023.1115833)

YLLs

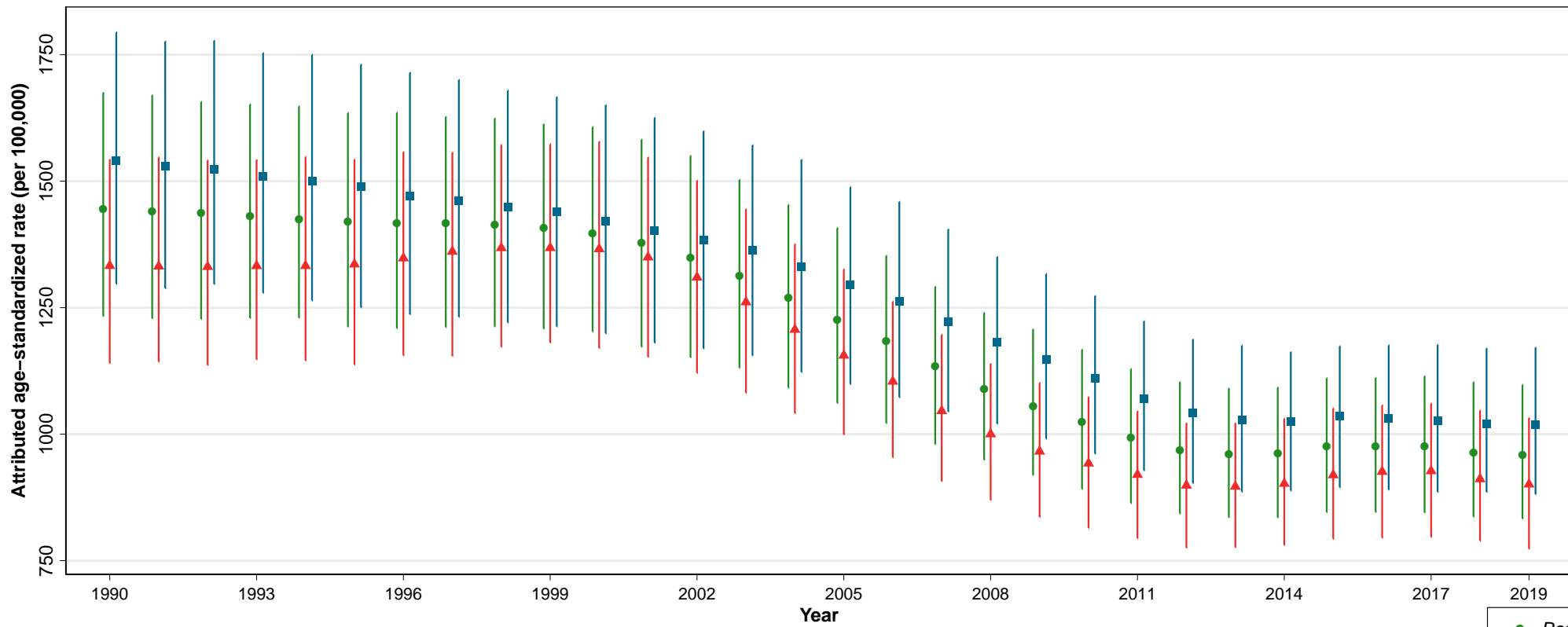

YLDs

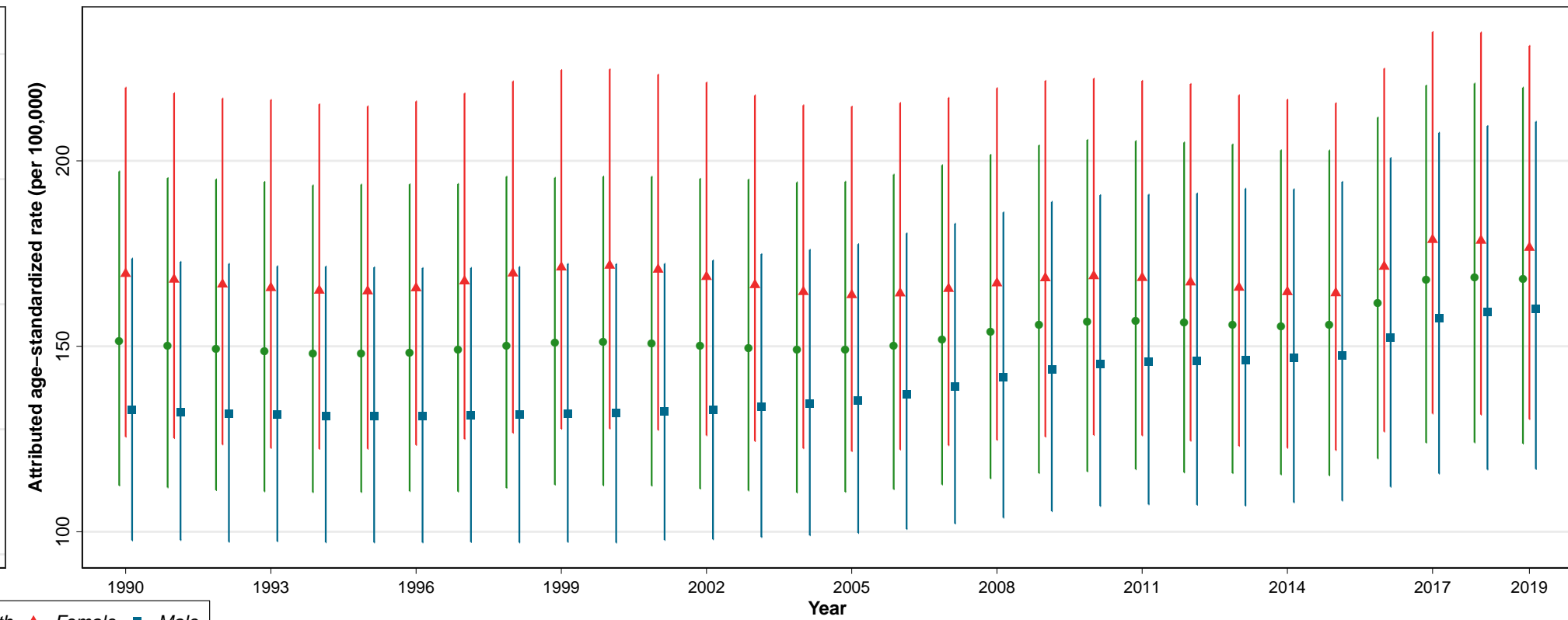

Deaths

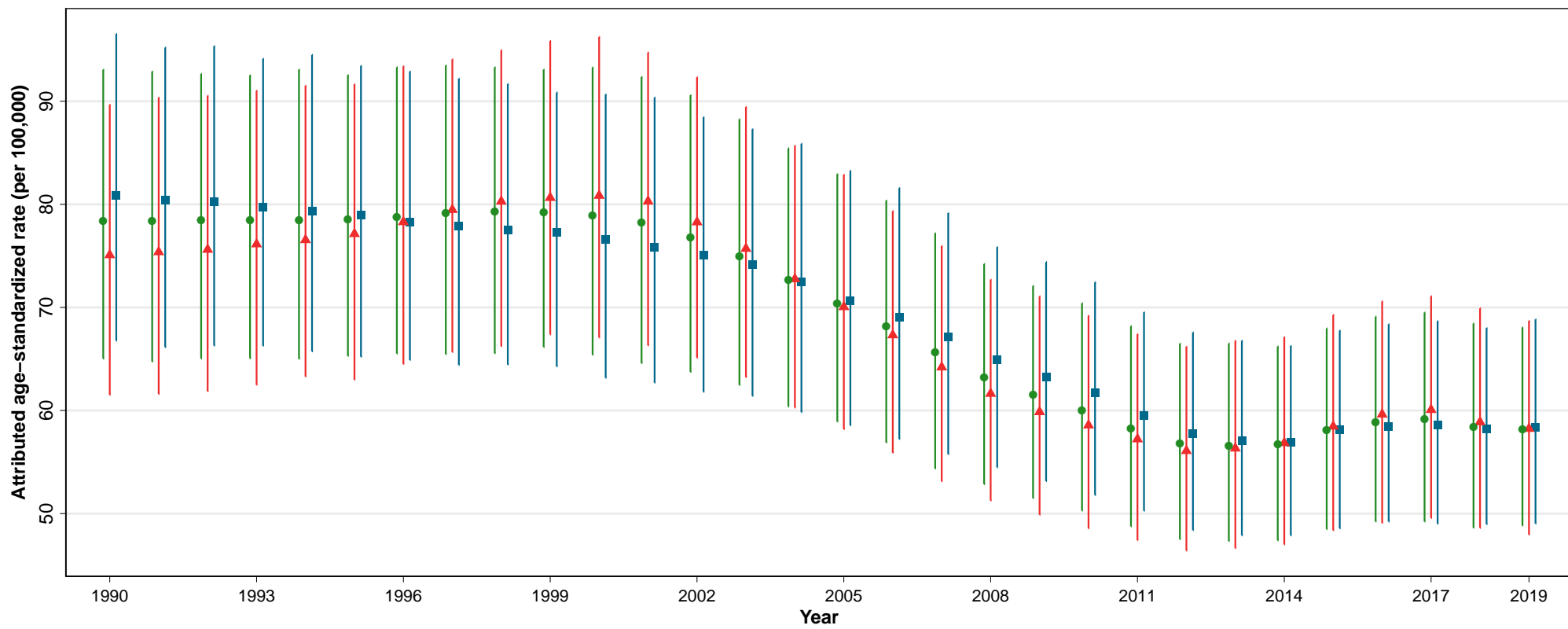

DALYs

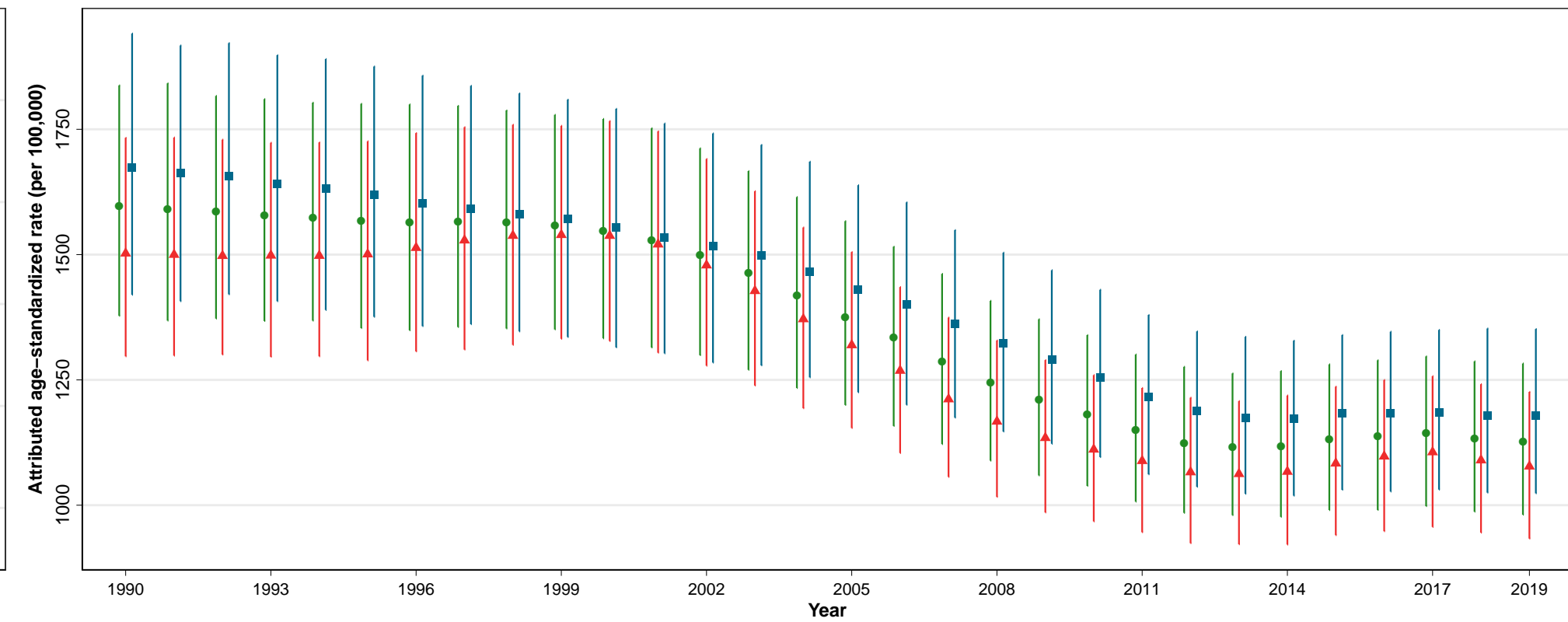

Supplement: Supplementary Figure 1 — Trend of age-standardized rate of years of life lost (YLLs), years lived with disability (YLDs), deaths, and disability-adjusted life years (DALYs) attributable to kidney dysfunction in Iran from 1990 to 2019 by sex. [file Image_1.pdf]

1990

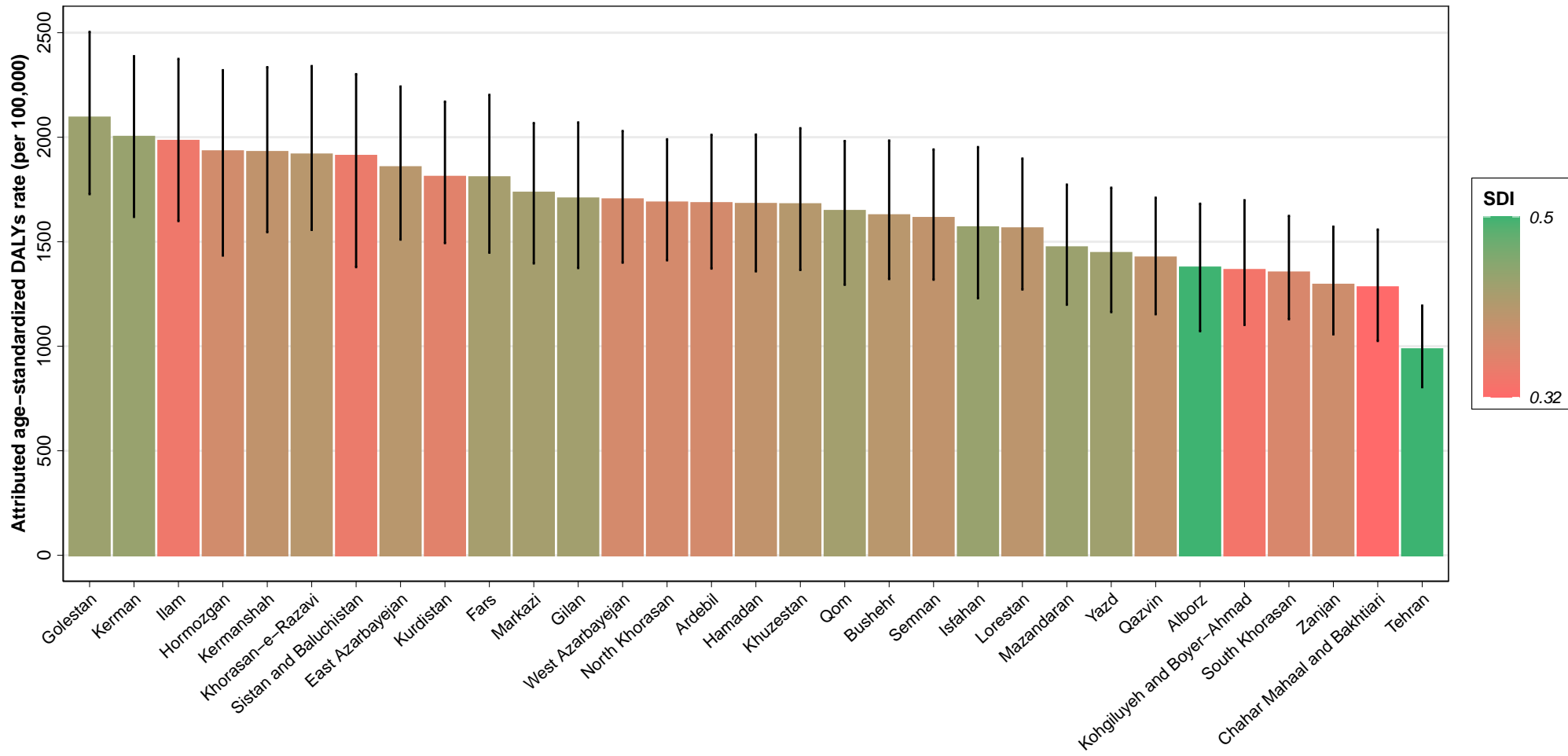

2000

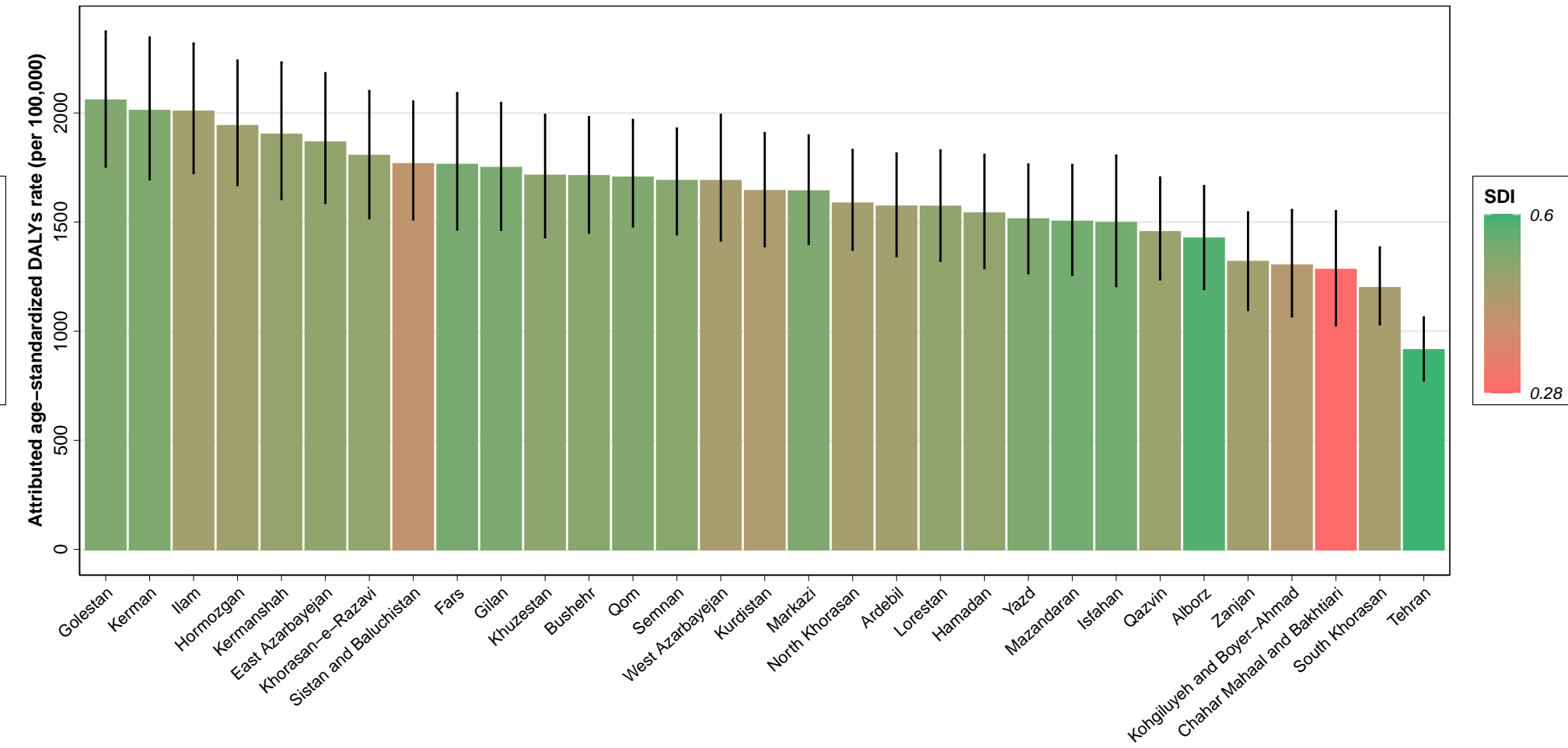

2010

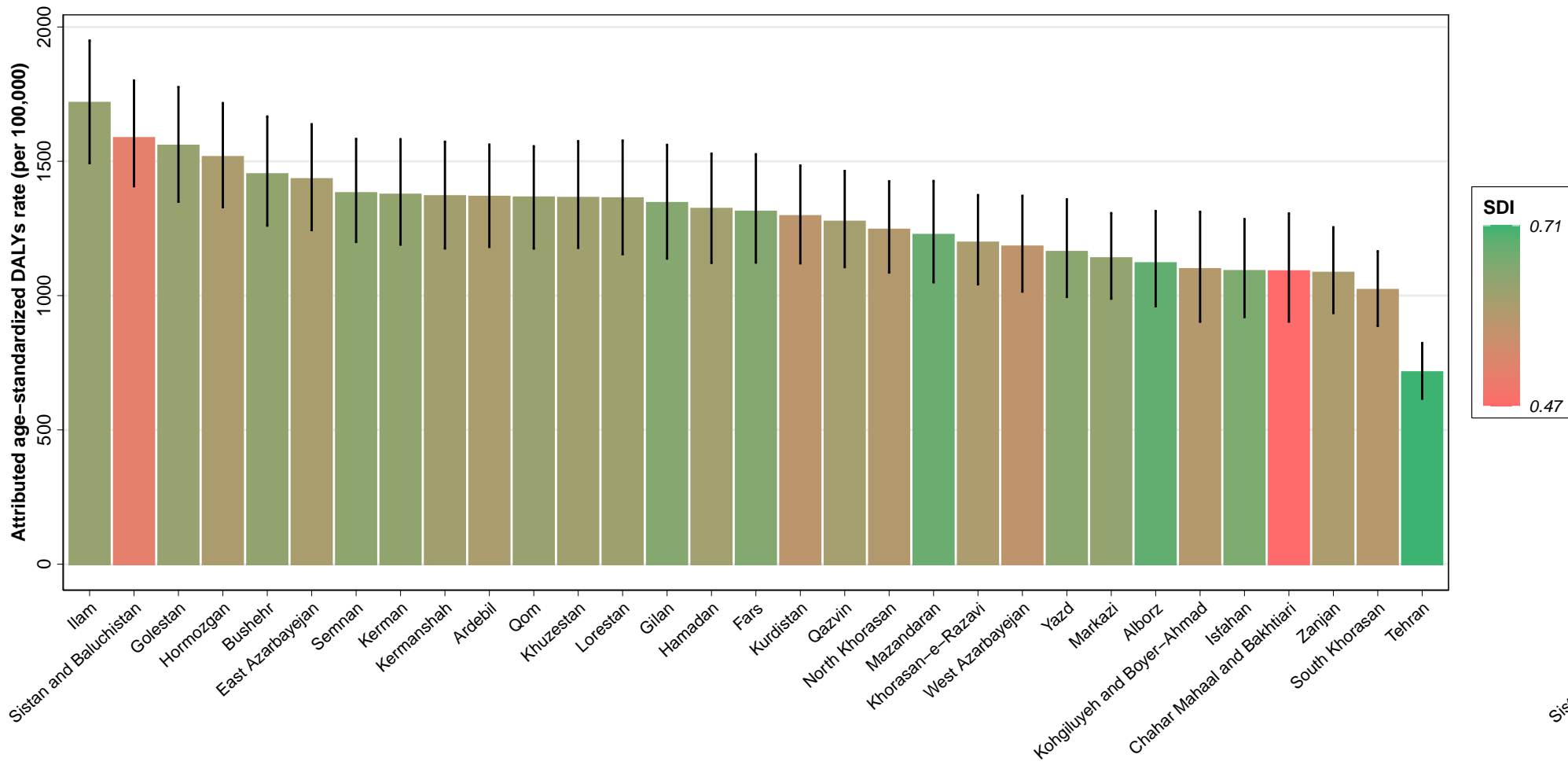

2019

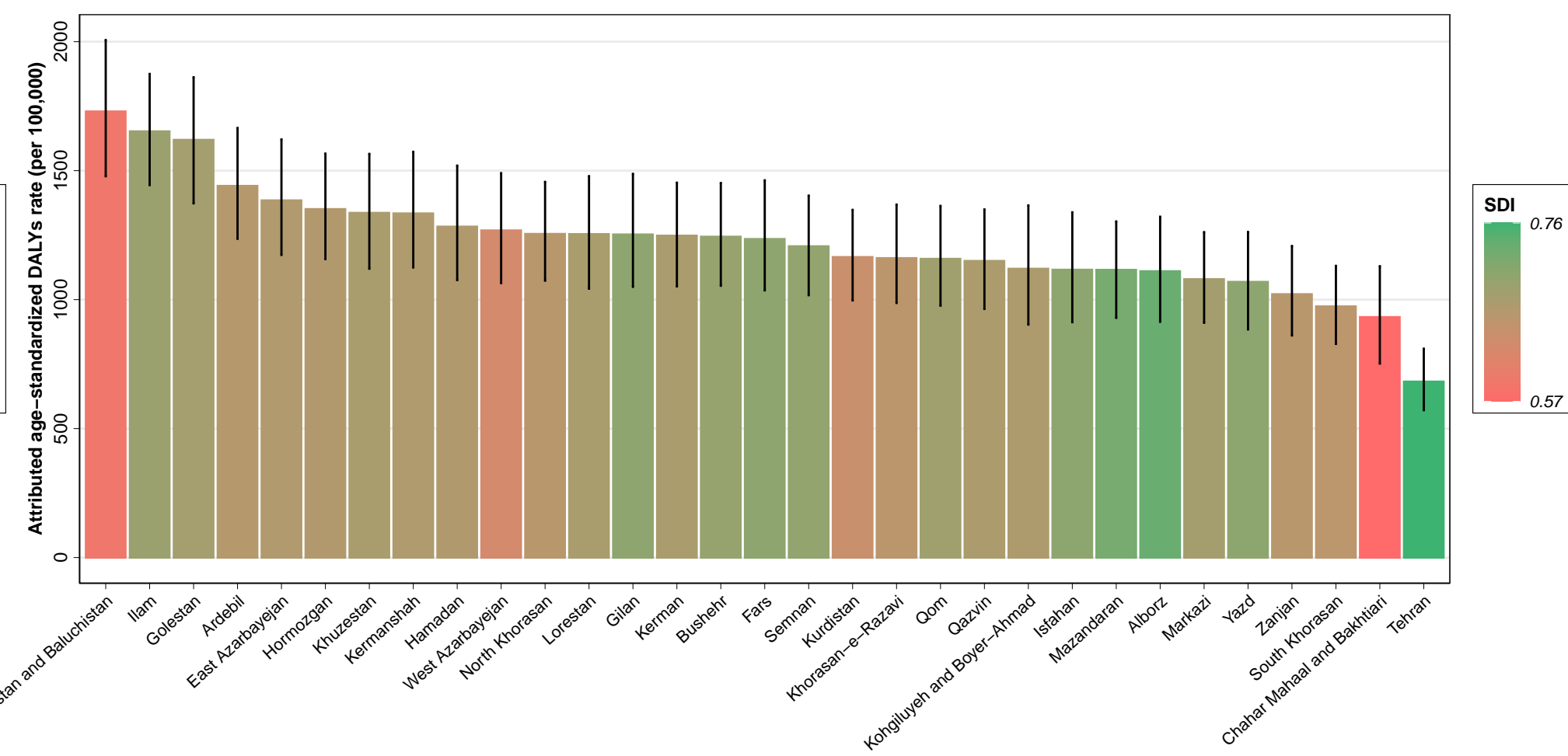

Supplement: Supplementary Figure 6 — Age-standardized rate of disability-adjusted life years (DALYs) attributable to kidney dysfunction in Iran in 1990 and 2019 by socio-demographic index (SDI) quintiles and province. [file Image_6.pdf]

1990

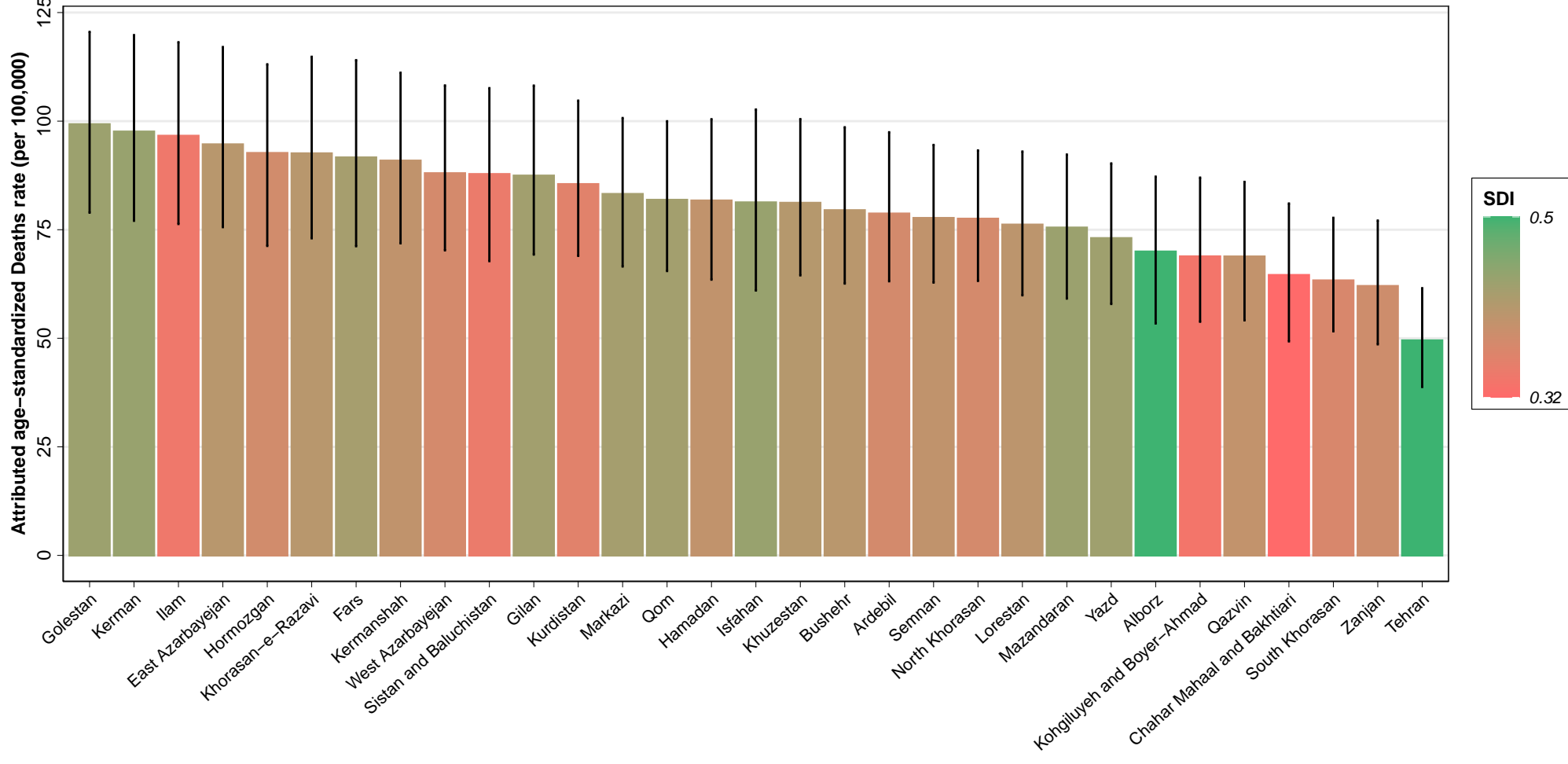

2000

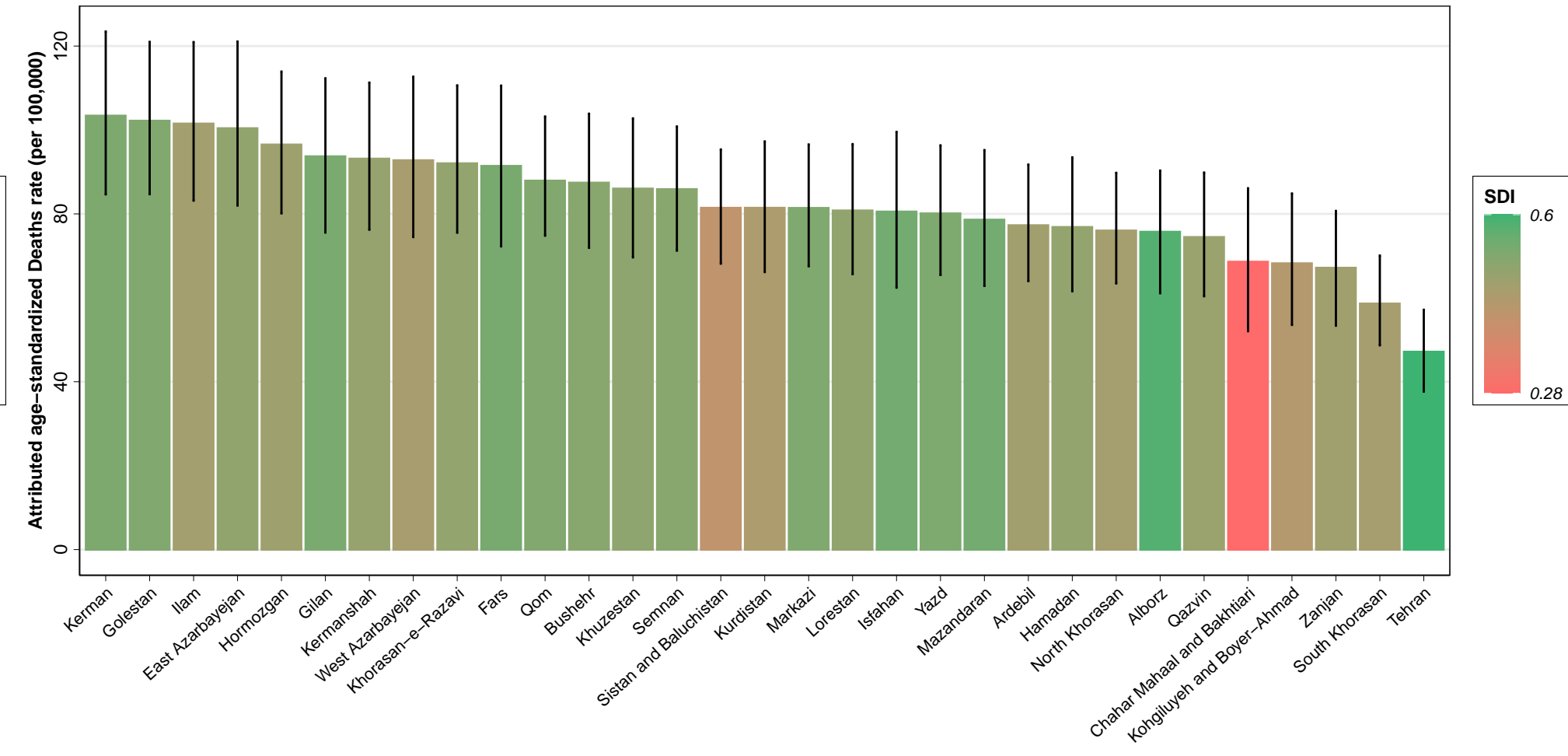

2010

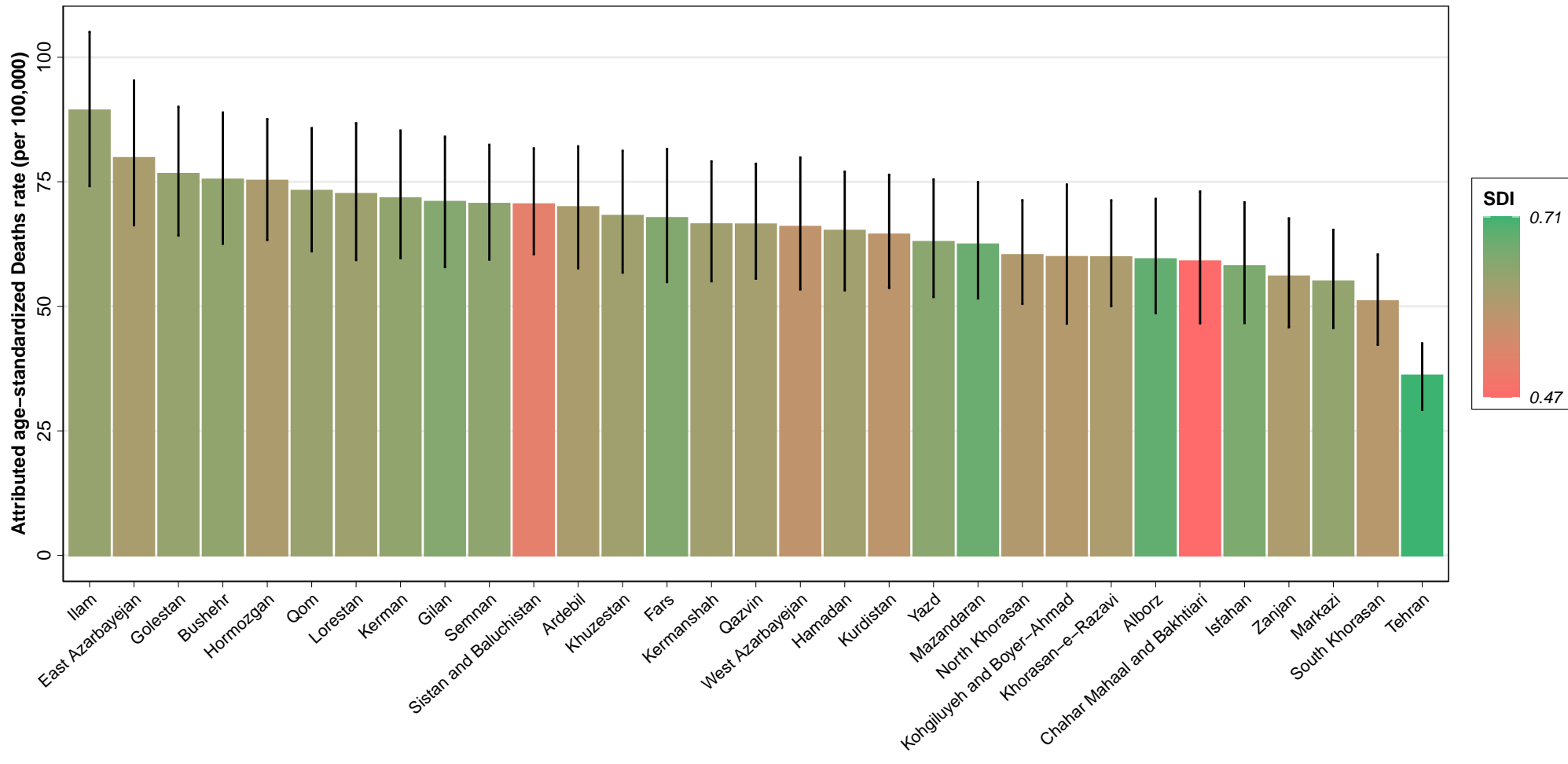

2019

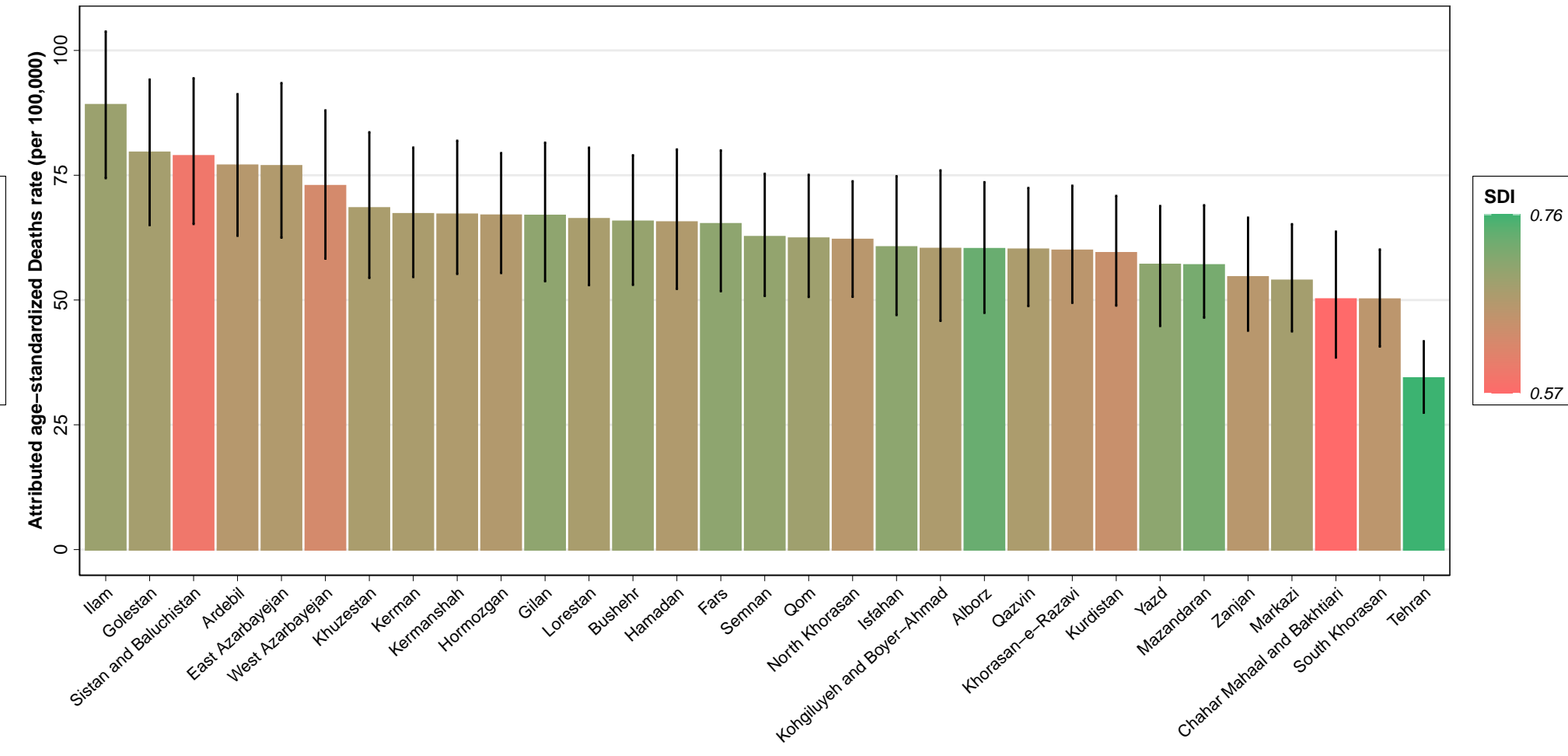

Supplement: Supplementary Figure 7 — Age-standardized rate of deaths attributable to kidney dysfunction in Iran in 1990 and 2019 by socio-demographic index (SDI) quintiles and province. [file Image_7.pdf]
