# Supplementary material for: Trends of national and sub-national burden attributed to kidney dysfunction risk factor in Iran: 1990-2019
Source: Front Endocrinol (Lausanne). 2023 Feb 27;14:1115833. doi: 10.3389/fendo.2023.1115833 (PMC10010168; doi:10.3389/fendo.2023.1115833)
Supplement: Supplementary Figure 2 — Age-standardized rate of years of life lost (YLLs), years lived with disability (YLDs), deaths, and disability-adjusted life years (DALYs) attributable to kidney dysfunction among both sexes in Iran in 1990 by province. [file Image_2.pdf]

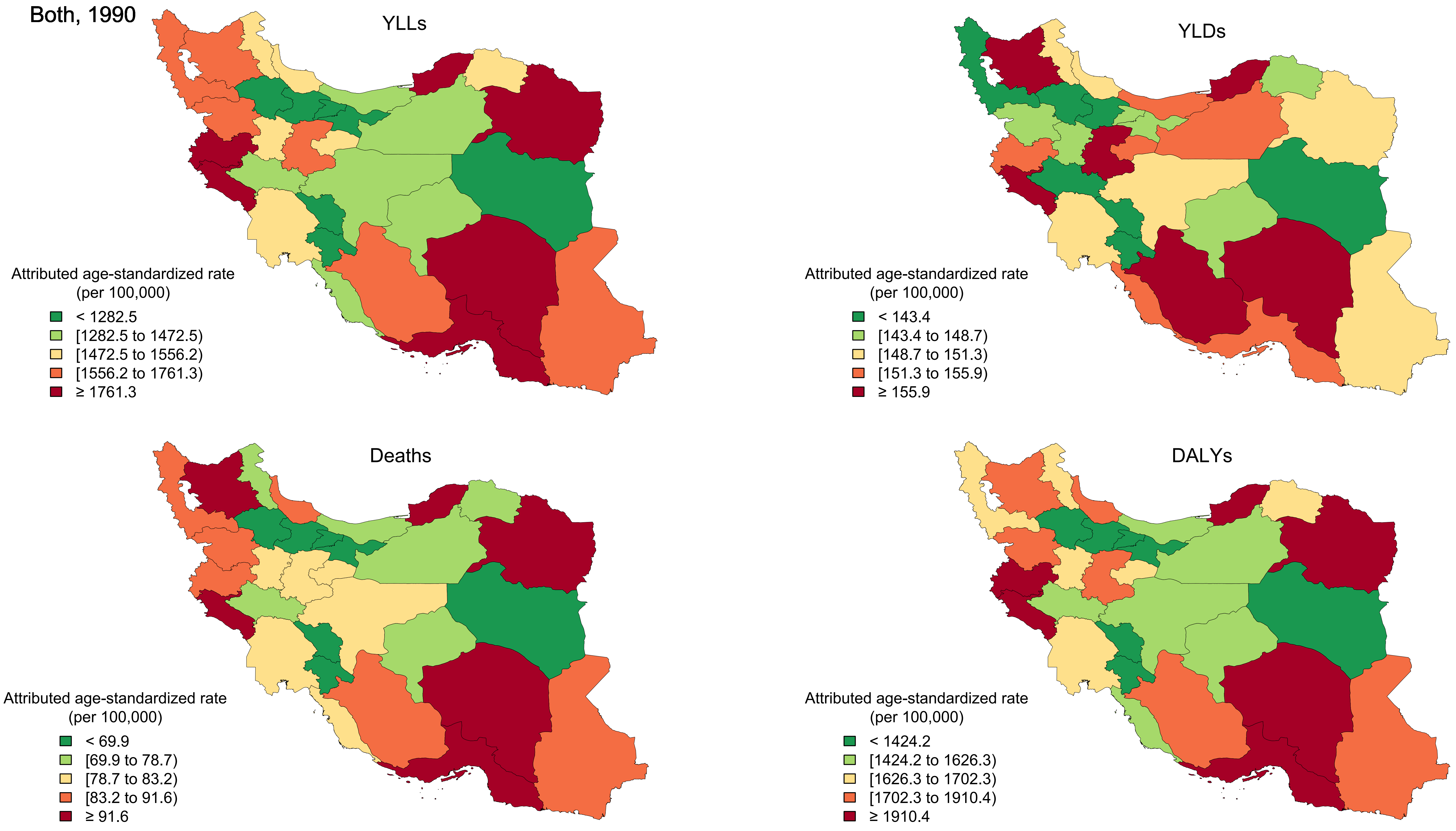
Both, 1990
YLLs
Attributed age-standardized rate
(per 100,000)
< 1282.5
[1282.5 to 1472.5)
[1472.5 to 1556.2)
[1556.2 to 1761.3)
≥ 1761.3
YLDs
Attributed age-standardized rate
(per 100,000)
< 143.4
[143.4 to 148.7)
[148.7 to 151.3)
[151.3 to 155.9)
≥ 155.9
Deaths
Attributed age-standardized rate
(per 100,000)
< 69.9
[69.9 to 78.7)
[78.7 to 83.2)
[83.2 to 91.6)
≥ 91.6
DALYs
Attributed age-standardized rate
(per 100,000)
< 1424.2
[1424.2 to 1626.3)
[1626.3 to 1702.3)
[1702.3 to 1910.4)
≥ 1910.4
