# Supplementary material for: Trends of national and sub-national burden attributed to kidney dysfunction risk factor in Iran: 1990-2019
Source: Front Endocrinol (Lausanne). 2023 Feb 27;14:1115833. doi: 10.3389/fendo.2023.1115833 (PMC10010168; doi:10.3389/fendo.2023.1115833)
Supplement: Supplementary Figure 3 — Age-standardized rate of years of life lost (YLLs), years lived with disability (YLDs), deaths, and disability-adjusted life years (DALYs) attributable to kidney dysfunction among both sexes in Iran in 2019 by province. [file Image_3.pdf]

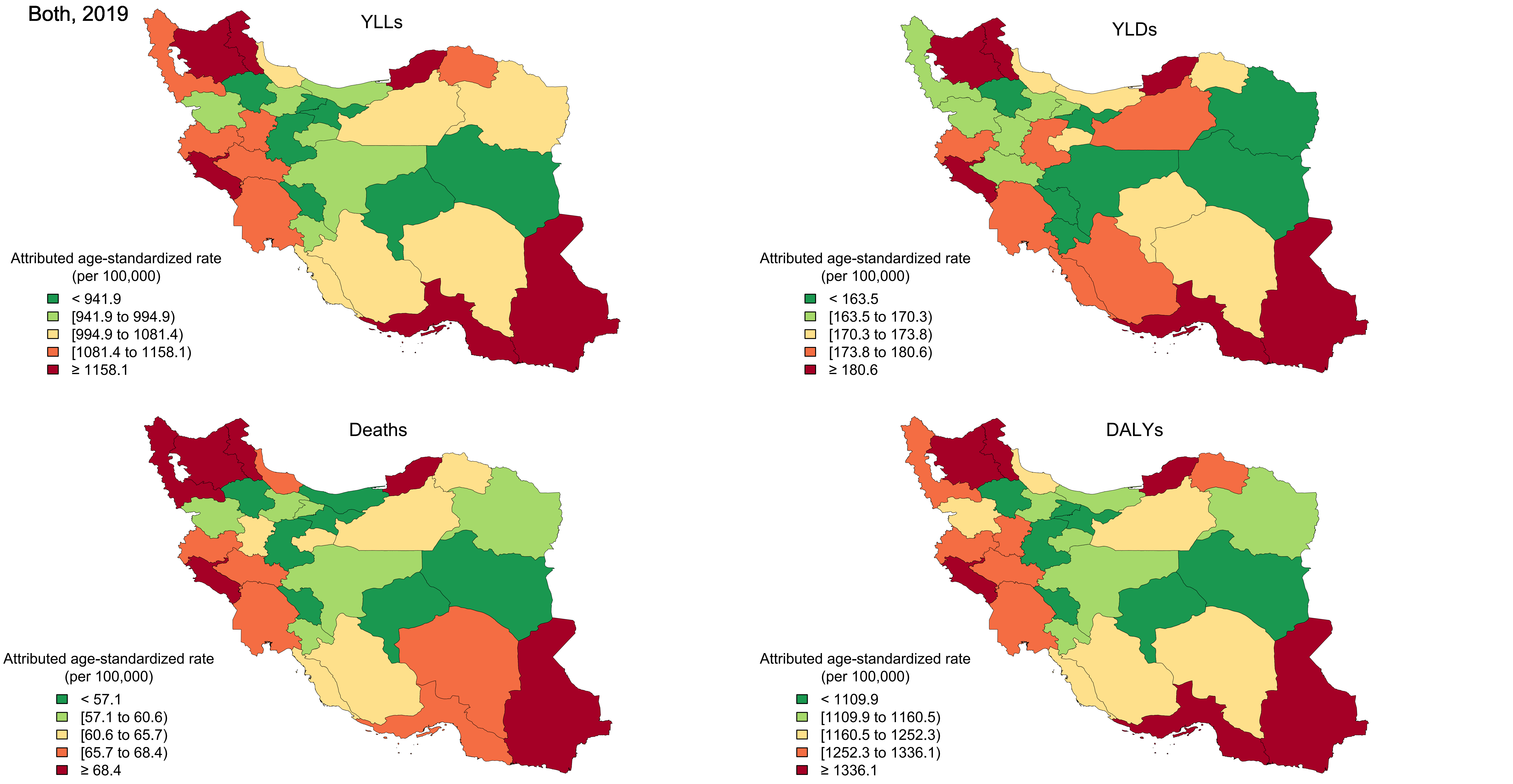

Both, 2019
YLLs
Attributed age-standardized rate
(per 100,000)
< 941.9
[941.9 to 994.9)
[994.9 to 1081.4)
[1081.4 to 1158.1)
≥ 1158.1
YLDs
Attributed age-standardized rate
(per 100,000)
< 163.5
[163.5 to 170.3)
[170.3 to 173.8)
[173.8 to 180.6)
≥ 180.6
Deaths
Attributed age-standardized rate
(per 100,000)
< 57.1
[57.1 to 60.6)
[60.6 to 65.7)
[65.7 to 68.4)
≥ 68.4
DALYs
Attributed age-standardized rate
(per 100,000)
< 1109.9
[1109.9 to 1160.5)
[1160.5 to 1252.3)
[1252.3 to 1336.1)
≥ 1336.1
