# Supplementary material for: Trends of national and sub-national burden attributed to kidney dysfunction risk factor in Iran: 1990-2019
Source: Front Endocrinol (Lausanne). 2023 Feb 27;14:1115833. doi: 10.3389/fendo.2023.1115833 (PMC10010168; doi:10.3389/fendo.2023.1115833)
Supplement: Supplementary Figure 4 — Trend of age-standardized rate of years of life lost (YLLs), years lived with disability (YLDs), deaths, and disability-adjusted life years (DALYs) attributable to kidney dysfunction in Iran from 1990 to 2019 by province. [file Image_4.pdf]

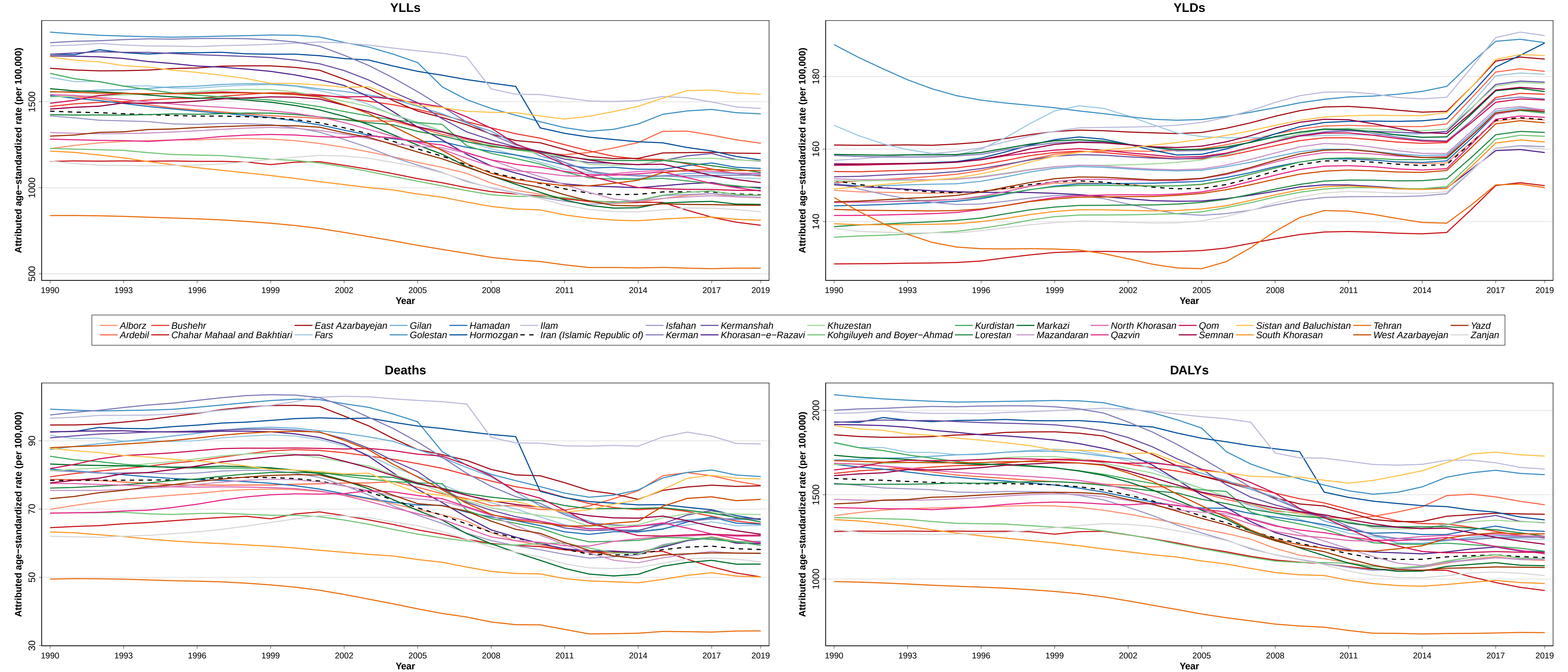
YLLs
YLDs
Deaths
DALYs
Attributed age−standardized rate (per 100,000)
Year
1990
1993
1996
1999
2002
2005
2008
2011
2014
2017
2019
500
1000
1500
140
160
180
30
50
70
90
2000
Alborz
Ardebil
Bushehr
Chahar Mahaal and Bakhtiari
East Azarbayejan
Fars
Gilan
Golestan
Hamadan
Hormozgan
Ilam
Iran (Islamic Republic of)
Isfahan
Kerman
Kermanshah
Khorasan−e−Razavi
Khuzestan
Kohgiluyeh and Boyer−Ahmad
Kurdistan
Lorestan
Markazi
Mazandaran
North Khorasan
Qazvin
Qom
Semnan
Sistan and Baluchistan
South Khorasan
Tehran
West Azarbayejan
Yazd
Zanjan
