# Supplementary material for: Trends of national and sub-national burden attributed to kidney dysfunction risk factor in Iran: 1990-2019
Source: Front Endocrinol (Lausanne). 2023 Feb 27;14:1115833. doi: 10.3389/fendo.2023.1115833 (PMC10010168; doi:10.3389/fendo.2023.1115833)
Supplement: Supplementary Figure 5 — Age-standardized rate of years of life lost (YLLs), years lived with disability (YLDs), deaths and disability-adjusted life years (DALYs) attributable to kidney dysfunction in provinces of Iran in 1990 and 2019 by sex and age. [file Image_5.pdf]

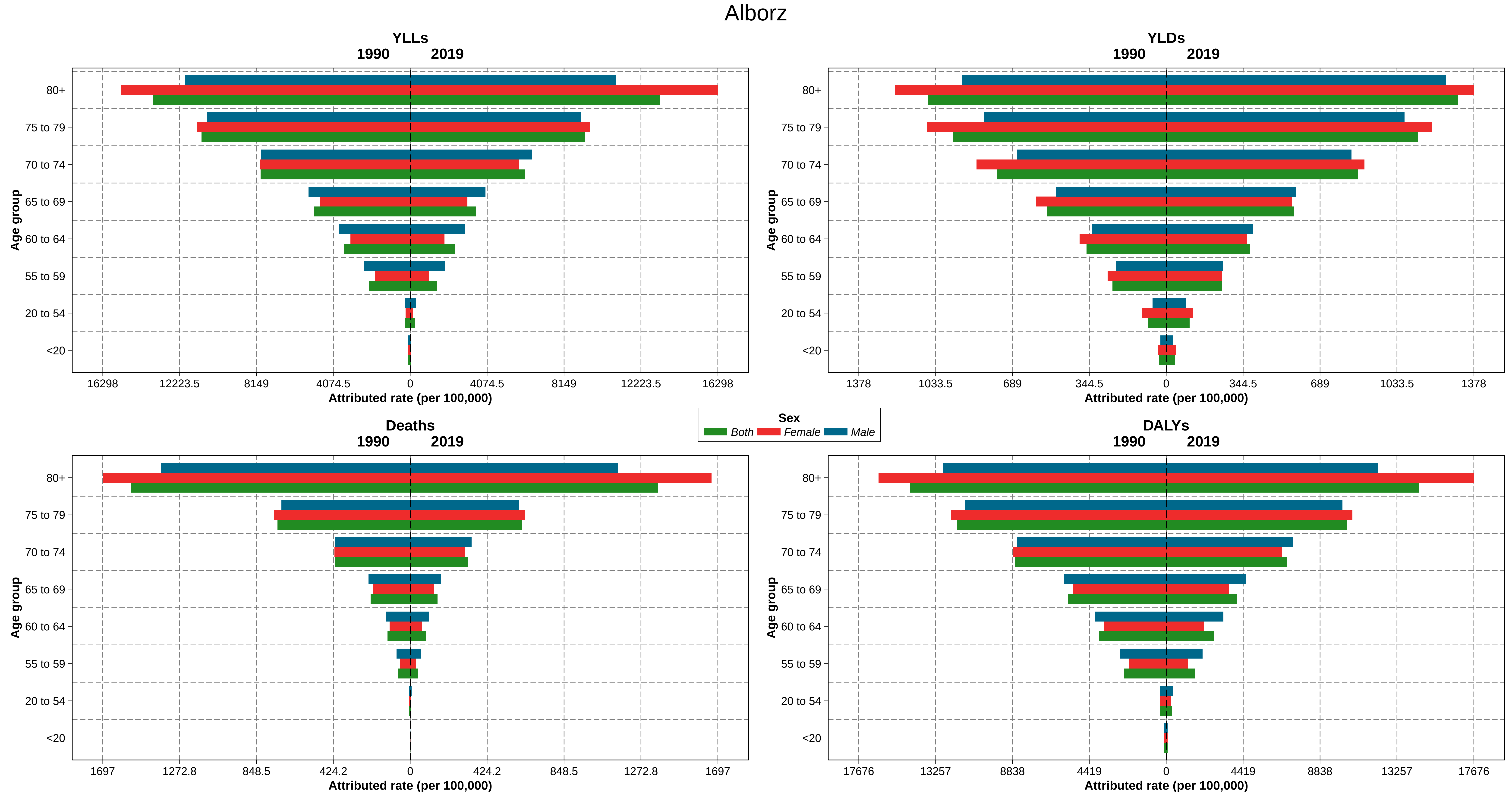
Alborz
YLLs
1990
2019
80+
75 to 79
70 to 74
65 to 69
60 to 64
55 to 59
20 to 54
<20
Age group
16298
12223.5
8149
4074.5
0
4074.5
8149
12223.5
16298
Attributed rate (per 100,000)
YLDs
1990
2019
1378
1033.5
689
344.5
0
344.5
689
1033.5
1378
Attributed rate (per 100,000)
Sex
Both
Female
Male
Deaths
1990
2019
1697
1272.8
848.5
424.2
0
424.2
848.5
1272.8
1697
Attributed rate (per 100,000)
DALYs
1990
2019
17676
13257
8838
4419
0
4419
8838
13257
17676
Attributed rate (per 100,000)

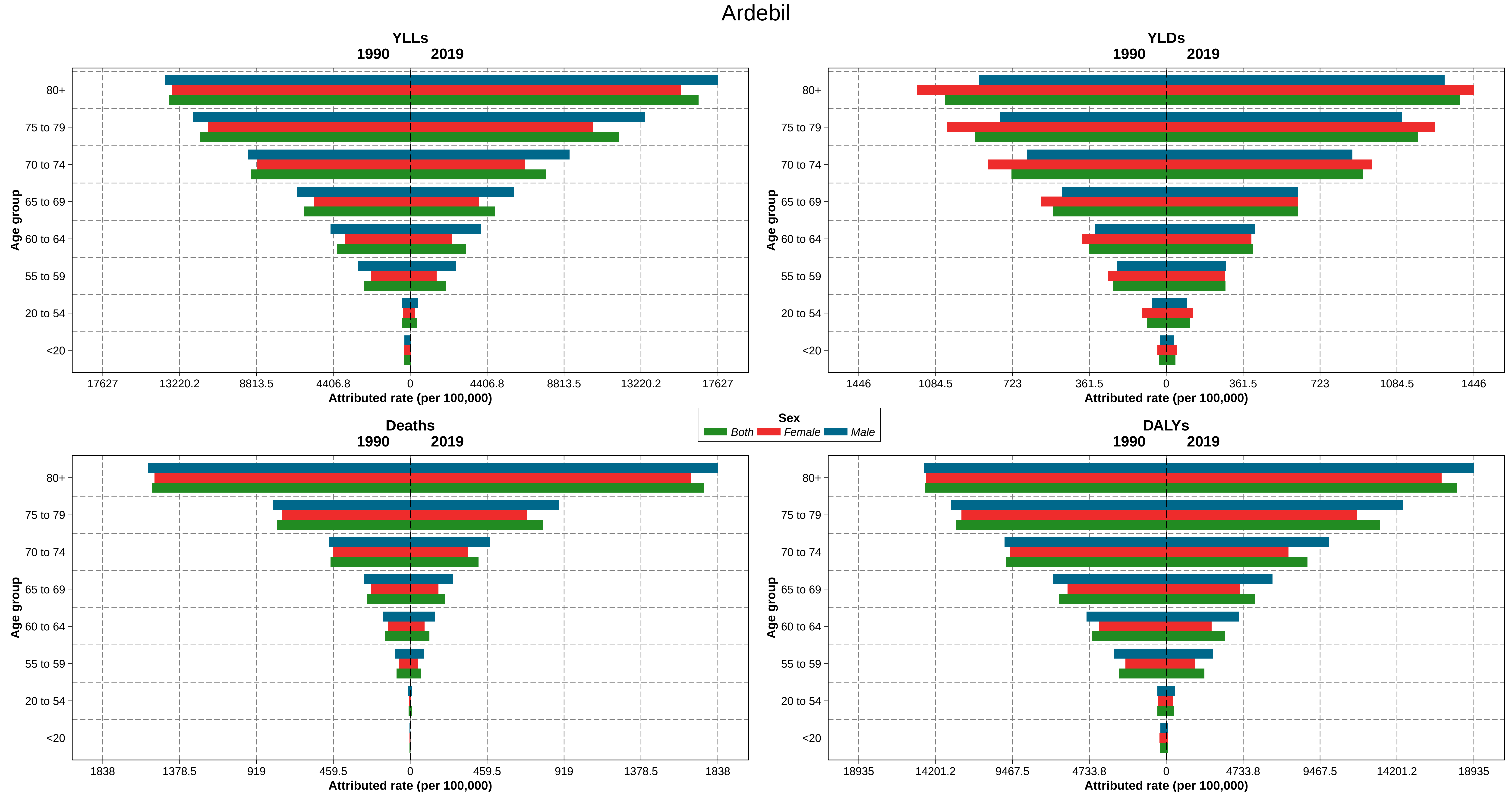

Ardebil
YLLs
1990
2019
YLDs
1990
2019
Deaths
1990
2019
DALYs
1990
2019
Age group
80+
75 to 79
70 to 74
65 to 69
60 to 64
55 to 59
20 to 54
<20
17627
13220.2
8813.5
4406.8
0
4406.8
8813.5
13220.2
17627
1446
1084.5
723
361.5
0
361.5
723
1084.5
1446
1838
1378.5
919
459.5
0
459.5
919
1378.5
1838
18935
14201.2
9467.5
4733.8
0
4733.8
9467.5
14201.2
18935
Attributed rate (per 100,000)
Sex
Both
Female
Male

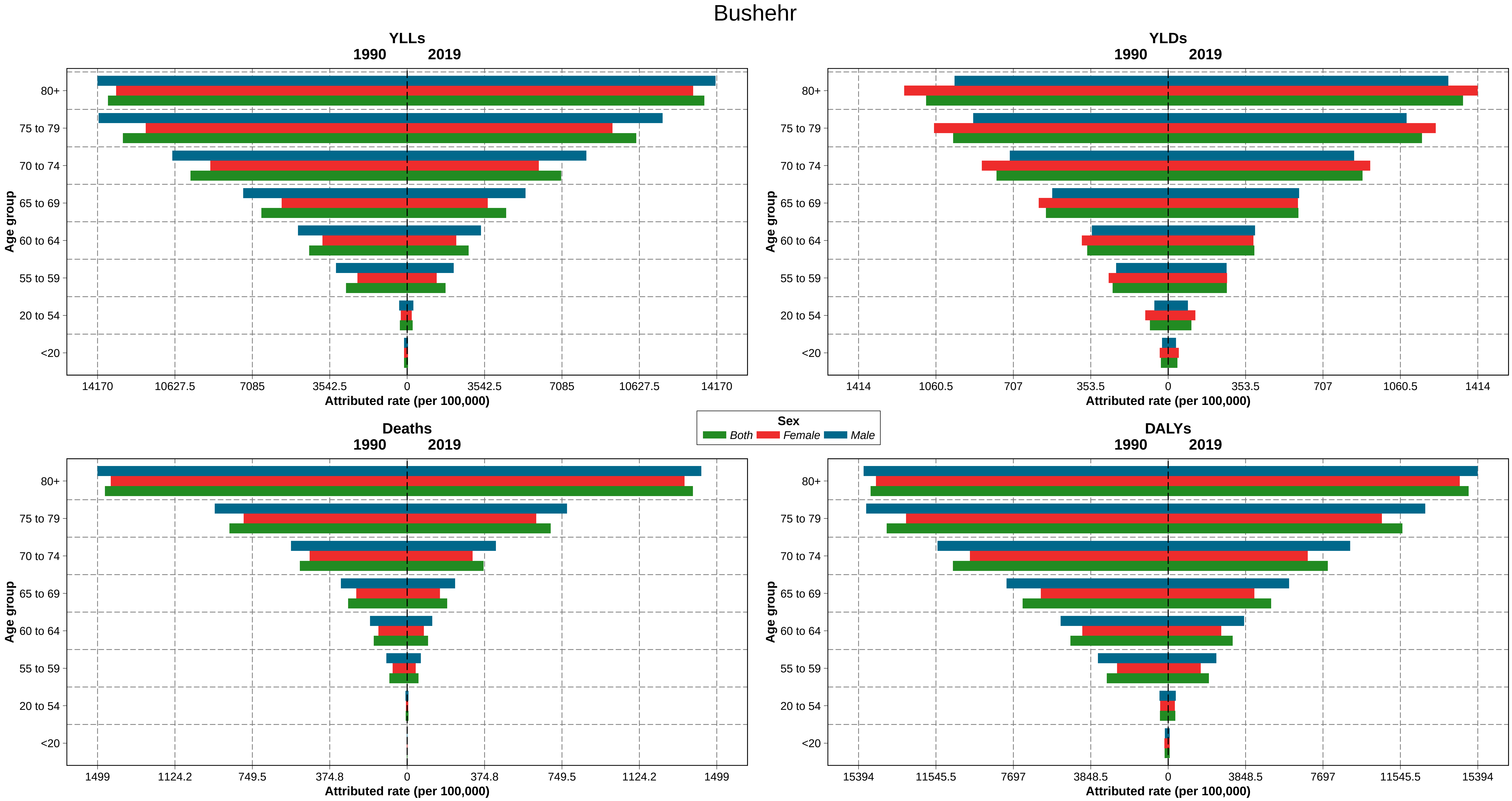
Bushehr
YLLs
1990
2019
YLDs
1990
2019
Deaths
1990
2019
DALYs
1990
2019
Age group
80+
75 to 79
70 to 74
65 to 69
60 to 64
55 to 59
20 to 54
<20
14170
10627.5
7085
3542.5
0
3542.5
7085
10627.5
14170
1414
1060.5
707
353.5
0
353.5
707
1060.5
1414
1499
1124.2
749.5
374.8
0
374.8
749.5
1124.2
1499
15394
11545.5
7697
3848.5
0
3848.5
7697
11545.5
15394
Attributed rate (per 100,000)
Sex
Both
Female
Male

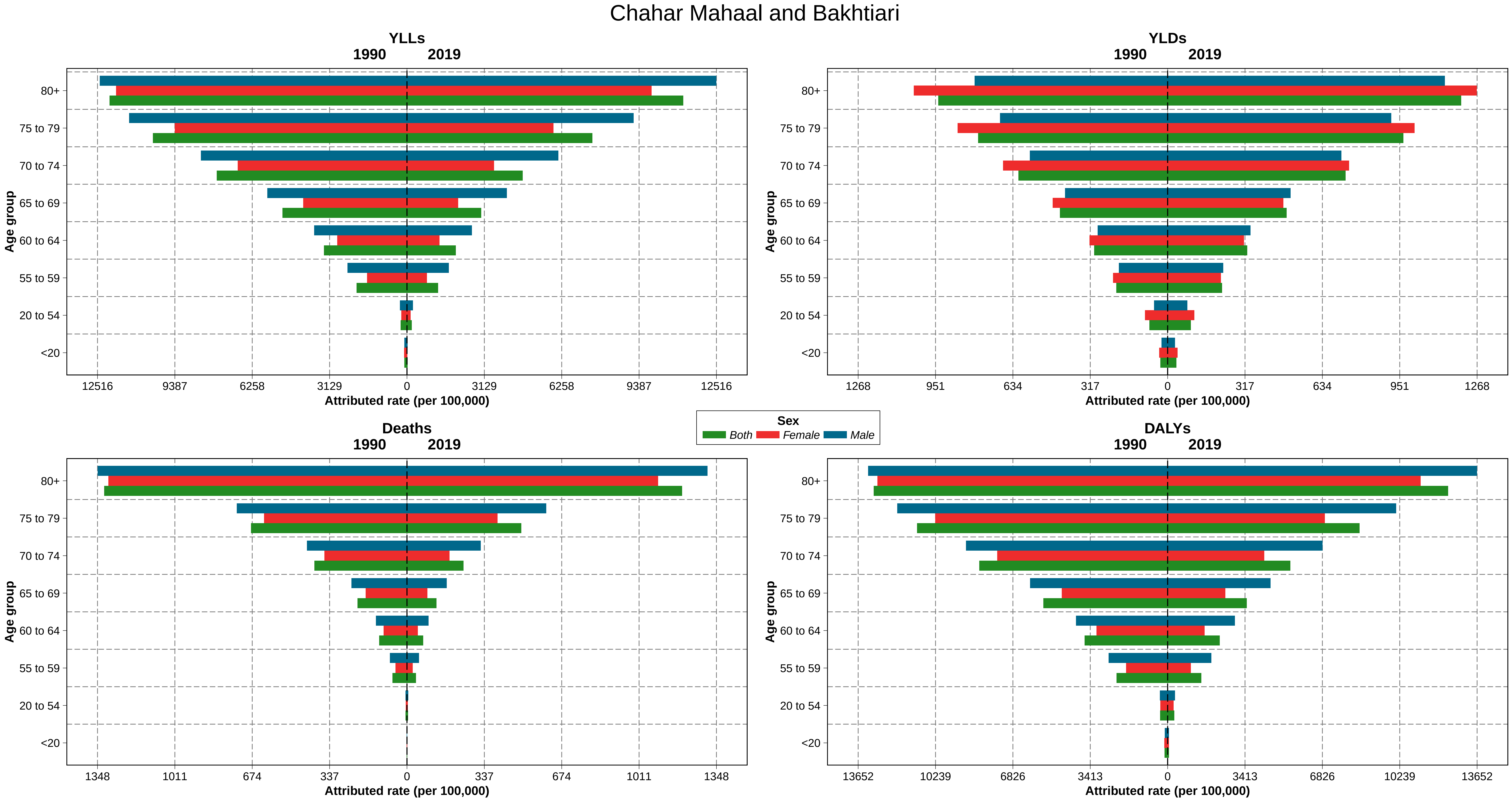

Chahar Mahaal and Bakhtiari
YLLs
1990
2019
YLDs
1990
2019
Deaths
1990
2019
DALYs
1990
2019
Age group
80+
75 to 79
70 to 74
65 to 69
60 to 64
55 to 59
20 to 54
<20
12516 9387 6258 3129 0 3129 6258 9387 12516
1268 951 634 317 0 317 634 951 1268
1348 1011 674 337 0 337 674 1011 1348
13652 10239 6826 3413 0 3413 6826 10239 13652
Attributed rate (per 100,000)
Sex
Both
Female
Male

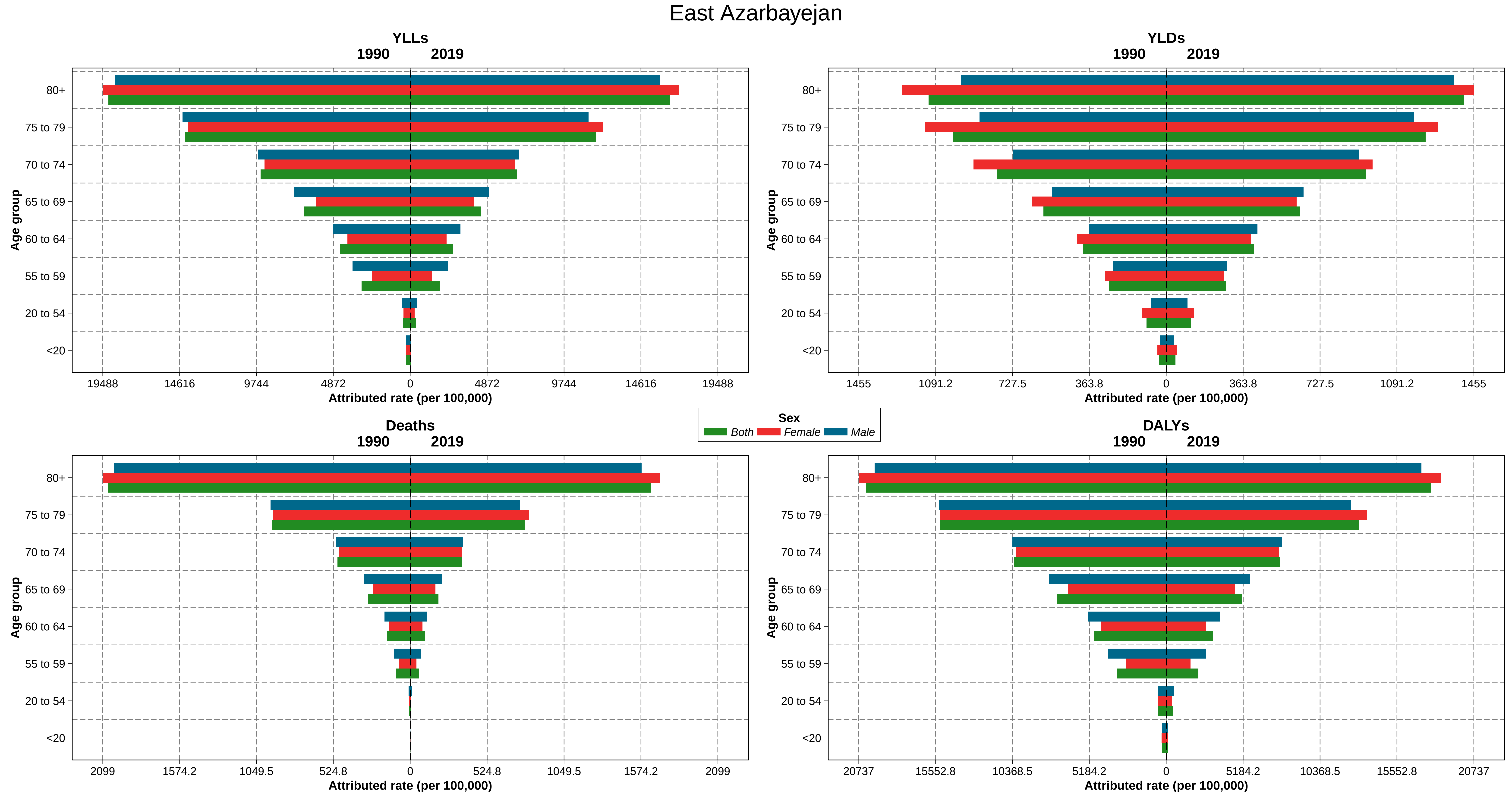

East Azarbayejan
YLLs
1990
2019
80+
75 to 79
70 to 74
65 to 69
60 to 64
55 to 59
20 to 54
<20
Age group
19488
14616
9744
4872
0
4872
9744
14616
19488
Attributed rate (per 100,000)
YLDs
1990
2019
1455
1091.2
727.5
363.8
0
363.8
727.5
1091.2
1455
Attributed rate (per 100,000)
Sex
Both
Female
Male
Deaths
1990
2019
2099
1574.2
1049.5
524.8
0
524.8
1049.5
1574.2
2099
Attributed rate (per 100,000)
DALYs
1990
2019
20737
15552.8
10368.5
5184.2
0
5184.2
10368.5
15552.8
20737
Attributed rate (per 100,000)

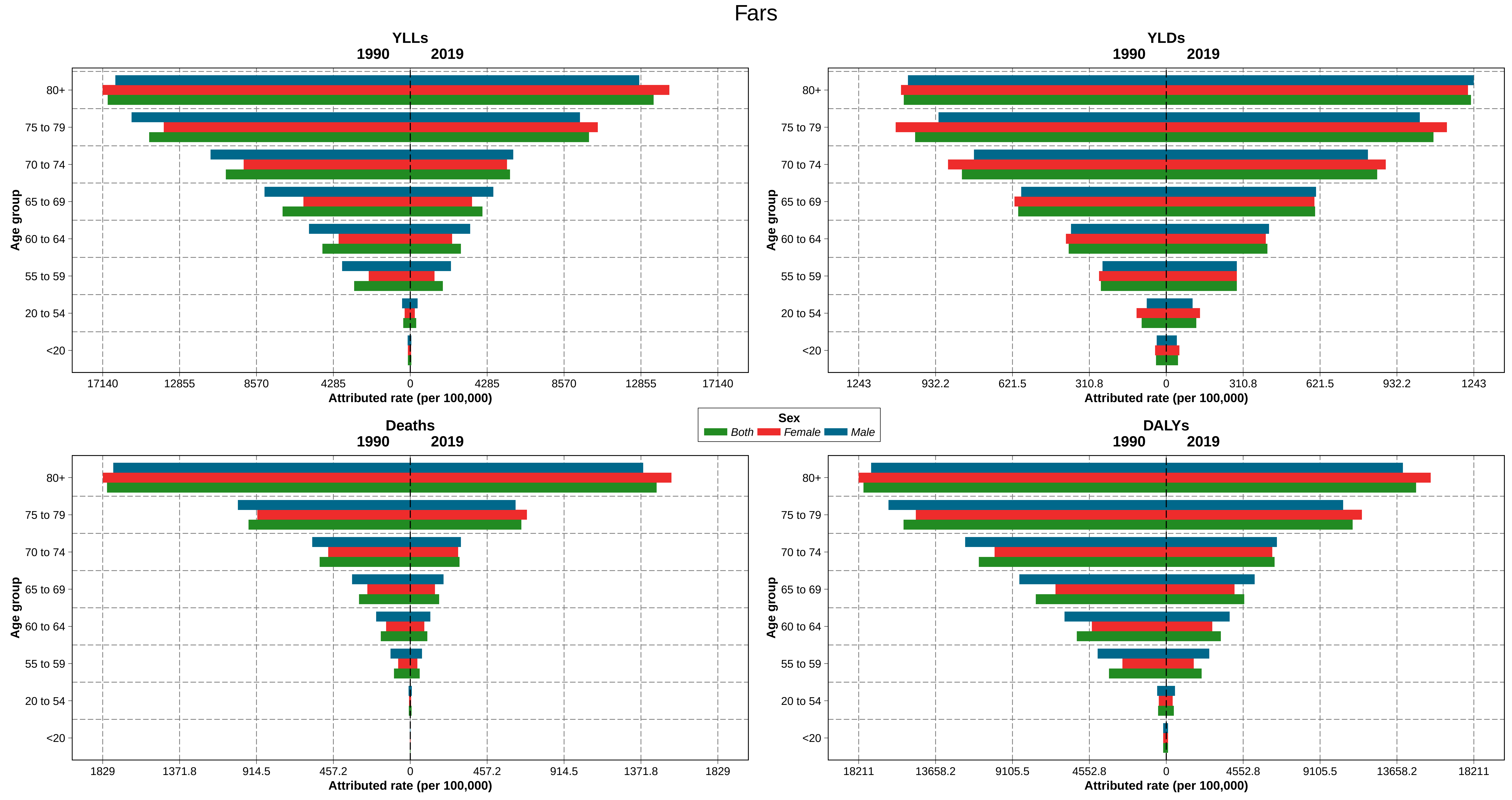

Fars
YLLs
1990
2019
YLDs
1990
2019
Deaths
1990
2019
DALYs
1990
2019
Age group
80+
75 to 79
70 to 74
65 to 69
60 to 64
55 to 59
20 to 54
<20
17140
12855
8570
4285
0
4285
8570
12855
17140
1243
932.2
621.5
310.8
0
310.8
621.5
932.2
1243
1829
1371.8
914.5
457.2
0
457.2
914.5
1371.8
1829
18211
13658.2
9105.5
4552.8
0
4552.8
9105.5
13658.2
18211
Attributed rate (per 100,000)
Sex
Both
Female
Male

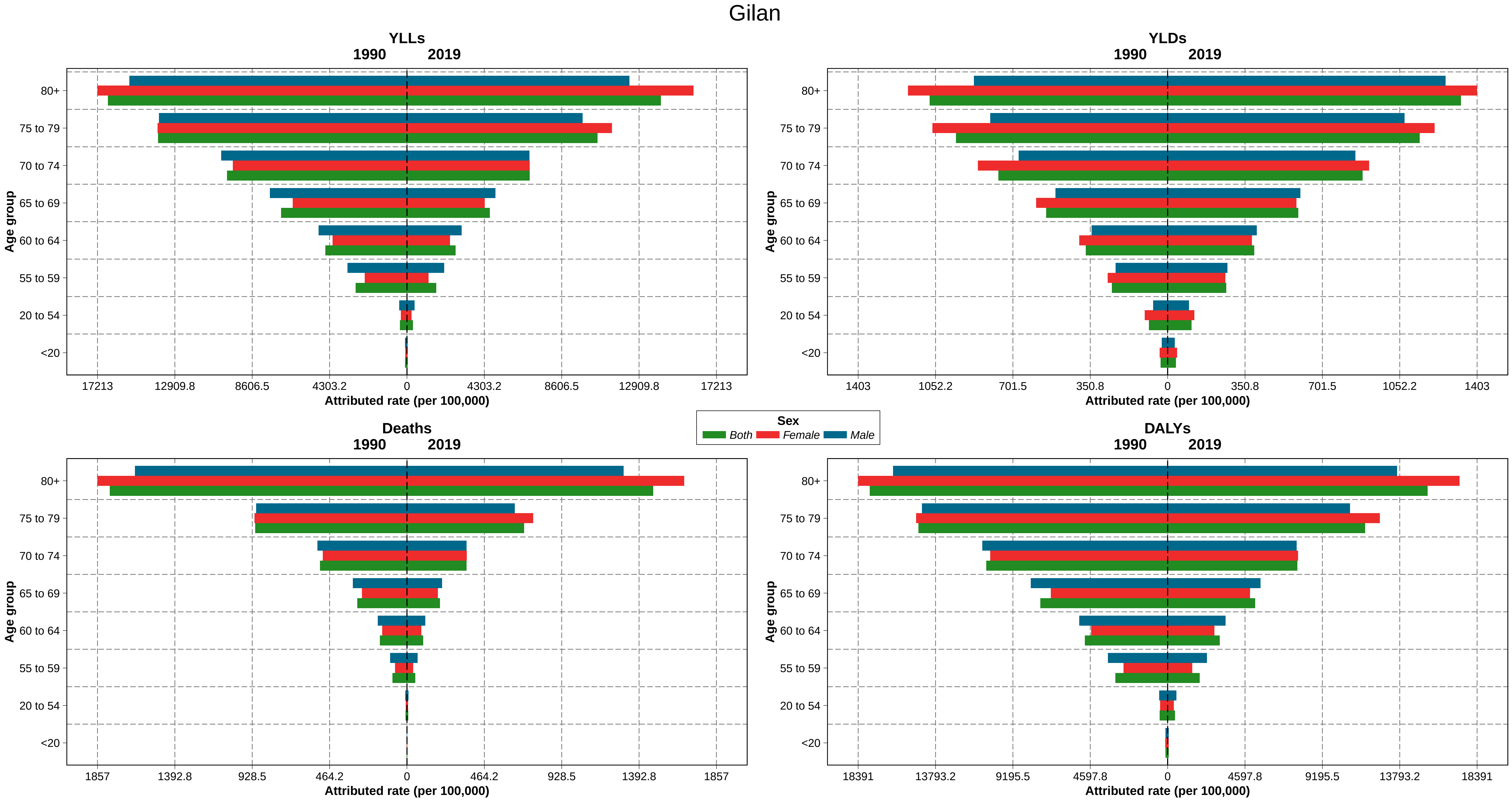
Gilan
YLLs
1990 2019
YLDs
1990 2019
Deaths
1990 2019
DALYs
1990 2019
Age group
80+
75 to 79
70 to 74
65 to 69
60 to 64
55 to 59
20 to 54
<20
17213 12909.8 8606.5 4303.2 0 4303.2 8606.5 12909.8 17213
1403 1052.2 701.5 350.8 0 350.8 701.5 1052.2 1403
1857 1392.8 928.5 464.2 0 464.2 928.5 1392.8 1857
18391 13793.2 9195.5 4597.8 0 4597.8 9195.5 13793.2 18391
Attributed rate (per 100,000)
Sex
Both
Female
Male

Golestan

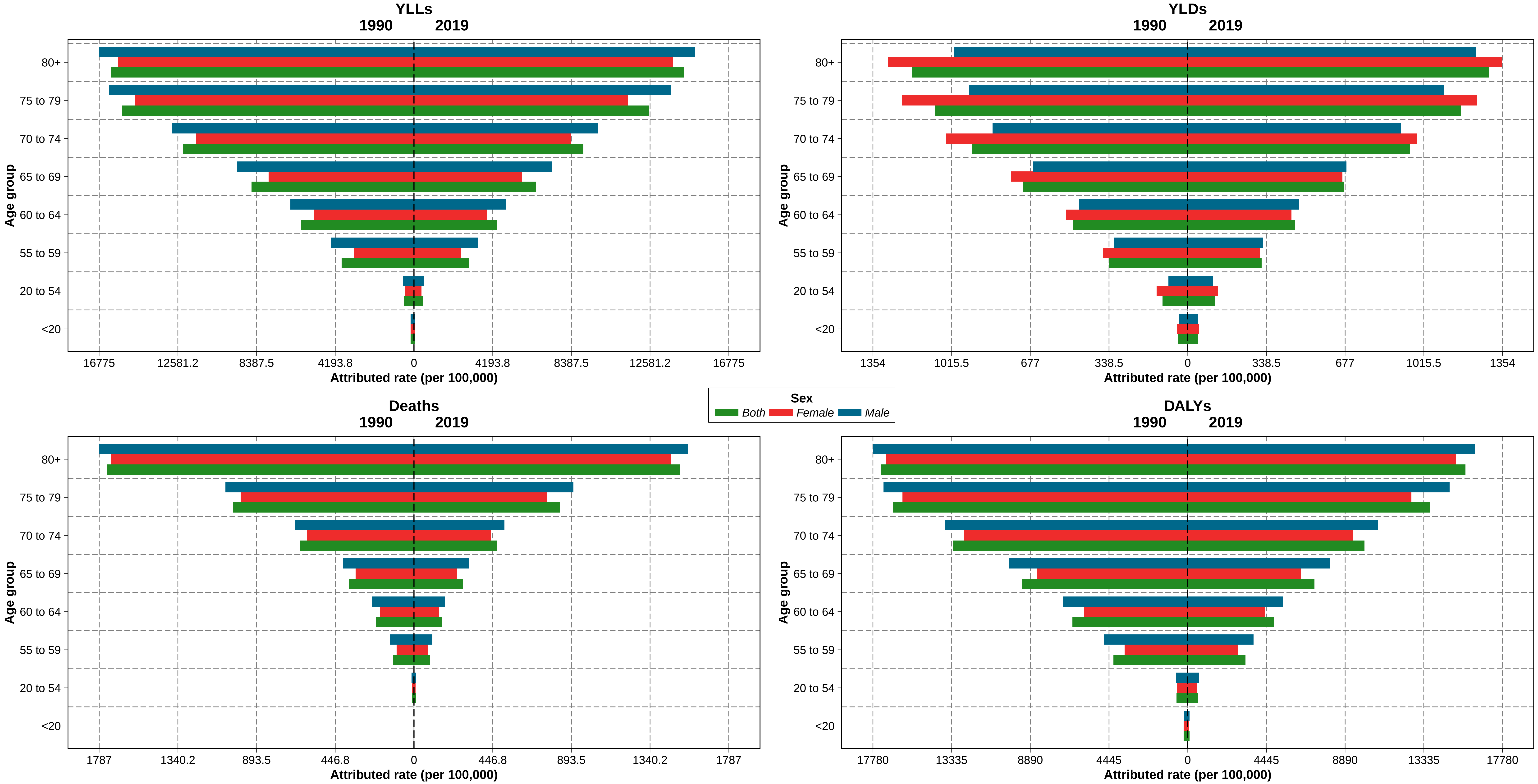

YLLs
1990
2019
80+
75 to 79
70 to 74
65 to 69
60 to 64
55 to 59
20 to 54
<20
Age group
16775
12581.2
8387.5
4193.8
0
4193.8
8387.5
12581.2
16775
Attributed rate (per 100,000)
YLDs
1990
2019
1354
1015.5
677
338.5
0
338.5
677
1015.5
1354
Attributed rate (per 100,000)
Sex
Both
Female
Male
Deaths
1990
2019
1787
1340.2
893.5
446.8
0
446.8
893.5
1340.2
1787
Attributed rate (per 100,000)
DALYs
1990
2019
17780
13335
8890
4445
0
4445
8890
13335
17780
Attributed rate (per 100,000)

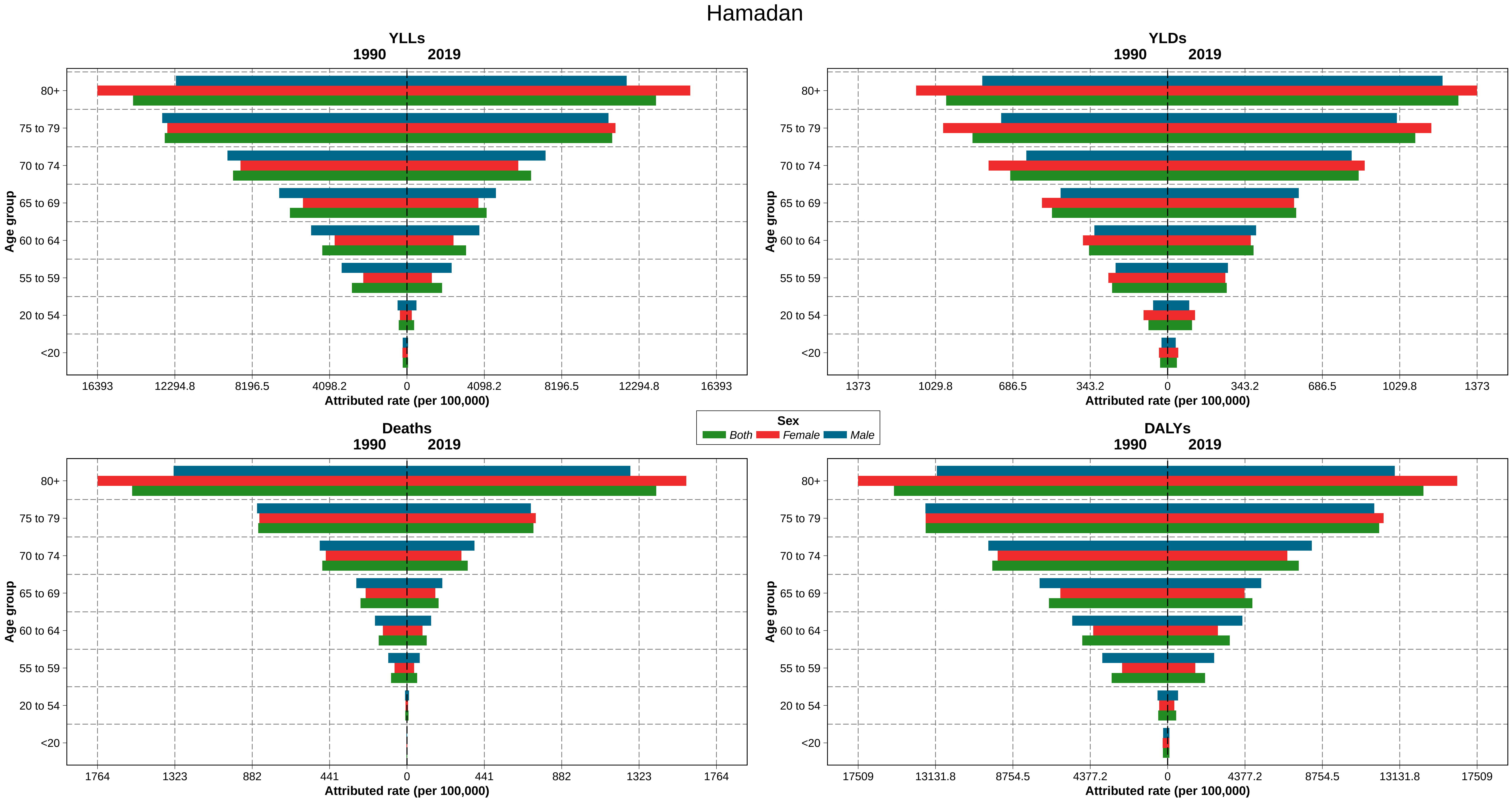
Hamadan
YLLs
1990
2019
80+
75 to 79
70 to 74
65 to 69
60 to 64
55 to 59
20 to 54
<20
Age group
16393
12294.8
8196.5
4098.2
0
4098.2
8196.5
12294.8
16393
Attributed rate (per 100,000)
YLDs
1990
2019
1373
1029.8
686.5
343.2
0
343.2
686.5
1029.8
1373
Attributed rate (per 100,000)
Sex
Both
Female
Male
Deaths
1990
2019
1764
1323
882
441
0
441
882
1323
1764
Attributed rate (per 100,000)
DALYs
1990
2019
17509
13131.8
8754.5
4377.2
0
4377.2
8754.5
13131.8
17509
Attributed rate (per 100,000)

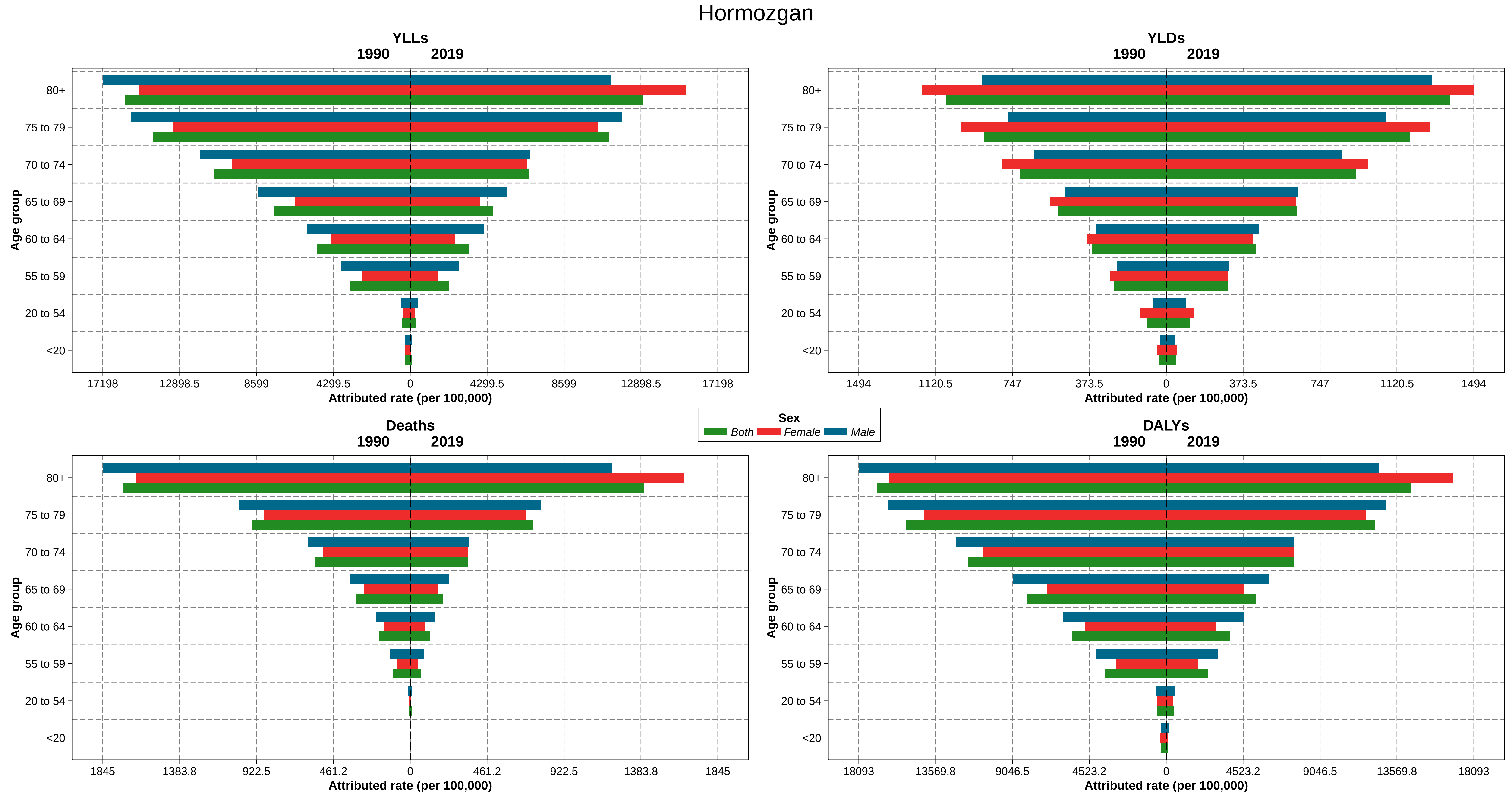

Hormozgan
YLLs
YLDs
Deaths
DALYs
1990
2019
Age group
80+
75 to 79
70 to 74
65 to 69
60 to 64
55 to 59
20 to 54
<20
17198
12898.5
8599
4299.5
0
1494
1120.5
747
373.5
1845
1383.8
922.5
461.2
18093
13569.8
9046.5
4523.2
Attributed rate (per 100,000)
Sex
Both
Female
Male

Ilam

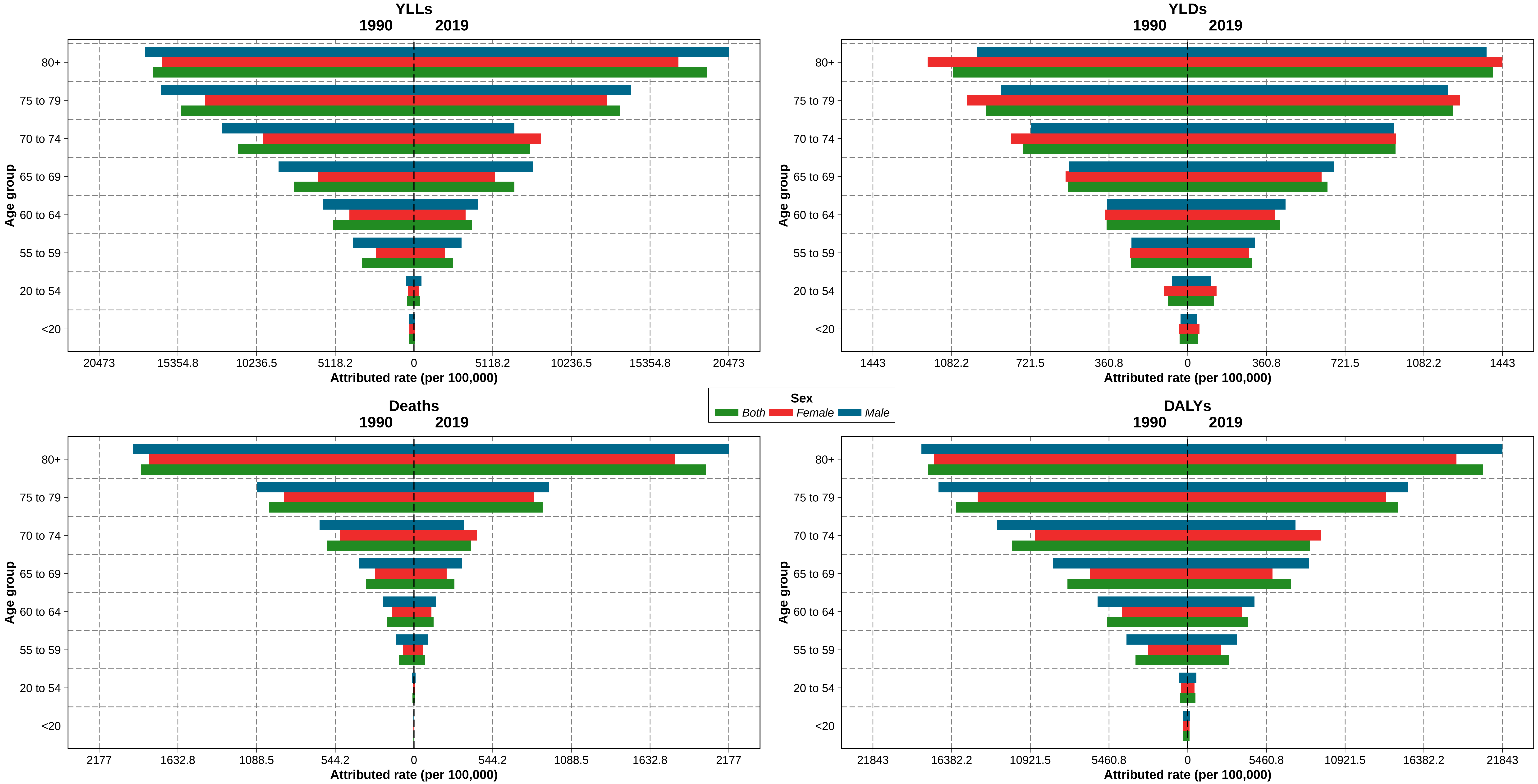

YLLs
1990
2019
80+
75 to 79
70 to 74
65 to 69
60 to 64
55 to 59
20 to 54
<20
Age group
20473
15354.8
10236.5
5118.2
0
5118.2
10236.5
15354.8
20473
Attributed rate (per 100,000)
YLDs
1990
2019
1443
1082.2
721.5
360.8
0
360.8
721.5
1082.2
1443
Attributed rate (per 100,000)
Sex
Both
Female
Male
Deaths
1990
2019
2177
1632.8
1088.5
544.2
0
544.2
1088.5
1632.8
2177
Attributed rate (per 100,000)
DALYs
1990
2019
21843
16382.2
10921.5
5460.8
0
5460.8
10921.5
16382.2
21843
Attributed rate (per 100,000)

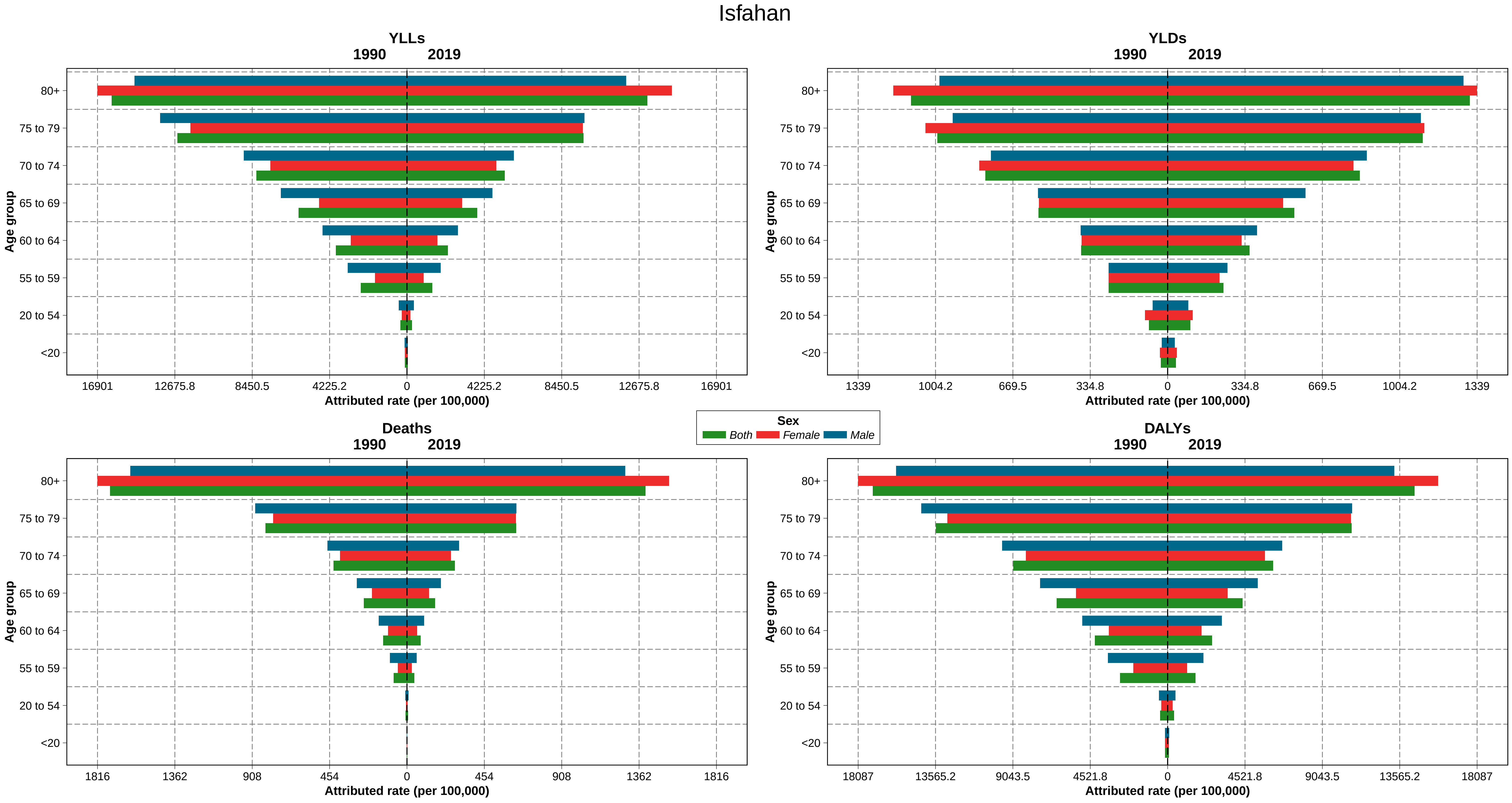

Isfahan
YLLs
1990
2019
YLDs
Deaths
DALYs
Age group
80+
75 to 79
70 to 74
65 to 69
60 to 64
55 to 59
20 to 54
<20
16901
12675.8
8450.5
4225.2
0
1339
1004.2
669.5
334.8
1816
1362
908
454
18087
13565.2
9043.5
4521.8
Attributed rate (per 100,000)
Sex
Both
Female
Male

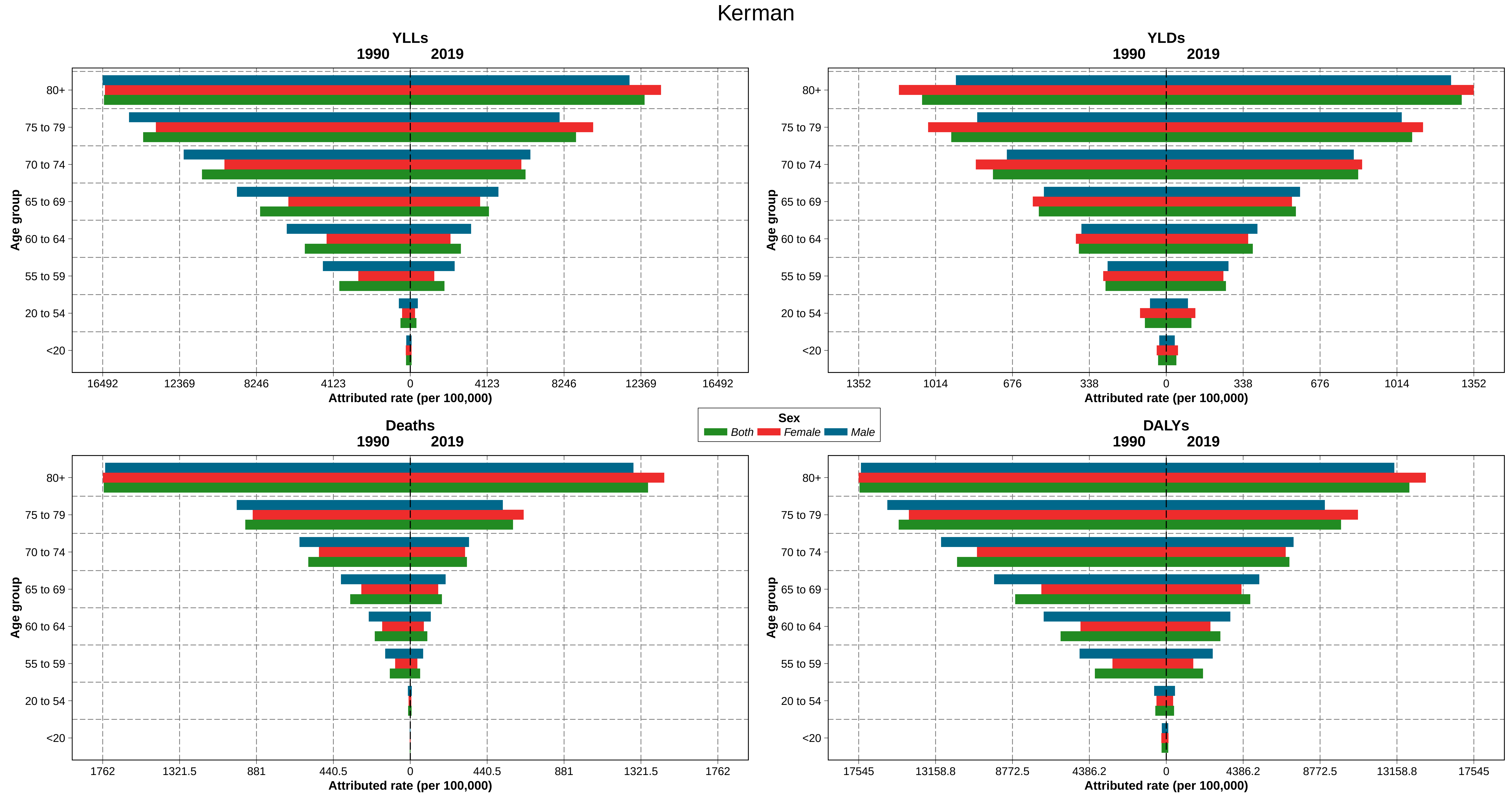
Kerman
YLLs
1990
2019
80+
75 to 79
70 to 74
65 to 69
60 to 64
55 to 59
20 to 54
<20
Age group
16492
12369
8246
4123
0
4123
8246
12369
16492
Attributed rate (per 100,000)
YLDs
1990
2019
1352
1014
676
338
0
338
676
1014
1352
Attributed rate (per 100,000)
Sex
Both
Female
Male
Deaths
1990
2019
1762
1321.5
881
440.5
0
440.5
881
1321.5
1762
Attributed rate (per 100,000)
DALYs
1990
2019
17545
13158.8
8772.5
4386.2
0
4386.2
8772.5
13158.8
17545
Attributed rate (per 100,000)

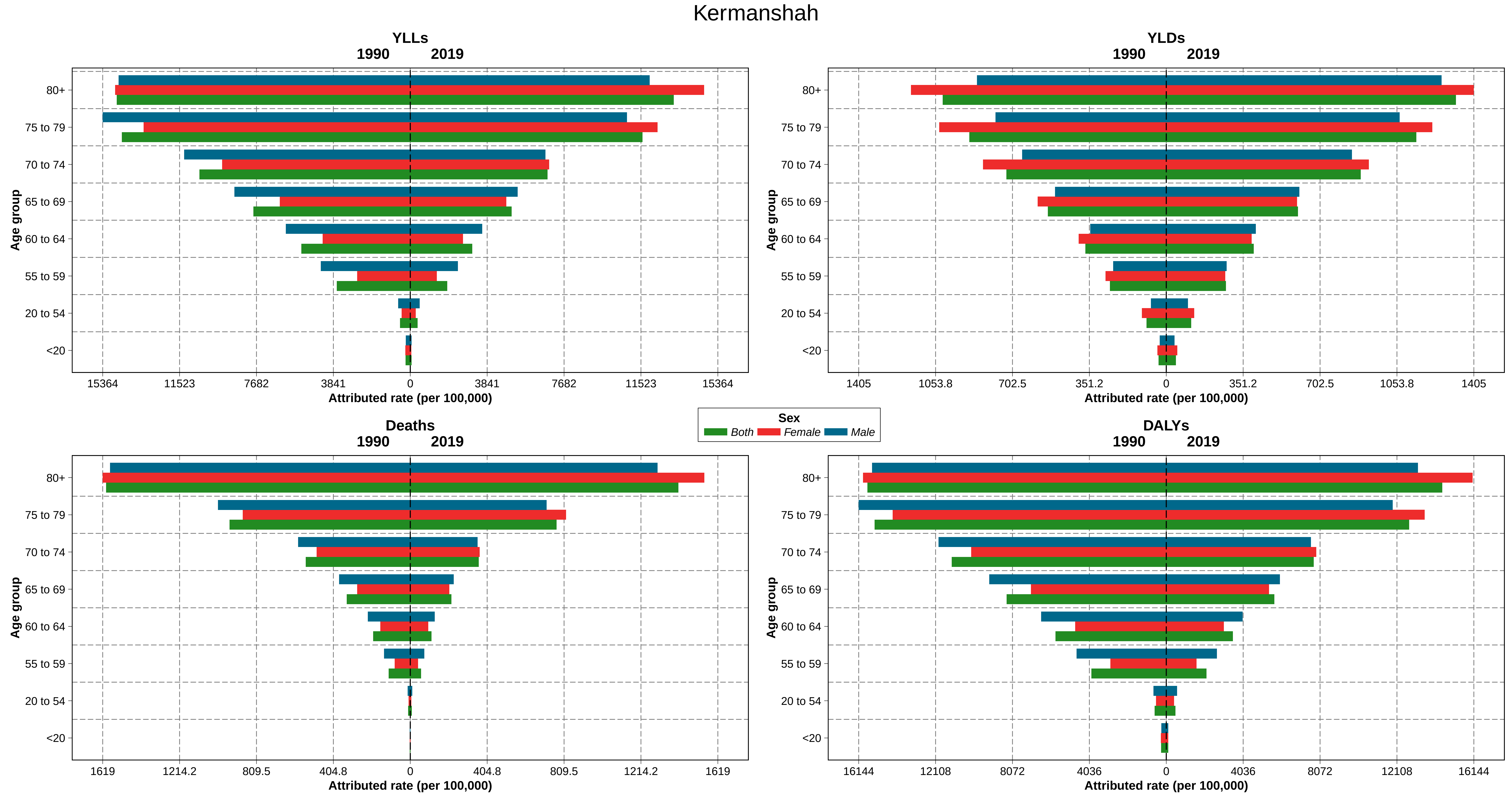
Kermanshah
YLLs
1990
2019
YLDs
Deaths
DALYs
80+
75 to 79
70 to 74
65 to 69
60 to 64
55 to 59
20 to 54
<20
Age group
Attributed rate (per 100,000)
15364
11523
7682
3841
0
1405
1053.8
702.5
351.2
1619
1214.2
809.5
404.8
16144
12108
8072
4036
Sex
Both
Female
Male

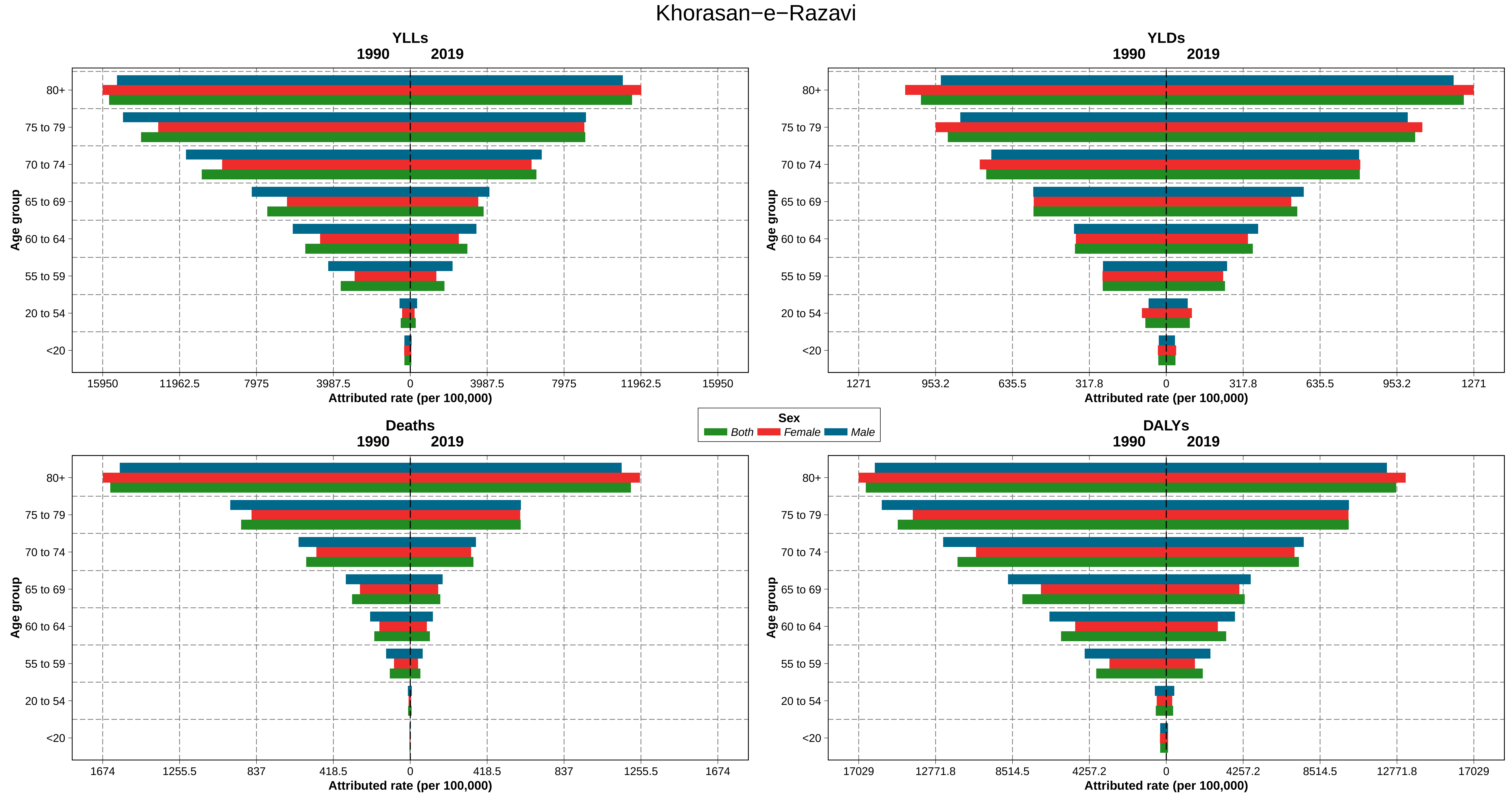

Khorasan−e−Razavi
YLLs
1990
2019
YLDs
Deaths
DALYs
Age group
80+
75 to 79
70 to 74
65 to 69
60 to 64
55 to 59
20 to 54
<20
15950 11962.5 7975 3987.5 0 3987.5 7975 11962.5 15950
1271 953.2 635.5 317.8 0 317.8 635.5 953.2 1271
1674 1255.5 837 418.5 0 418.5 837 1255.5 1674
17029 12771.8 8514.5 4257.2 0 4257.2 8514.5 12771.8 17029
Attributed rate (per 100,000)
Sex
Both
Female
Male

Khuzestan

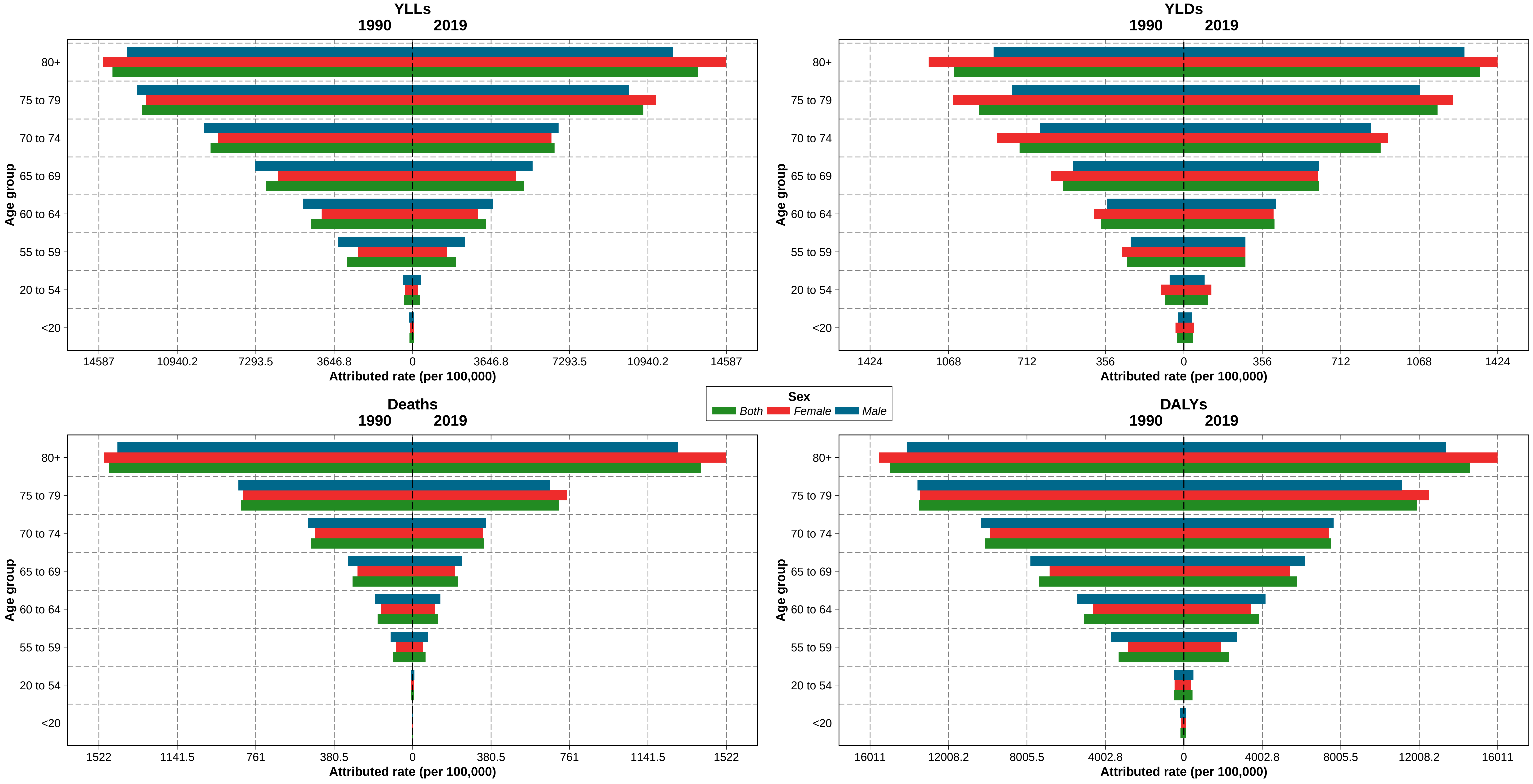
YLLs
1990
2019
80+
75 to 79
70 to 74
65 to 69
60 to 64
55 to 59
20 to 54
<20
Age group
14587
10940.2
7293.5
3646.8
0
3646.8
7293.5
10940.2
14587
Attributed rate (per 100,000)
YLDs
1990
2019
1424
1068
712
356
0
356
712
1068
1424
Attributed rate (per 100,000)
Sex
Both
Female
Male
Deaths
1990
2019
1522
1141.5
761
380.5
0
380.5
761
1141.5
1522
Attributed rate (per 100,000)
DALYs
1990
2019
16011
12008.2
8005.5
4002.8
0
4002.8
8005.5
12008.2
16011
Attributed rate (per 100,000)

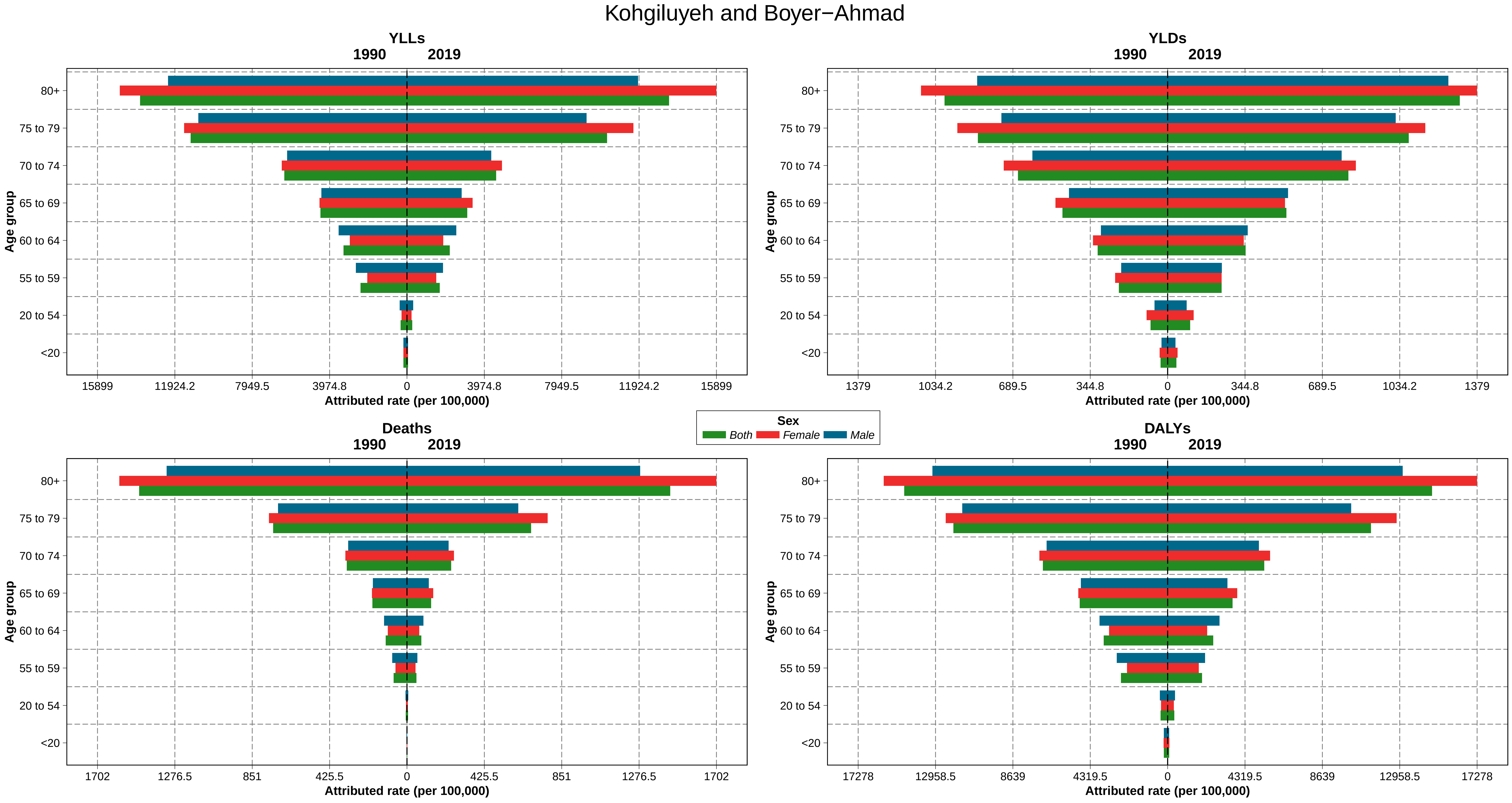
Kohgiluyeh and Boyer−Ahmad
YLLs
1990
2019
80+
75 to 79
70 to 74
65 to 69
60 to 64
55 to 59
20 to 54
<20
Age group
15899
11924.2
7949.5
3974.8
0
3974.8
7949.5
11924.2
15899
Attributed rate (per 100,000)
YLDs
1990
2019
1379
1034.2
689.5
344.8
0
344.8
689.5
1034.2
1379
Attributed rate (per 100,000)
Sex
Both
Female
Male
Deaths
1990
2019
1702
1276.5
851
425.5
0
425.5
851
1276.5
1702
Attributed rate (per 100,000)
DALYs
1990
2019
17278
12958.5
8639
4319.5
0
4319.5
8639
12958.5
17278
Attributed rate (per 100,000)

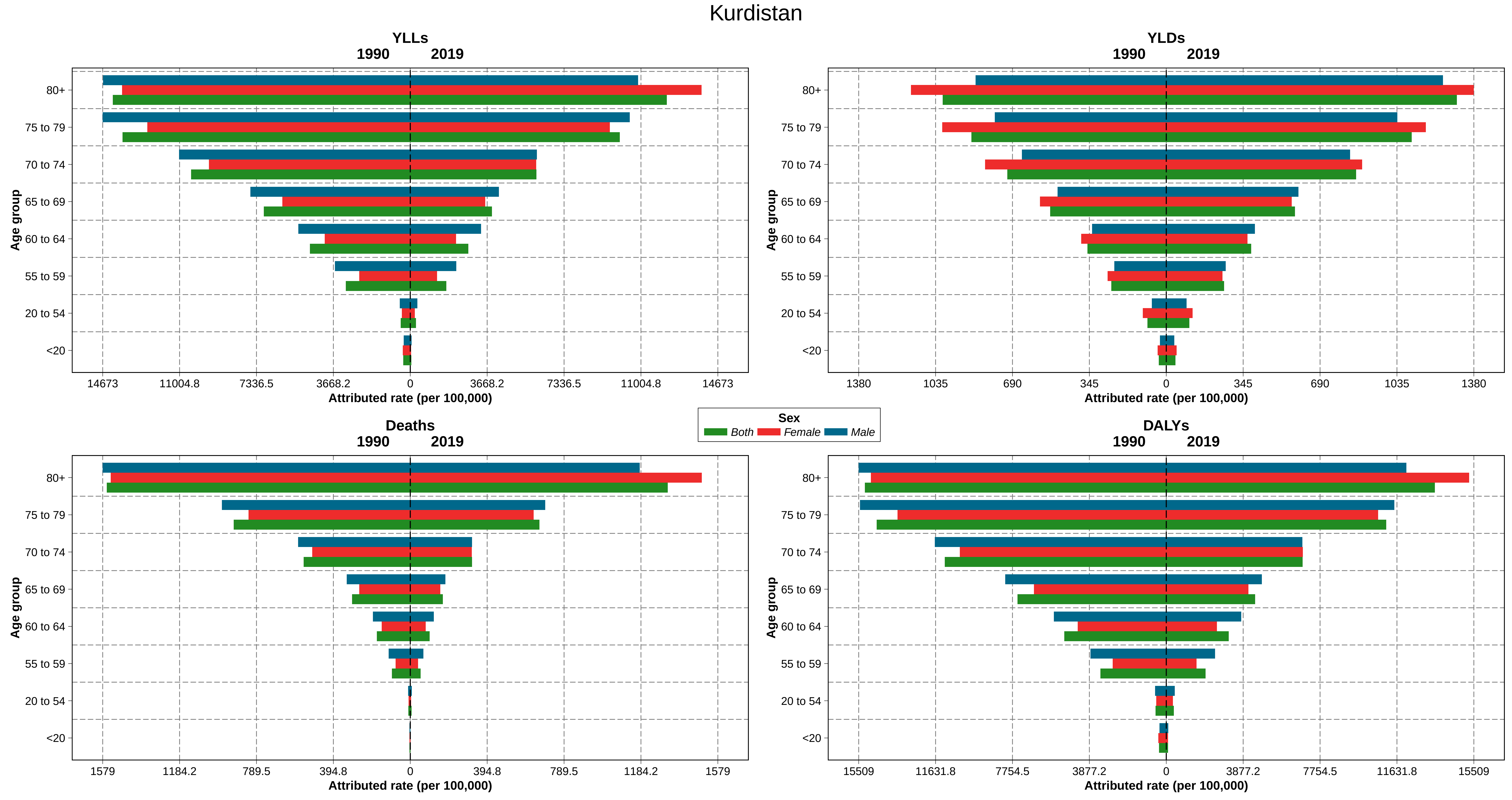

Kurdistan
YLLs
1990
2019
YLDs
Deaths
DALYs
Age group
80+
75 to 79
70 to 74
65 to 69
60 to 64
55 to 59
20 to 54
<20
14673
11004.8
7336.5
3668.2
0
1380
1035
690
345
1579
1184.2
789.5
394.8
15509
11631.8
7754.5
3877.2
Attributed rate (per 100,000)
Sex
Both
Female
Male

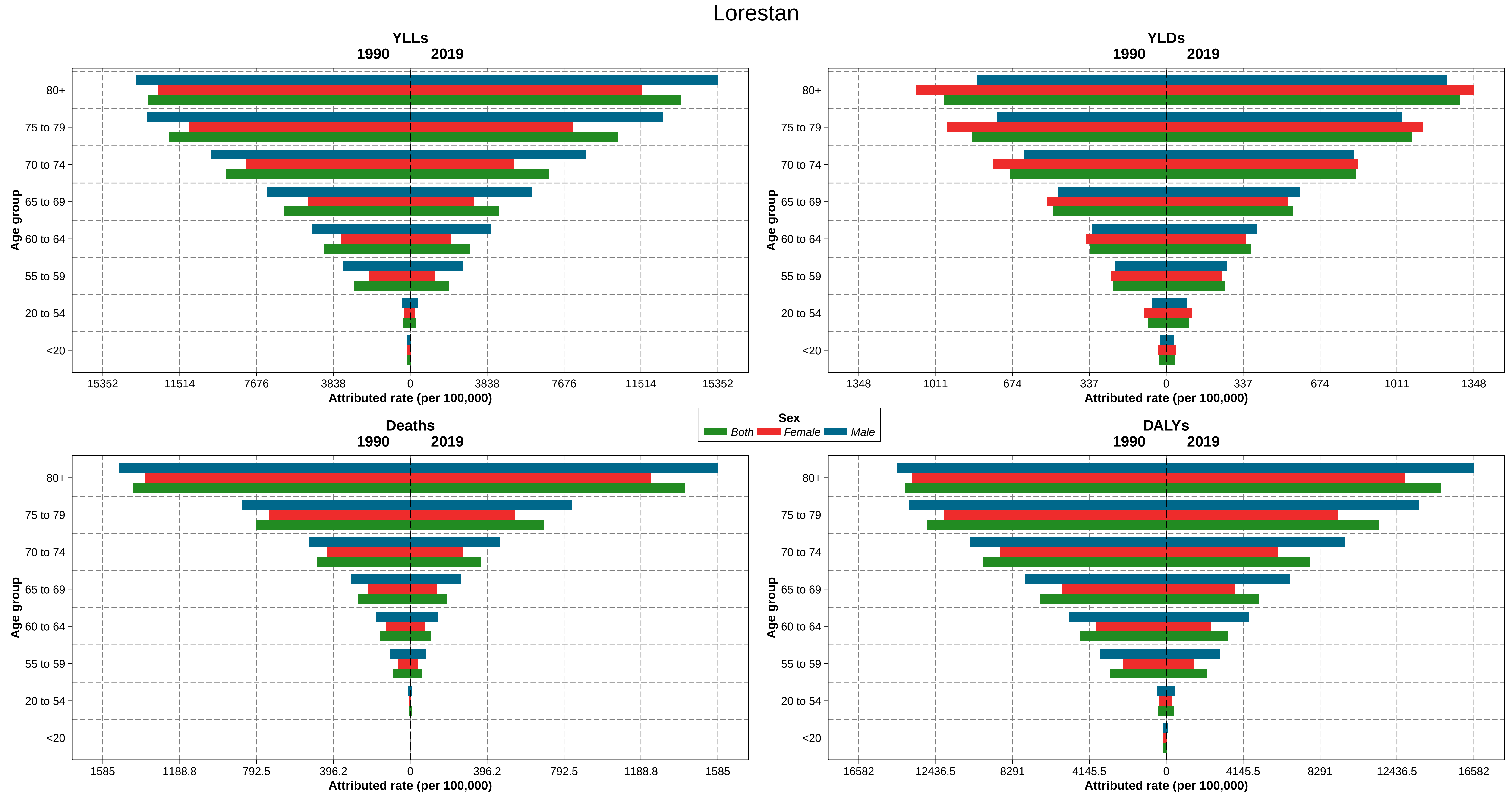

Lorestan
YLLs
1990
2019
80+
75 to 79
70 to 74
65 to 69
60 to 64
55 to 59
20 to 54
<20
Age group
15352
11514
7676
3838
0
3838
7676
11514
15352
Attributed rate (per 100,000)
YLDs
1990
2019
1348
1011
674
337
0
337
674
1011
1348
Attributed rate (per 100,000)
Sex
Both
Female
Male
Deaths
1990
2019
1585
1188.8
792.5
396.2
0
396.2
792.5
1188.8
1585
Attributed rate (per 100,000)
DALYs
1990
2019
16582
12436.5
8291
4145.5
0
4145.5
8291
12436.5
16582
Attributed rate (per 100,000)

Markazi

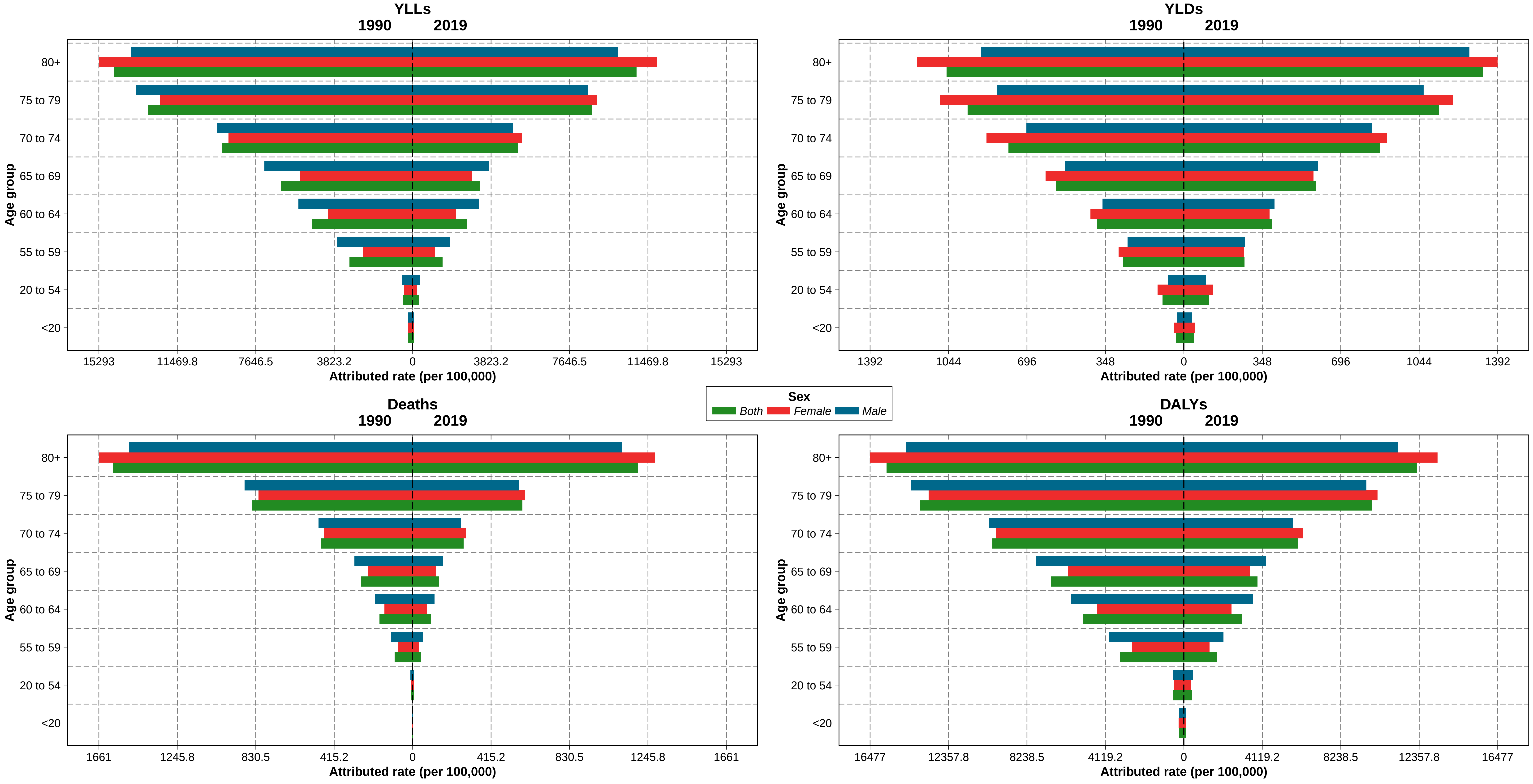
YLLs
1990
2019
YLDs
1990
2019
Deaths
1990
2019
DALYs
1990
2019
Age group
80+
75 to 79
70 to 74
65 to 69
60 to 64
55 to 59
20 to 54
<20
15293
11469.8
7646.5
3823.2
0
1392
1044
696
348
1661
1245.8
830.5
415.2
16477
12357.8
8238.5
4119.2
Attributed rate (per 100,000)
Sex
Both
Female
Male

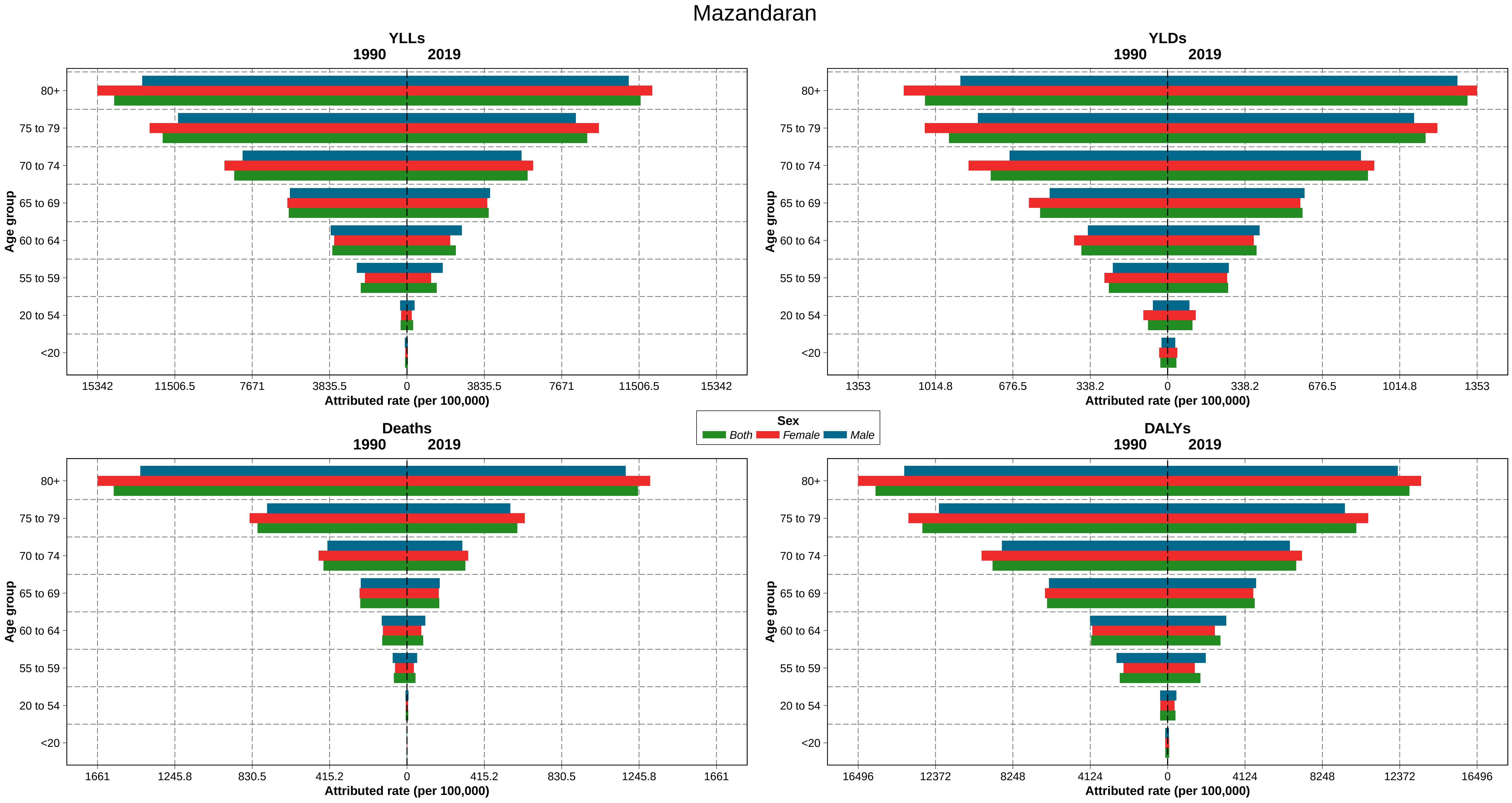

Mazandaran
YLLs
1990
2019
80+
75 to 79
70 to 74
65 to 69
60 to 64
55 to 59
20 to 54
<20
Age group
15342
11506.5
7671
3835.5
0
3835.5
7671
11506.5
15342
Attributed rate (per 100,000)
YLDs
1990
2019
1353
1014.8
676.5
338.2
0
338.2
676.5
1014.8
1353
Attributed rate (per 100,000)
Sex
Both
Female
Male
Deaths
1990
2019
1661
1245.8
830.5
415.2
0
415.2
830.5
1245.8
1661
Attributed rate (per 100,000)
DALYs
1990
2019
16496
12372
8248
4124
0
4124
8248
12372
16496
Attributed rate (per 100,000)

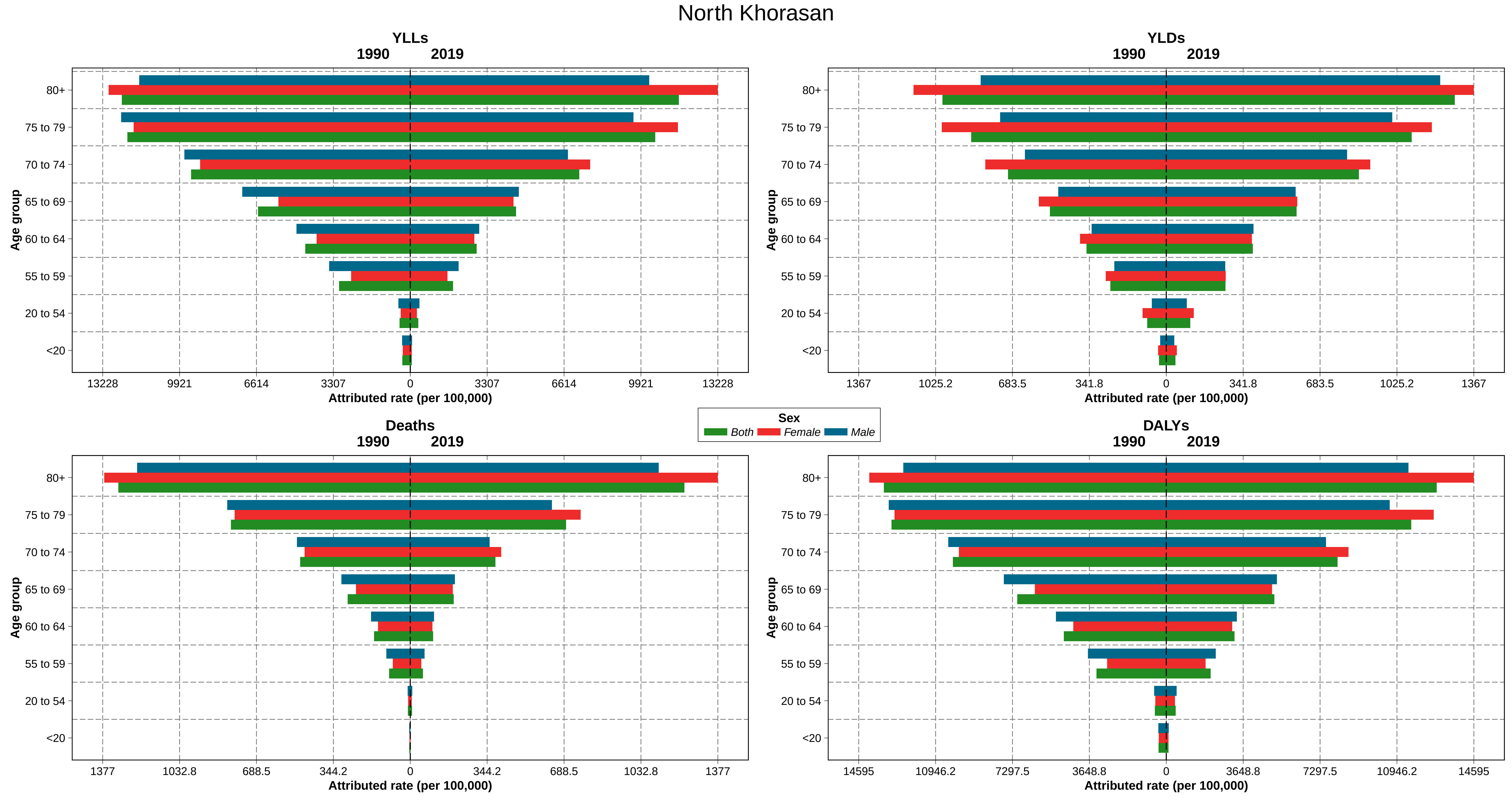
North Khorasan
YLLs
1990
2019
YLDs
1990
2019
Deaths
1990
2019
DALYs
1990
2019
Age group
80+
75 to 79
70 to 74
65 to 69
60 to 64
55 to 59
20 to 54
<20
13228
9921
6614
3307
0
3307
6614
9921
13228
1367
1025.2
683.5
341.8
0
341.8
683.5
1025.2
1367
1377
1032.8
688.5
344.2
0
344.2
688.5
1032.8
1377
14595
10946.2
7297.5
3648.8
0
3648.8
7297.5
10946.2
14595
Attributed rate (per 100,000)
Sex
Both
Female
Male

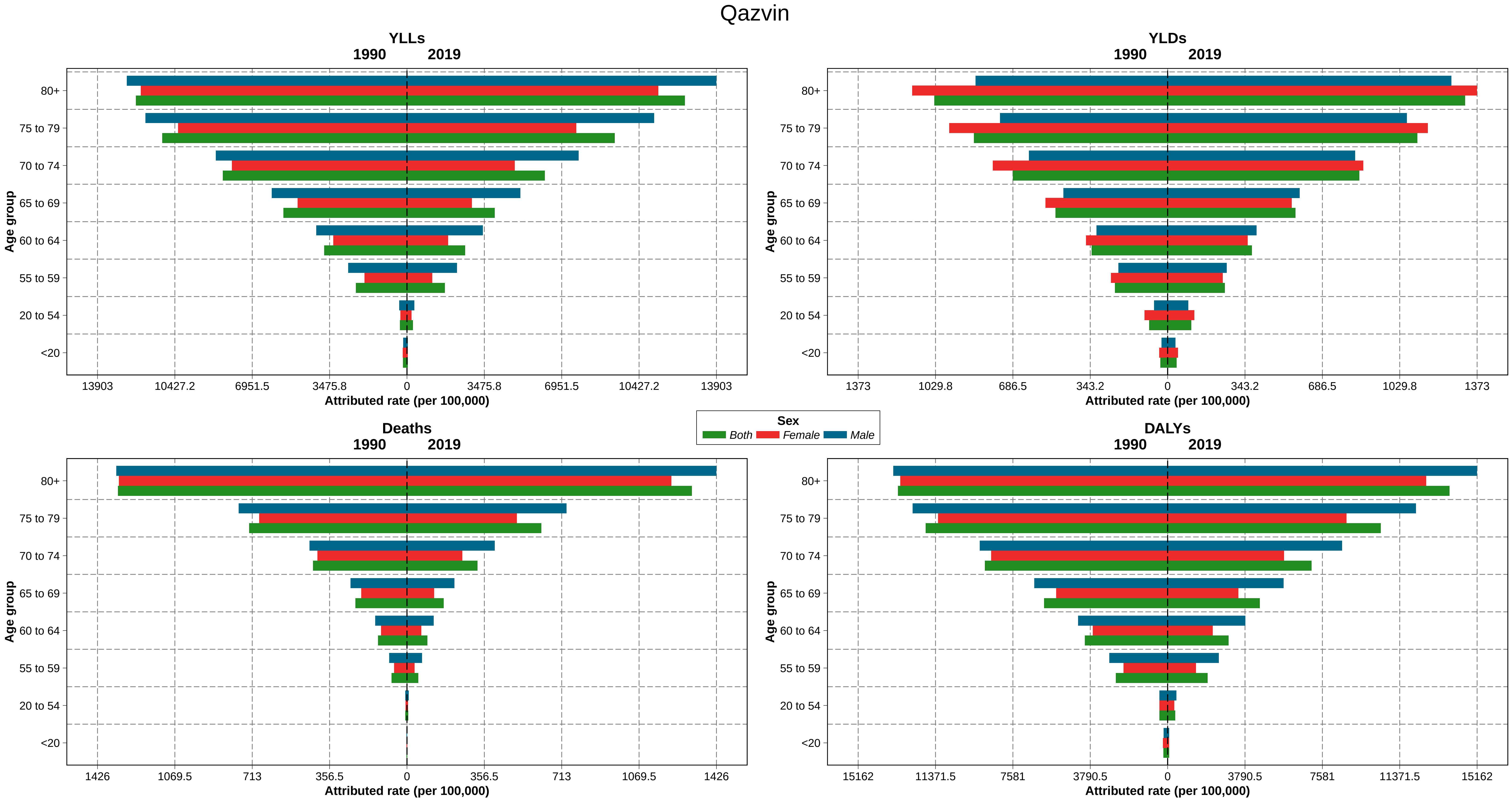

Qazvin
YLLs
1990
2019
80+
75 to 79
70 to 74
65 to 69
60 to 64
55 to 59
20 to 54
<20
Age group
13903
10427.2
6951.5
3475.8
0
3475.8
6951.5
10427.2
13903
Attributed rate (per 100,000)
YLDs
1990
2019
1373
1029.8
686.5
343.2
0
343.2
686.5
1029.8
1373
Attributed rate (per 100,000)
Sex
Both
Female
Male
Deaths
1990
2019
1426
1069.5
713
356.5
0
356.5
713
1069.5
1426
Attributed rate (per 100,000)
DALYs
1990
2019
15162
11371.5
7581
3790.5
0
3790.5
7581
11371.5
15162
Attributed rate (per 100,000)

Qom

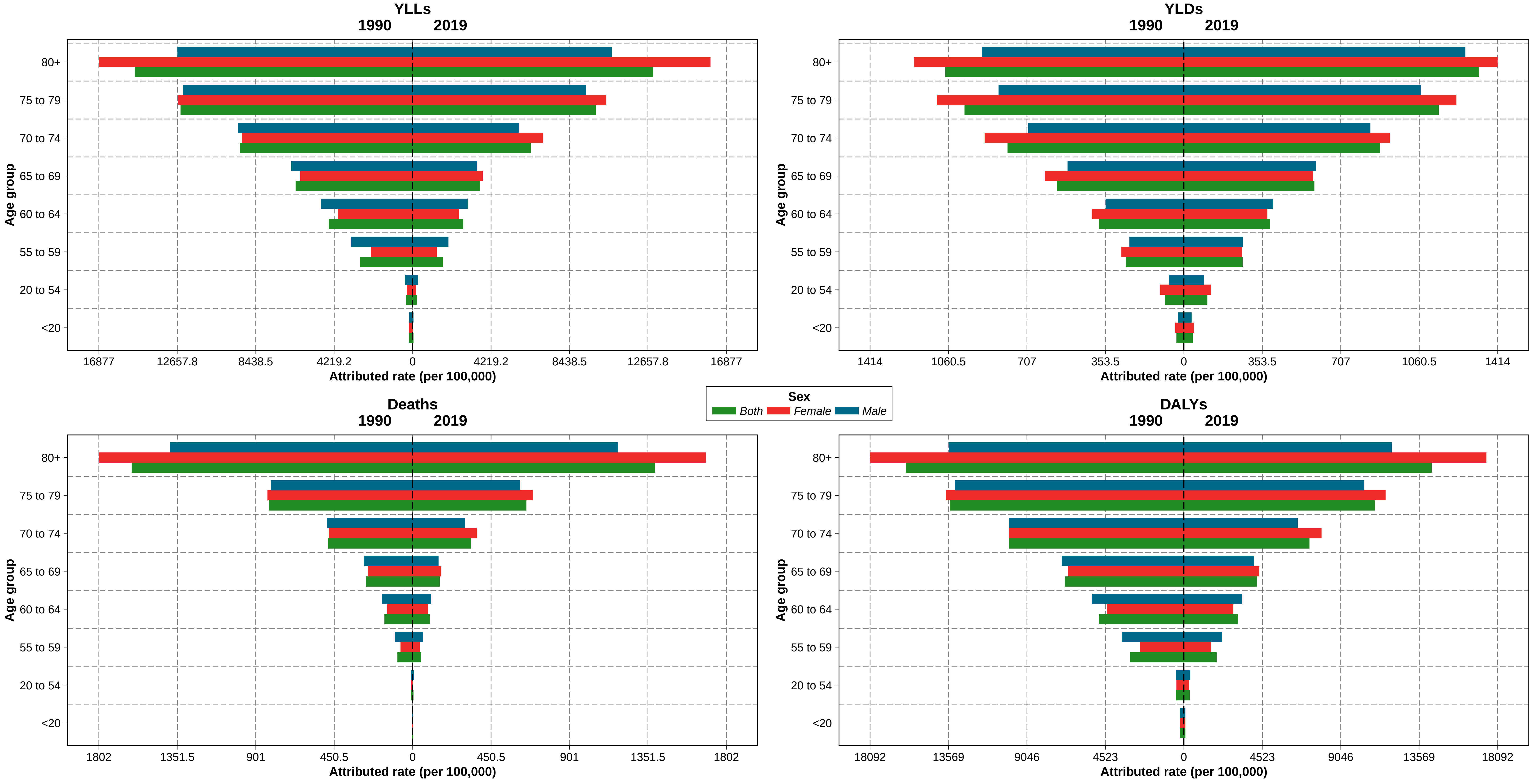

YLLs
1990
2019
80+
75 to 79
70 to 74
65 to 69
60 to 64
55 to 59
20 to 54
<20
Age group
16877
12657.8
8438.5
4219.2
0
4219.2
8438.5
12657.8
16877
Attributed rate (per 100,000)
YLDs
1990
2019
1414
1060.5
707
353.5
0
353.5
707
1060.5
1414
Attributed rate (per 100,000)
Sex
Both
Female
Male
Deaths
1990
2019
1802
1351.5
901
450.5
0
450.5
901
1351.5
1802
Attributed rate (per 100,000)
DALYs
1990
2019
18092
13569
9046
4523
0
4523
9046
13569
18092
Attributed rate (per 100,000)

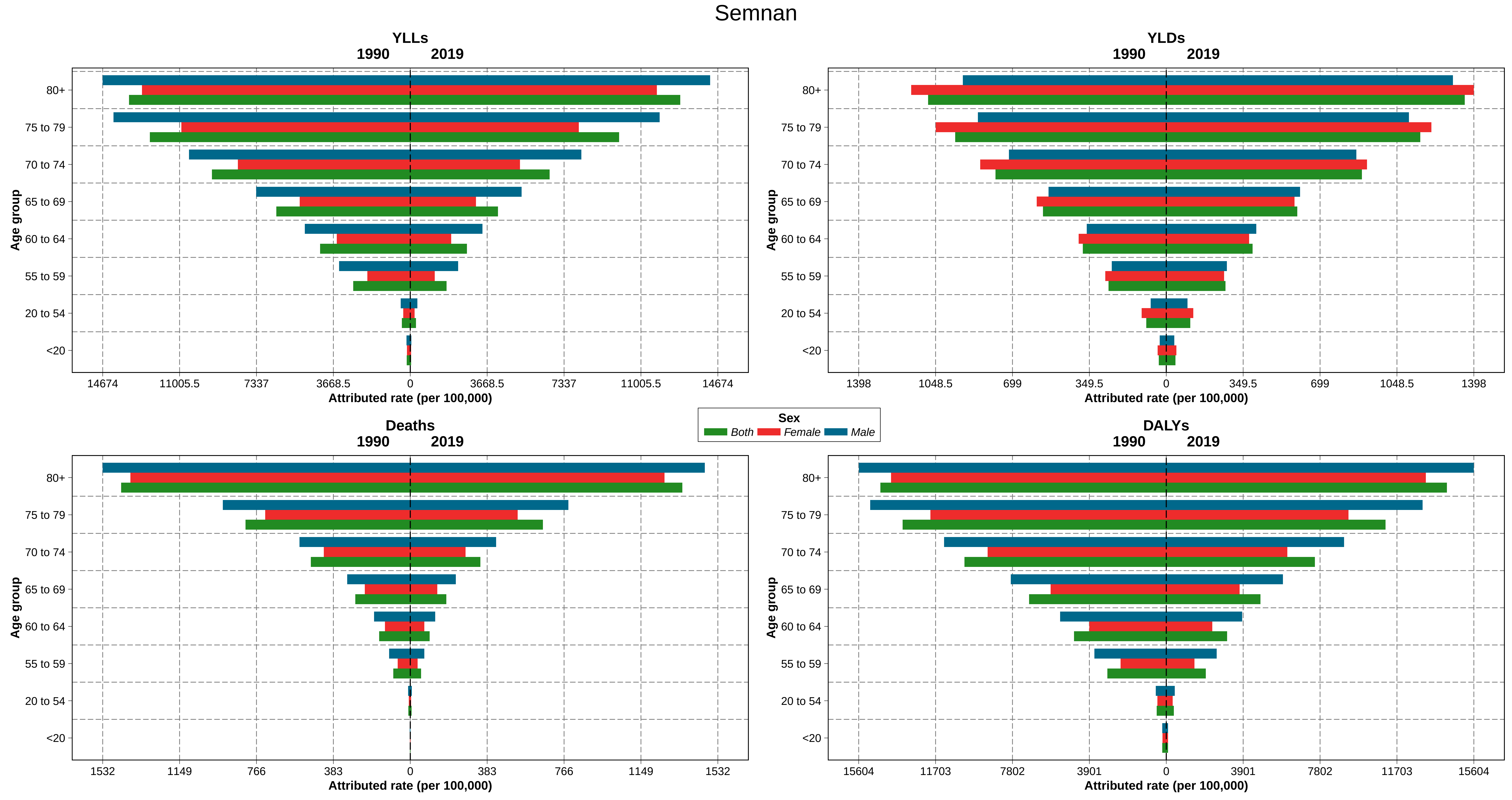

Semnan
YLLs
1990
2019
80+
75 to 79
70 to 74
65 to 69
60 to 64
55 to 59
20 to 54
<20
Age group
14674
11005.5
7337
3668.5
0
3668.5
7337
11005.5
14674
Attributed rate (per 100,000)
YLDs
1990
2019
1398
1048.5
699
349.5
0
349.5
699
1048.5
1398
Attributed rate (per 100,000)
Sex
Both
Female
Male
Deaths
1990
2019
1532
1149
766
383
0
383
766
1149
1532
Attributed rate (per 100,000)
DALYs
1990
2019
15604
11703
7802
3901
0
3901
7802
11703
15604
Attributed rate (per 100,000)

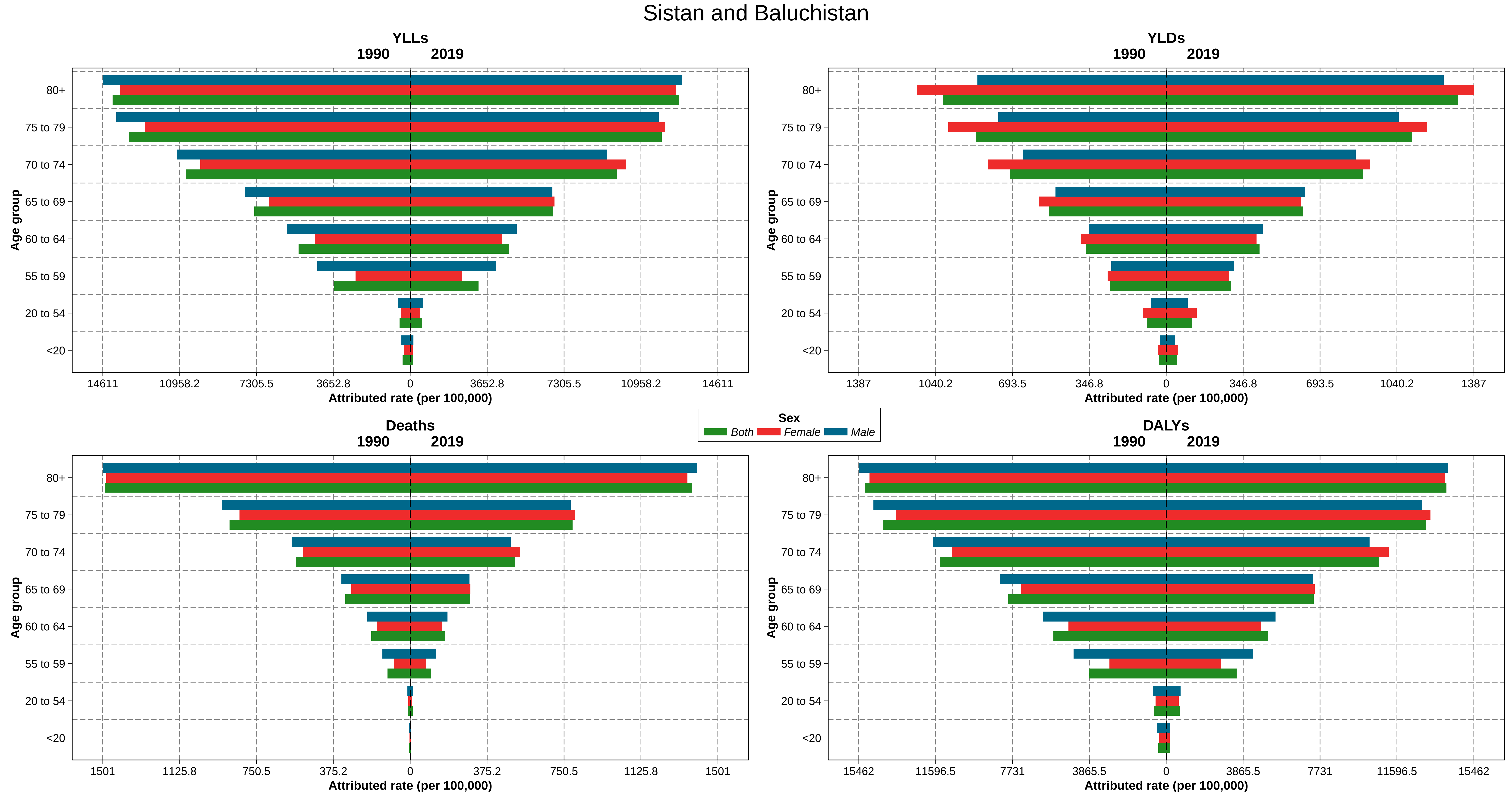
Sistan and Baluchistan
YLLs
1990
2019
80+
75 to 79
70 to 74
65 to 69
60 to 64
55 to 59
20 to 54
<20
Age group
14611
10958.2
7305.5
3652.8
0
3652.8
7305.5
10958.2
14611
Attributed rate (per 100,000)
YLDs
1990
2019
1387
1040.2
693.5
346.8
0
346.8
693.5
1040.2
1387
Attributed rate (per 100,000)
Sex
Both
Female
Male
Deaths
1990
2019
1501
1125.8
750.5
375.2
0
375.2
750.5
1125.8
1501
Attributed rate (per 100,000)
DALYs
1990
2019
15462
11596.5
7731
3865.5
0
3865.5
7731
11596.5
15462
Attributed rate (per 100,000)

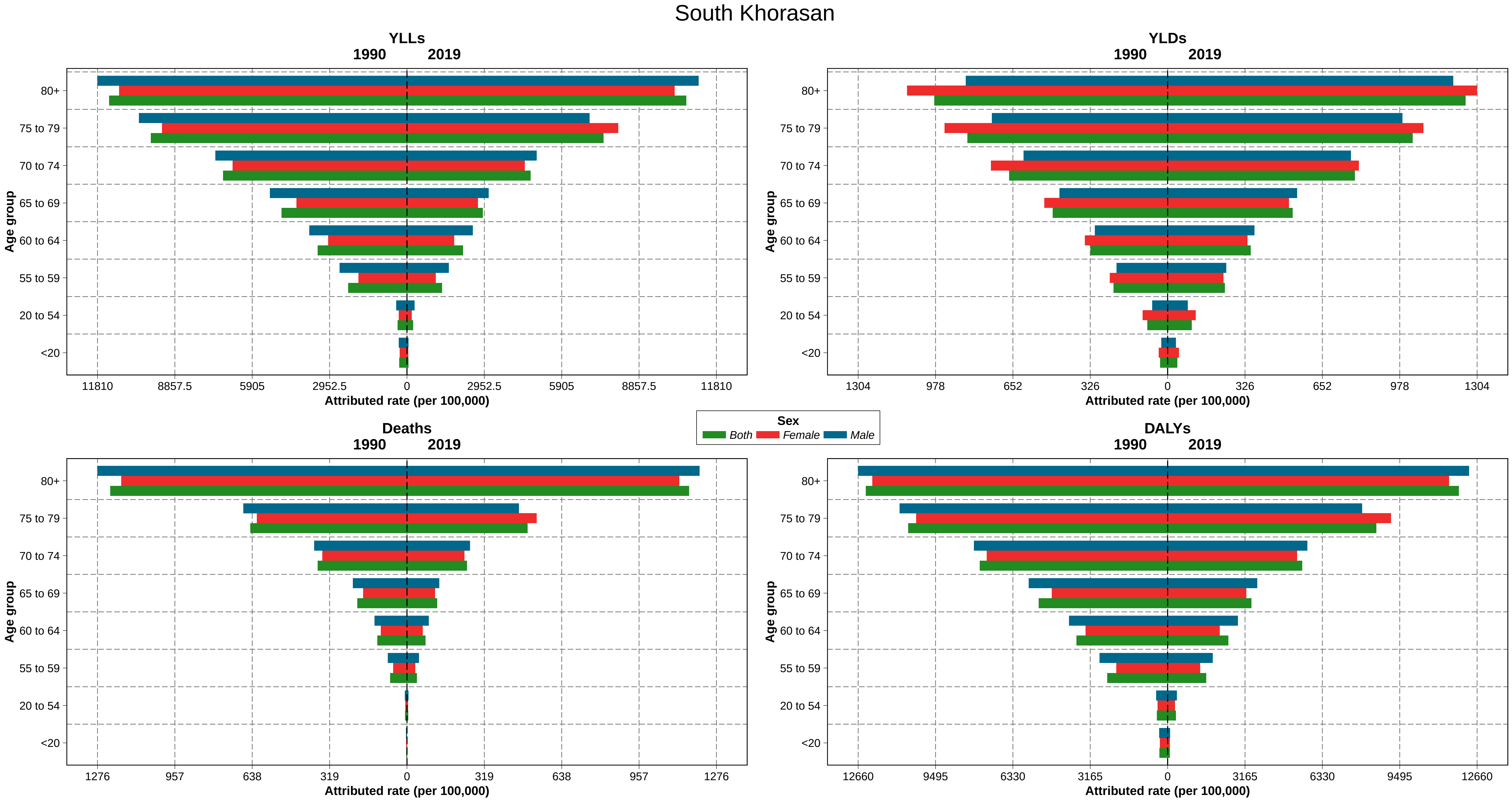
South Khorasan
YLLs
1990
2019
80+
75 to 79
70 to 74
65 to 69
60 to 64
55 to 59
20 to 54
<20
Age group
11810
8857.5
5905
2952.5
0
2952.5
5905
8857.5
11810
Attributed rate (per 100,000)
YLDs
1990
2019
1304
978
652
326
0
326
652
978
1304
Attributed rate (per 100,000)
Sex
Both
Female
Male
Deaths
1990
2019
1276
957
638
319
0
319
638
957
1276
Attributed rate (per 100,000)
DALYs
1990
2019
12660
9495
6330
3165
0
3165
6330
9495
12660
Attributed rate (per 100,000)

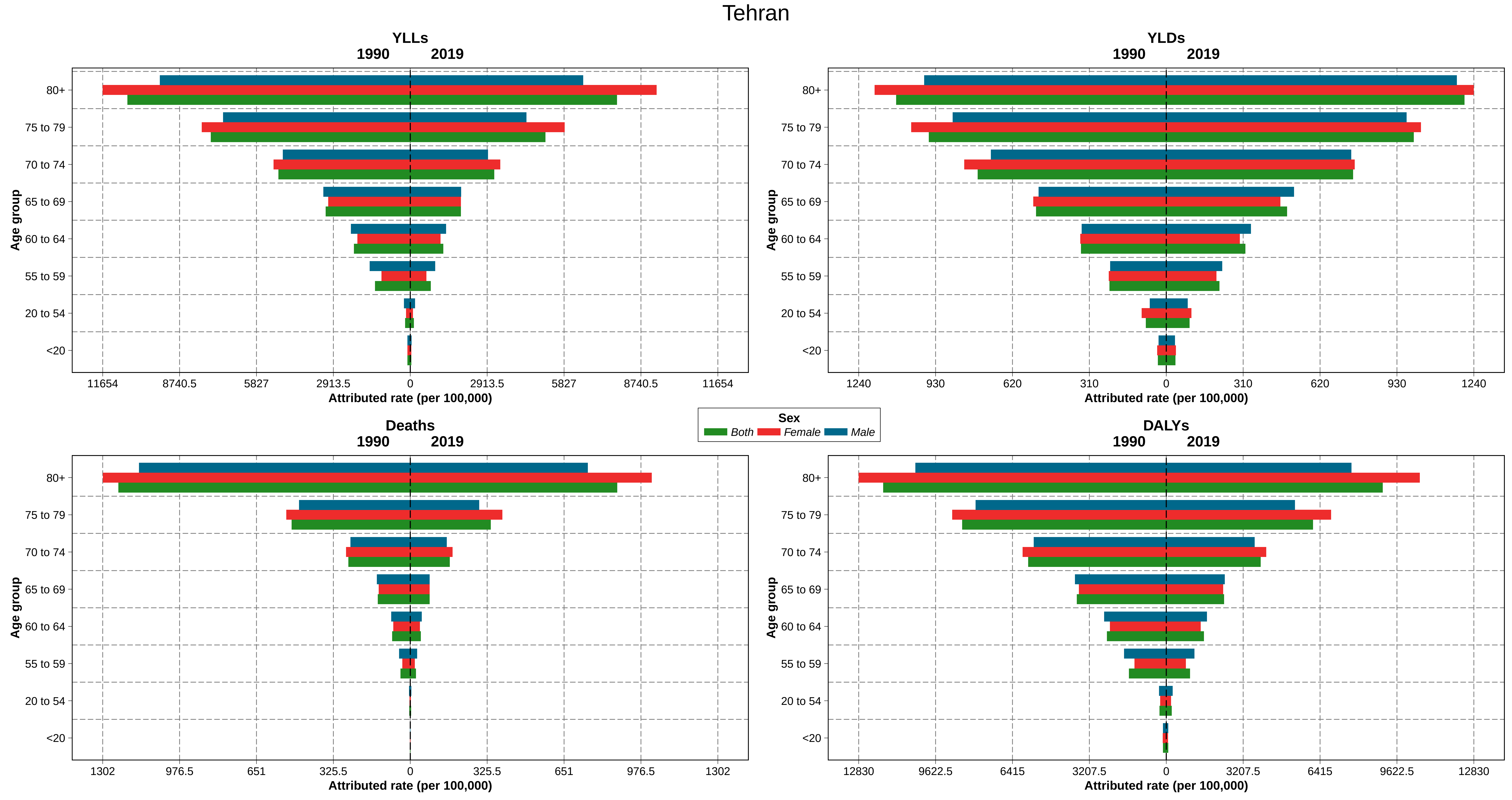

Tehran
YLLs
1990
2019
80+
75 to 79
70 to 74
65 to 69
60 to 64
55 to 59
20 to 54
<20
Age group
11654
8740.5
5827
2913.5
0
2913.5
5827
8740.5
11654
Attributed rate (per 100,000)
YLDs
1990
2019
1240
930
620
310
0
310
620
930
1240
Attributed rate (per 100,000)
Sex
Both
Female
Male
Deaths
1990
2019
1302
976.5
651
325.5
0
325.5
651
976.5
1302
Attributed rate (per 100,000)
DALYs
1990
2019
12830
9622.5
6415
3207.5
0
3207.5
6415
9622.5
12830
Attributed rate (per 100,000)

West Azarbayejan

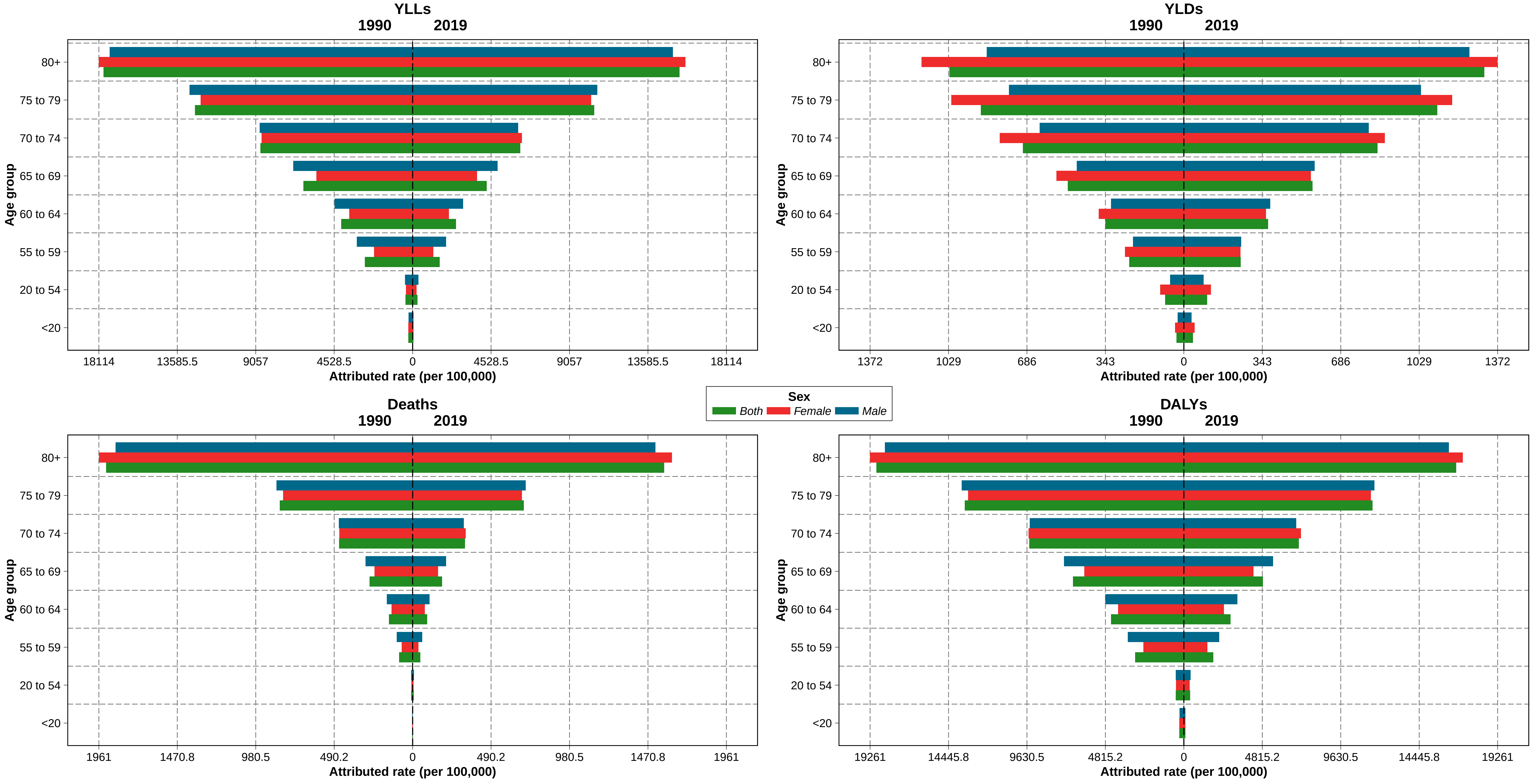

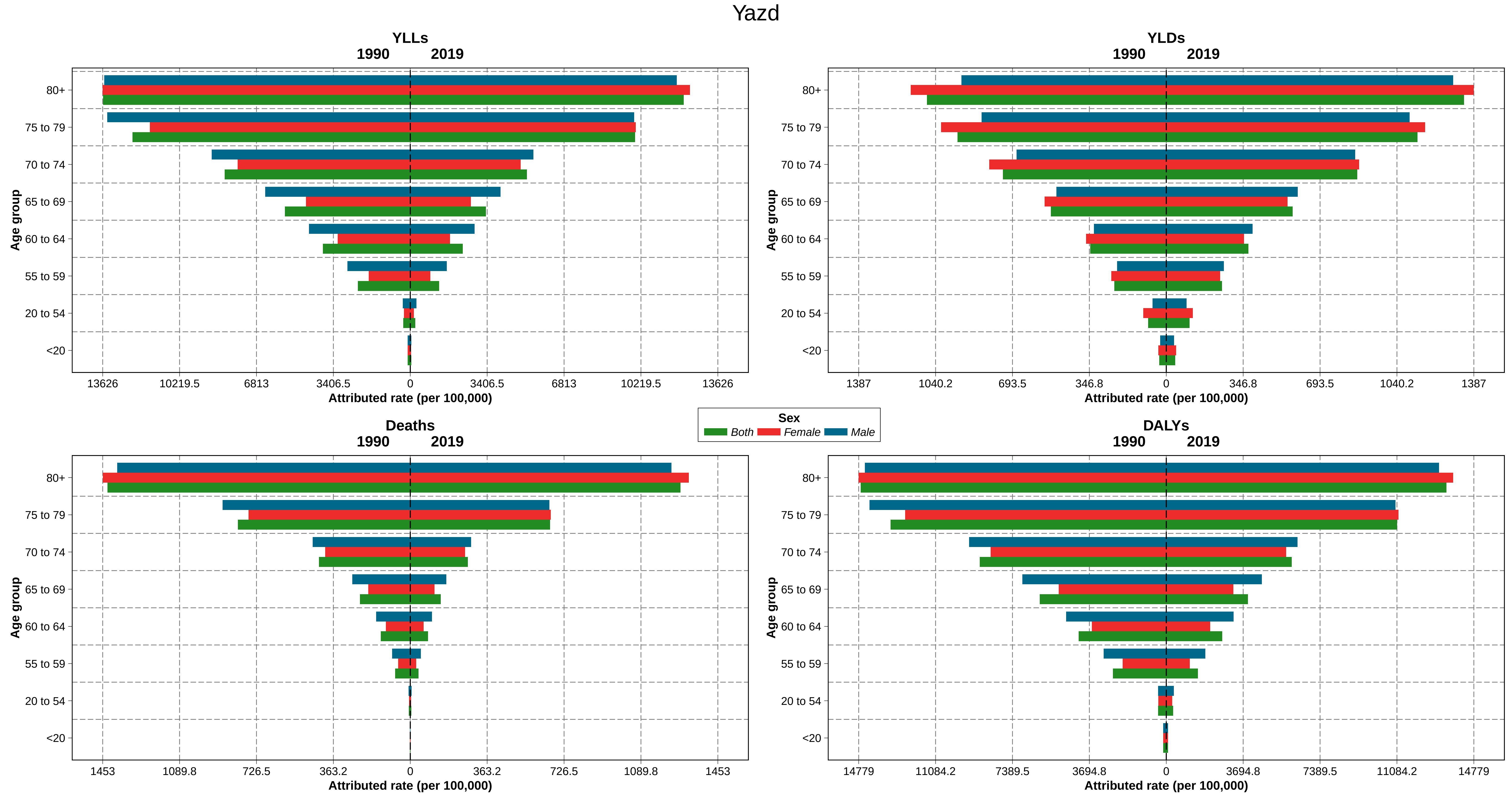

Yazd
YLLs
1990
2019
YLDs
1990
2019
Deaths
1990
2019
DALYs
1990
2019
Age group
80+
75 to 79
70 to 74
65 to 69
60 to 64
55 to 59
20 to 54
<20
13626
10219.5
6813
3406.5
0
3406.5
6813
10219.5
13626
1387
1040.2
693.5
346.8
0
346.8
693.5
1040.2
1387
1453
1089.8
726.5
363.2
0
363.2
726.5
1089.8
1453
14779
11084.2
7389.5
3694.8
0
3694.8
7389.5
11084.2
14779
Attributed rate (per 100,000)
Sex
Both
Female
Male

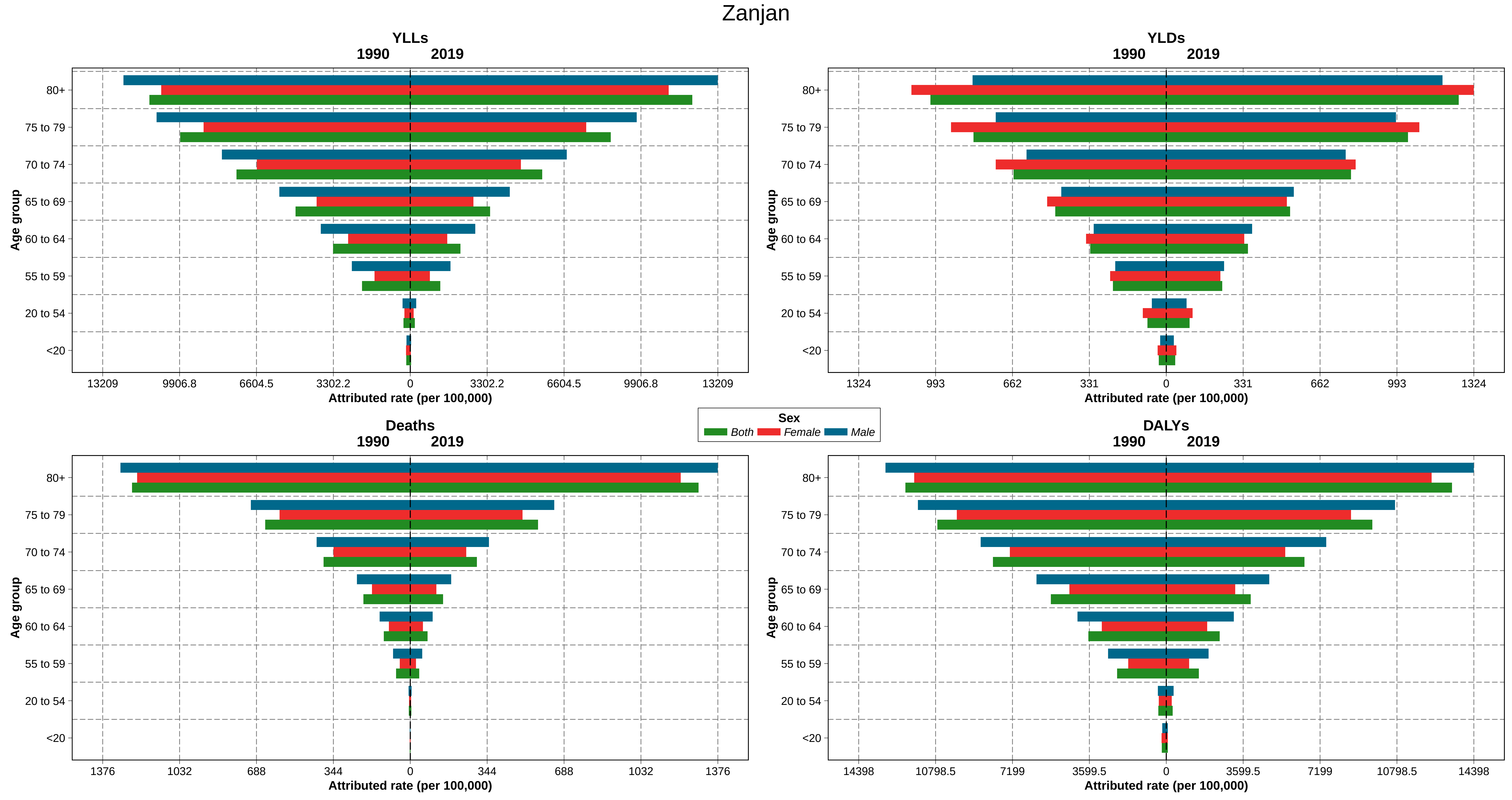

Zanjan
YLLs
1990
2019
80+
75 to 79
70 to 74
65 to 69
60 to 64
55 to 59
20 to 54
<20
Age group
13209
9906.8
6604.5
3302.2
0
3302.2
6604.5
9906.8
13209
Attributed rate (per 100,000)
YLDs
1990
2019
1324
993
662
331
0
331
662
993
1324
Attributed rate (per 100,000)
Sex
Both
Female
Male
Deaths
1990
2019
1376
1032
688
344
0
344
688
1032
1376
Attributed rate (per 100,000)
DALYs
1990
2019
14398
10798.5
7199
3599.5
0
3599.5
7199
10798.5
14398
Attributed rate (per 100,000)
