# Supplementary material for: Trends of national and sub-national burden attributed to kidney dysfunction risk factor in Iran: 1990-2019
Source: Front Endocrinol (Lausanne). 2023 Feb 27;14:1115833. doi: 10.3389/fendo.2023.1115833 (PMC10010168; doi:10.3389/fendo.2023.1115833)
Supplement: Supplementary file 9 [file DataSheet_1.docx]

### Providing data or critical feedback on data sources

Ashkan Abdollahi, Ali Ahmadi, Sepideh Ahmadi, Jalal Arabloo, Mohammad Arjomandzadegan, Seyyed Shamsadin Athari, Sara Bagherieh, Shirin Barati, Farshad Farzadfar, Ali Fatehizadeh, Maryam Gholamalizadeh, Kimiya Gohari, Hadi Hassankhani, Bagher Larijani, Mohammadreza Mobayen, Yousef Moradi, Mohsen Naghavi, Ghazaleh Pourali, Nazila Rezaei, Sahar Saeedi Moghaddam, Parnian Shobeiri, Majid Taheri, Amir Taherkhani, and Iman Zare.

### Developing methods or computational machinery

Farshad Farzadfar, Mohsen Naghavi, and Sahar Saeedi Moghaddam.

### Providing critical feedback on methods or results

Ashkan Abdollahi, Ali Ahmadi, Sudabeh Alatab, Jalal Arabloo, Mohammad Arjomandzadegan, Seyyed Shamsadin Athari, Sina Azadnajafabad, Mohammadreza Azangou-Khyavy, Nayereh Baghcheghi, Sara Bagherieh, Shirin Barati, Azizallah Dehghan, Farshad Farzadfar, Ali Fatehizadeh, Ali Gholami, Kimiya Gohari, Hadi Hassankhani, Mohammad Jokar, Mohammad Keykhaei, Fatemeh khorashadizadeh, Farzad Kompani, Hamid Reza Koohestani, Bagher Larijani, Ata Mahmoodpoor, Elaheh Malakan Rad, Mohammadreza Mobayen, Yousef Moradi, Negar Morovatdar, Mohsen Naghavi, Seyed Aria Nejadghaderi, Maryam Noori, Hassan Okati-Aliabad, Ghazaleh Pourali, Quinn Rafferty, Mahsa Rashidi, Mohammad-Mahdi Rashidi, Nazila Rezaei, Negar Rezaei, Sahar Saeedi Moghaddam, Parnian Shobeiri, Seyed Afshin Shorofi, Seyyed Mohammad Tabatabaei, Majid Taheri, Amir Taherkhani, Mazyar Zahir, and Moein Zangiabadian.

### Drafting the work or revising is critically for important intellectual content

Ashkan Abdollahi, Ali Ahmadi, Sepideh Ahmadi, Jalal Arabloo, Sina Azadnajafabad, Mohammadreza Azangou-Khyavy, Sara Bagherieh, Shirin Barati, Farshad Farzadfar, Ali Fatehizadeh, Fataneh Ghadirian, Kimiya Gohari, Farzad Kompani, Bagher Larijani, Soleiman Mahjoub, Ata Mahmoodpoor, Elaheh Malakan Rad, Mohammadreza Mobayen, Esmaeil Mohammadi, Yousef Moradi, Mohsen Naghavi, Seyed Aria Nejadghaderi, Ghazaleh Pourali, Sina Rashedi, Nazila Rezaei, Negar Rezaei, Sahar Saeedi Moghaddam, Amirhossein Sahebkar, Parnian Shobeiri, Seyed Afshin Shorofi, Amir Taherkhani, Mazyar Zahir, Moein Zangiabadian, and Iman Zare.

### Management of the overall research enterprise

Farshad Farzadfar, Bagher Larijani, Mohsen Naghavi, Seyed Aria Nejadghaderi, Negar Rezaei, and Sahar Saeedi Moghaddam.
